# Lithographic patterning of conformal thin films on 3D structures using Scaffold-architected Lift-off masks

**DOI:** 10.1038/s41467-026-75538-z

**Published:** 2026-07-14

**Authors:** Xinxin Liu, Zifan Che, Zofia Maj, Lee-Lun Lai, Kristinn B. Gylfason, Valentin Dubois, Shyamprasad N. Raja, Göran Stemme, Frank Niklaus

**Affiliations:** Department of Micro and Nanosystems (MST), School of Electrical Engineering and Computer Science (EECS), KTH Royal Institute of Technology, Stockholm, Sweden

**Keywords:** Surface patterning, Surface patterning, Design, synthesis and processing

## Abstract

Micro- and nanoscale patterning of conformal thin-film coatings on the exterior surfaces of complex three-dimensional (3D) structures is essential for emerging applications such as soft robotics, photonics, and functional 3D-printed MEMS devices. However, existing methods struggle to deliver high-resolution patterning on complex 3D structures and often suffer from poor thickness control, and inadequate surface conformity of the thin-film coatings. Here we present a robust approach for patterning of conformal thin-film coatings on complex 3D structures, including on sloped surfaces with angles up to 90°, with multiscale dimensions from 100 μm to 100 nm, and even down to the sub-30 nm scale when mask shrinkage techniques are used. This patterning approach utilizes a lithographically defined 3D Scaffold-Architected Lift-Off (SALO) mask in the lift-off process. It is agnostic to the used thin-film deposition process and enables even lift-off patterning of atomic layer deposited (ALD) conformal coatings, a task infeasible for conventional shadowing-based lift-off processes. Our approach opens opportunities for manufacturing complex 3D structures at the micro- and nanoscale by enabling lithographic patterning on the exterior surfaces of arbitrary 3D structures.

## Abstract (web summary)

Micro- and nanoscale patterning of conformal thin-film coatings on the exterior surfaces of complex structures is essential for emerging applications but existing methods struggle to deliver high-resolution patterning. Here the authors present a robust approach for patterning of conformal thin-film coatings on complex 3D structures with multiscale dimensions from 100 μm to down to the sub-30 nm scale.

## Introduction

Photolithographic processes on planar surfaces, including lift-off techniques, are widely used for the manufacturing of planar structures in applications such as micro- and nano-electronics, photonics, MEMS, and other micro- and nano-system components. However, an increasing number of emerging applications now demand three-dimensional (3D) geometries, where device performance is intrinsically linked to its 3D geometry. Notable examples include curved optical focal planes for enhanced field-of-view^1–3^, non-planar photonic metasurfaces that enable advanced light manipulation through tailored phase profiles^4–6^, fiber-tip integrated sensors that bring high-resolution sensing capabilities directly into confined or in vivo environments^7,8^, and 3D-printed functional MEMS^9–12^. Despite their promising applications, such 3D devices remain challenging to fabricate using conventional planar lithography, which suffers from degraded resolution and limited adaptability when applied to non-planar surfaces. Bridging this gap requires fabrication strategies that enable reliable and high-resolution patterning of conductive and dielectric thin-film coatings on exterior surfaces of complex 3D structures, thereby facilitating the realization of functional devices with arbitrary 3D topographies.

Previous efforts to realize patterned thin films on non-planar surfaces generally fall into three categories: (i) Direct lithography on 3D surfaces, (ii) transferring patterned layers onto 3D structures, and (iii) direct printing or writing of patterns onto 3D substrates. Each class of methods offers partial solutions yet suffers from distinct limitations that hinder broad applicability. (i) Direct lithography on non-planar surfaces involves adapting conventional lithographic techniques such as stencil lithography^13^, projection lithography^5,14^, soft lithography^15^, and nanoimprint lithography^1,16^, to the surfaces of 3D structures. These methods typically rely on 2D optical systems and can work reasonably well for realizing simple patterns. However, when extended to sharply curved or highly textured 3D geometries, they frequently encounter issues of pattern distortion and misalignment, undermining their reliability. To overcome these drawbacks, direct material removal techniques using high-energy beams have been explored previously, including laser ablation^17^ and focused ion beam (FIB) milling^18,19^. These methods can create high-resolution patterns directly on 3D substrates. However, these approaches often induce undesirable effects such as material damage in heat-affected areas, spallation, and unintended doping, which may compromise the intrinsic properties of the deposited thin films. (ii) Transfer or assembly of patterned layers onto 3D surfaces is another approach, and includes transfer printing^2,7,20,21^and mechanically guided assembly^22–24^. These substrate-mount approaches provide a lab-friendly and low-cost means to conform thin-film patterns onto curved surfaces through mechanical manipulation or reflow processes. Notably, reflow-assisted microprinting^20^ has enabled conformal transfers onto complex curvilinear geometries, however they are typically constrained to specific shapes (e.g., spherical surfaces), they struggle with precise alignment, and they risk damaging the patterned film during transfer, thereby limiting their reliability and versatility. (iii) Direct printing or writing methods are well suited for realizing precise patterns on 3D surfaces due to their high design freedom and direct-write capability. Early efforts using focused electron or ion beam-induced deposition (FEBID, FIBID)^25,26^ demonstrated high-resolution patterning on freeform surfaces, albeit limited to very small features and extremely slow throughput. Recent advances in direct ink writing processes^27–32^ have pushed the boundaries of resolution and complexity of the patterns that can be realized on complex 3D structures using these techniques. Furthermore, conductive and other functional inks have been applied to directly print 3D structures using femtosecond laser-based two-photon polymerization (TPP)^33–36^. Despite these achievements, such techniques remain largely limited to polymeric or metallic inks. While some attempts have been made to print inorganic materials^33,34^, the resulting materials still fall short of matching the electrical, thermal, and mechanical properties of thin films deposited by conventional techniques such as physical vapor deposition (PVD), chemical vapor deposition (CVD), or atomic layer deposition (ALD). Additionally, the additive nature of these processes inherently limits their thickness resolution. Taken together, existing methods for patterning thin-film coatings on non-planar 3D structures face fundamental trade-offs and limitations, including conformality of the coating on complex 3D geometries, material selection and quality, pattern resolution, thickness control, and uniformity of the thin-film coatings.

To address these limitations, here we report a patterning strategy that enables multi-scale lift-off of conformal thin films, including atomic layer deposited (ALD) films, on complex 3D topographies and structures, which is not possible using conventional lift-off processes. We demonstrate the capabilities of our approach by lift-off patterning of diverse thin films on a range of 3D structures, including silicon pyramids with steep 54.7° sidewalls, dome-shaped silicon structures, glass structures, and TPP-printed 3D polymer structures. Our approach uses TPP printing of sacrificial scaffolds as a lift-off mask, combined with a mechanically driven lift-off process using sonication in an ultrasonic bath. By purposefully designing 3D features in the scaffold mask that promote its mechanical detachment during lift-off, selective scaffold removal is achieved via controlled sonication, thereby enabling clean, damage-free patterning of conformal thin-film coatings on complex 3D topographies and structures. We term this method Scaffold-Architected Lift-Off (SALO). Prior studies have shown that ultrasonic agitation can assist the removal or detachment of TPP-printed microstructures, such as helical microswimmers from substrates^37^. Ultrasonic agitation has also been used to remove overhang supports of TPP-printed structures^38^, and to clean off arrays of printed polymer pillars from silicon surfaces^39^. However, in these reports, sonication is used only to separate or clean off polymer structures from substrate surfaces. In contrast, our SALO process utilizes TPP-printed scaffold masks with 3D design features that cause the scaffold to break and releases at predetermined release interfaces when exposed to sonication. This approach enables high-resolution lift-off patterning of conformal thin films, including ALD coatings. While ALD is well suited for coating the exterior surfaces of complex 3D structures because of the extremely uniform and pinhole-free surface coverage of ALD processes, the same coating conformality also closes the sidewall pathway of conventional lift-off resist masks that is critically important in solvent-based lift-off processes. One report has addressed this problem by employing a conventional lift-off mask and manually scratching openings in the deposited ALD film to provide solvent access to the underlying lift-off resist^40^, which however is not a scalable process. Another report has proposed the use of inkjet-printed growth-inhibiting surface layers that result in area-selective ALD and allow subsequent lift-off^41^, however this approach suffers from limited resolution in the micrometer range and is specific to the used ALD process. Thus, while SALO inherits the benefits of conventional planar lift-off processes, such as patterning of high-quality deposited thin-film materials and high pattern resolution, it overcomes important limitations, including the inability to pattern conformal thin-film coatings, e.g., ALD coatings, and making lift-off patterning applicable to coatings on arbitrary 3D topographies and structures.

## Results

### Patterning of thin-film coatings by Scaffold-architected Lift-off (SALO)

**Fig. 1:**
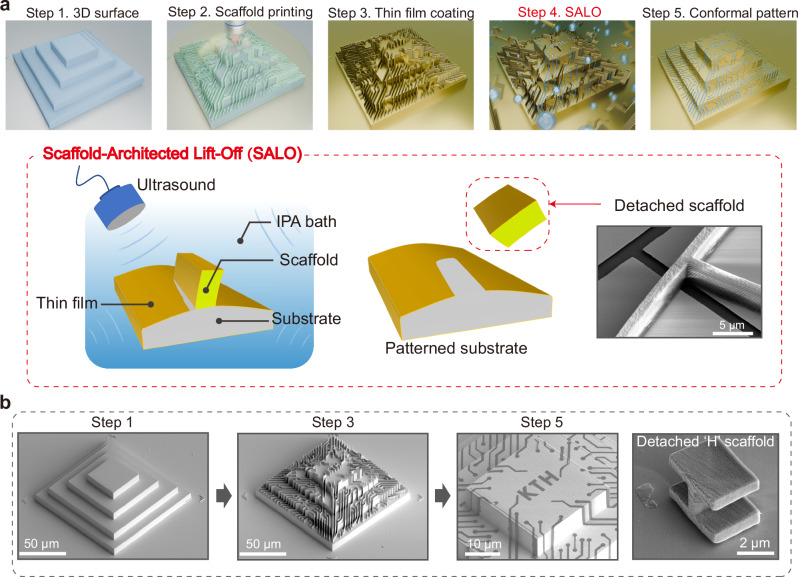
Scaffold-architected lift-off (SALO) process. **a** Schematic illustration of the SALO process steps (upper panel). Steps 1 and 2: Printing of the polymer scaffold structures by TPP. Step 3: Conformal thin-film coating of the surface. Step 4: Sonication to initiate scaffold lift-off. Step 5: Final pattern after SALO. In the SALO process (Step 4), the scaffold with the thin-film coating is selectively lifted off in an isopropanol bath (lower panel). The sonication causes cavitation-induced microjets and shockwaves that facilitate the mechanical detachment of the scaffold, leaving the defined pattern on the underlying surface. **b** SEM images of samples at each of the key SALO process steps. The 3D substrate and the scaffold structures were fabricated using TPP printing, demonstrating high-resolution patterning on complex 3D topographies.

The proposed SALO process (Fig. 1a) begins with the aligned printing of scaffold structures (i.e., the lift-off mask) directly onto the target surface of a 3D structure using TPP lithography. Therefore, we first drop-cast IP-Dip photoresist (Nanoscribe GmbH, Germany) onto the target surface, followed by scaffold printing (Fig. 1, Step 1–2). We selected the proprietary acrylic photoresist IP-Dip as scaffold mask material in our experiments because this resist is optimized for ultra-high-resolution 3D patterning in our employed TPP laser lithography tool, Photonic Professional GT2 system (Nanoscribe GmbH, Germany). Conveniently for the SALO workflow, IP-Dip exhibits intrinsically weak adhesion to untreated silicon and SiO_2_ surfaces^42^, a characteristic that facilitates clean and reproducible structural release during the final mechanical lift-off step without damaging the underlying surface or the deposited thin films. The entire sample is then coated conformally with the thin-film material to be patterned (Fig. 1, Step 3). The deposition of the thin-film coating can be done, for example, by physical vapor deposition, chemical vapor deposition, or atomic layer deposition. Finally, the sample is immersed in an ultrasonic bath filled with isopropyl alcohol (IPA), where mechanical forces induced by cavitation in the liquid selectively remove the scaffold along with the overlying thin film (Fig. 1, Step 4), revealing the patterned film on the underlying surface (Fig. 1, Step 5). Given appropriate designs of the lift-off mask scaffold and sonication parameters, the scaffold structures cleanly detach from the substrate during the mechanical lift-off step, resulting in well-defined patterns, as shown in the scanning electron microscope (SEM) image of Fig. 1a (lower panel). To demonstrate the feasibility of this approach, we patterned a 50 nm thick Ni film deposited by sputtering on a terraced 3D structure. The individual steps of the process are shown in Fig. 1b (see Methods for full details). High-fidelity patterns are achieved on both horizontal and vertical surfaces (Fig. 1b, Step 5), and the lifted-off scaffold with the “H” shape reveals the clean lift-off and detachment of the scaffold from the substrate.

The SALO process relies on efficient detachment of the lift-off mask (scaffold structures) from the substrate surface. When using sonication for the lift-off step, cavitation-induced shockwaves and microjets in the liquid dislodge the scaffold structures^38,43^. Here, we used isopropanol (IPA) liquid for the lift-off process, as it is particularly effective in promoting stable cavitation conditions due to its low surface tension and relatively low vapor pressure (~4 kPa at 20 °C, lower than most organic solvents) help to sustain stable cavitation during sonication. For the design of the scaffold structures, there are two important parameters that facilitate the detachment of the scaffolds from the surface during lift-off:

(1) Weak adhesion between the polymer scaffold and the surface on which the thin film is coated facilitates controlled and clean lift-off. If the substrate surface is a polymer (e.g., a TPP-printed 3D structure), adhesion between the polymer surface and the printed scaffold must be minimized. This can, for example, be achieved through surface treatments or by applying a thin intermediate layer on the surface prior to scaffold printing. We found that when printing the scaffold mask directly on a polymer surface, depositing a thin intermediate inorganic layer, such as 10 nm of sputtered SiO₂, is generally required, acting as a diffusion barrier and preventing strong covalent cross-linking and polymer chain interpenetration between the polymer surface and the IP-Dip resin^44^. This approach confines the lift-off mode of the scaffold mask to clean interfacial delamination rather than fracture within the scaffold itself. To evaluate the influence of different substrate surfaces and surface pre-treatments on the lift-off behavior of the scaffold mask, we performed comparative contact-angle and scaffold adhesion experiments and found that the surface material and its pre-treatment are important factors for reliable scaffold lift-off (Supplementary Note 1).

(2) Suitable designs of the scaffold structures facilitate controlled and clean detachment of the scaffold from the surface during SALO. This is the case for scaffold structures that feature narrow areas in which the scaffolds are attached to the substrate surface, combined with comparably large scaffold height (i.e., high-aspect ratios between the scaffold height (*h*) and the width (*w*) of the scaffold). For effective lift-off, scaffold designs should typically exhibit a height-to-width aspect ratio (*h*/*w*) greater than 1. For large-area patterns, hollow (shell-type^45^) scaffold designs can be used that are attached to the substrate surface only at the rim of the structure, thereby enabling an efficient lift-off process. TPP lithography enables printing of 3D scaffold structures with optimized designs, including shell-type structures. Unlike conventional planar lift-off, which relies on chemical or thermal means to remove the lift-off mask, the SALO process uses mechanical forces; thus, it avoids both the harsh solvents that can introduce chemical contamination and the ion bombardment damage typical of beam-based patterning methods. In addition, SALO enables lift‑off of conformally coated thin films, which conventional lift-off cannot achieve because solvent access to the resist sidewalls is required to initiate the lift-off process^40^. Furthermore, the crosslinked TPP photoresist used in the present study exhibits thermal stability up to 350 °C, allowing high-temperature deposition processes for the thin-film material while maintaining pattern fidelity of the lift-off mask.

### SALO process capabilities

To elucidate and demonstrate the capabilities of the SALO process, we conducted experiments addressing four key aspects: (1) Patterning of thin-film coatings on surfaces with diverse 3D topographies; (2) patterning on different substrate materials; (3) compatibility with different deposition methods (PVD and ALD); and (4) the achievable resolution of the resulting patterns.

**Fig. 2:**
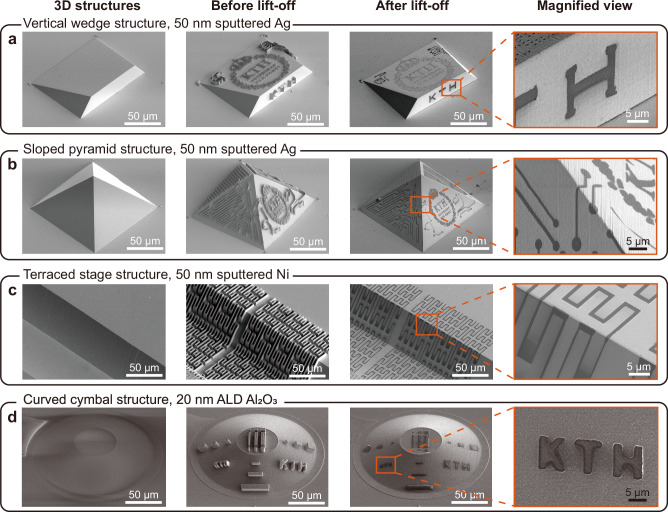
Patterning of conformal thin-film coatings on diverse 3D topographies. SEM images of the SALO process applied to different 3D structures and surface topographies, demonstrating conformal patterning across varying inclinations. Each row shows: the initial printed 3D structures (first column), the printed scaffold structure on the surfaces of the 3D structures with the thin-film coatings before lift-off (second column), the patterned thin-film coating after lift-off (third column), and a magnified view of a detail in the pattern (fourth column). Lift-off patterns on: **a** SALO on a 3D-printed vertical wedge surface using a sputtered Ag thin-film coating. **b** SALO on a 3D-printed sloped pyramid surface using a sputtered Ag thin-film coating. **c** SALO on a KOH-etched silicon terraced stage surface with high-density microstructures using a sputtered Ni thin-film coating. **d** SALO on a 3D-printed cymbal surface using a conformal ALD Al_2_O_3_ thin-film coating.

To explore the patterning of coatings on 3D structures with diverse topographies, we fabricated 3D structures with different shapes, angles, and topographies on different substrates (Fig. 2a, 2b, 2d: 3D-printed polymer and Fig. 2c: KOH-etched Si) using TPP. Therefore, we deposited a 10 nm thick conformal SiO_2_ layer on the 3D-printed samples using sputter deposition. This was done to provide a controlled and weak adhesion between the surfaces of the printed 3D structures and the printed scaffold (lift-off mask). Thereafter, we printed the scaffolds across the different surfaces of the 3D structures. After thin-film coating (Fig. 2a–d, second column), we performed the lift-off step under sonication in an IPA bath, resulting in patterned thin-film coatings across the surfaces of the 3D structures (Fig. 2a–d, third column). We used sputtering (ATC Orion-8, AJA international Inc., USA) of 50 nm thick Ag for the thin-film coatings of the 3D structures in Fig. 2a, 2b, and 50 nm thick Ni in Fig. 2c. As a versatile process for patterning thin-film coatings on the exterior surfaces of 3D structures with arbitrary topographies, SALO is compatible with a wide range of 3D substrate materials. To further demonstrate these capabilities, we patterned the United Nations logo onto substrates with curved surfaces (fabrication details in Methods–Preparation of 3D substrate), including on TPP-printed curved polymer structures and on curved silicon surfaces (Supplementary Fig. S2). When printing across complex surface topographies, the printed scaffold can be precisely positioned on the 3D surfaces with an alignment accuracy of approximately 1 μm (Supplementary Fig. S3), which is important to minimize pattern distortions in non-continuous regions of the 3D topography such as near edges.

As a 3D patterning technique, ALD is a preferred coating method because it produces highly conformal films with uniform and precisely controlled nanometer-scale thicknesses, even on complex topographies such as overhangs, re-entrant features, and capped cavities. Paradoxically, this very conformality makes ALD layers extremely difficult to pattern using conventional lift-off processes. To highlight the versatility of the SALO process in patterning conformal ALD coatings, we patterned a 20 nm thick ALD Al₂O₃ layer on curved cymbal structures (Fig. 2d). Furthermore, we patterned 20 nm thick ALD Al₂O₃ layers using arrays of scaffold mask structures with different complex overhang features (Supplementary Information, Fig. S4a). We also patterned a 20 nm thick ALD Al₂O₃ layer on large-area (mm-scale) arrays of 3D pillars (Supplementary Information, Fig. S4b). Our results show that localized interfacial debonding of the scaffold mask cleanly shears off the patterned features along the scaffold perimeter without transferring destructive tensile stresses to the surrounding thin film, thereby enabling the patterning of highly conformal ALD coatings with clean edges. and no evident cracking in the inspected cross-sections. This is further confirmed by SEM and FIB-milled cross-sectional analysis of a SALO-patterned 25 nm thick ALD ZnO layer (see Supplementary Fig. S5). To further confirm the actual thickness of a patterned ALD layer, we performed step-height measurements by atomic force microscopy (AFM) of the thinnest representative ALD layer (15 nm of HfO₂) that we patterned using SALO, confirming its nominal thickness (Supplementary Fig. S6c). When patterning over complex 3D topographies, deposition non-uniformity can become a critical consideration. For surface-reaction-limited deposition processes such as ALD, however, the coatings are highly uniform, even in between high-aspect-ratio scaffold geometries, as illustrated in Supplementary Fig. S6a, b. For more directional thin-film deposition processes such as PVD, potential geometric shadowing can be mitigated through scaffold geometry design, as discussed in Supplementary Note 6. Taken together, our results demonstrate that the SALO process is compatible with a wide range of thin-film deposition techniques and enables high- resolution patterning on sloped, stepped, and vertical surfaces (up to 90° angles) with excellent uniformity.

**Fig. 3:**
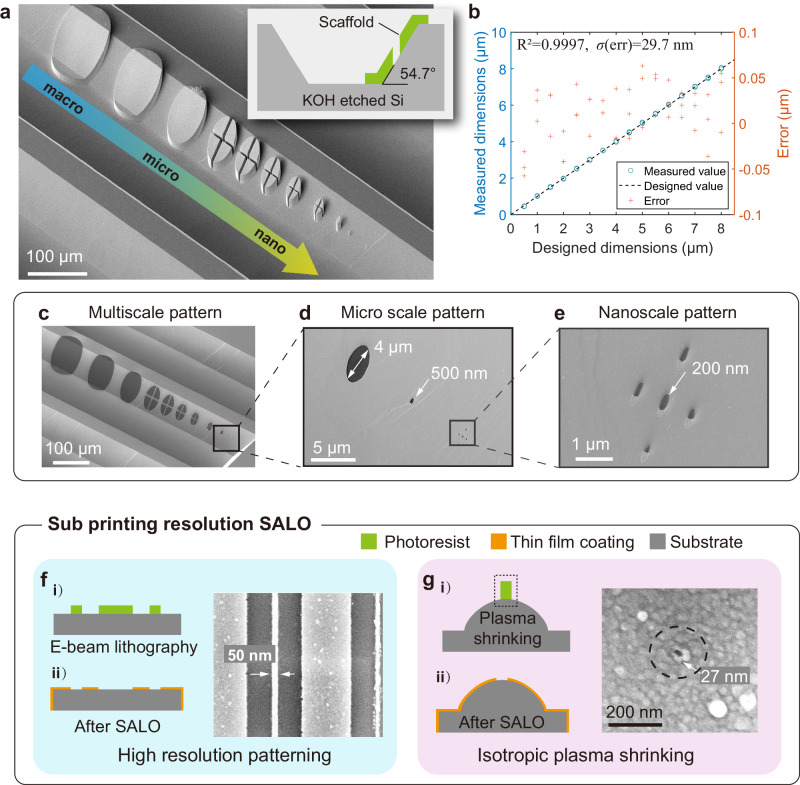
Multiscale patterning with critical dimensional features spanning three orders of magnitude. **a** Polymer scaffold for SALO with multiscale patterns ranging from 100 µm to 200 nm, printed on the KOH-etched silicon surfaces of a single substrate. A 100 nm thick Ag film was sputtered onto the scaffold, and the 3D structures were KOH-etched in a silicon substrate (100) (inset shows a schematic of the scaffold in the KOH-etched trench). **b** Measured dimensions of the resulting patterns in the 50 nm thick Ni film after SALO as a function of the designed dimensions of the scaffold, for pattern widths ranging from 500 nm to 8.5 µm. The errors were all within an 80 nm span, including SEM measurement errors. **c** SEM image showing the integrated multiscale patterns combining both nano- and microscale features. **d** SEM image showing a microscale pattern with feature dimensions of approximately 4 µm, next to patterns with nano-scale dimensions. **e** | SEM image showing nanoscale patterns in a 50 nm thick Ni film with minimum feature size of about 200 nm. **f** |High-resolution patterns in a 30 nm thick Au film with dimensions down to 50 nm, realized using an e-beam lithography-defined scaffold for the SALO process. **g** Pattern in a 30 nm thick Ag film with dimensions down to 27 nm, realized by TPP scaffolds in combination with O_2_-plasma shrinkage of the scaffolds. The dashed line indicates the scaffold outline before plasma shrinking. Source data are provided as a Source Data file.

As with any lithographic method, patterning resolution is an important consideration. The underlying lithography in our approach is based on TPP using a 780 nm laser light source, achieving sub-micrometer spatial resolution. Our SALO method enables multiscale patterning on the same substrate, with features spanning three orders of magnitude in size, from 100 µm down to sub-100 nm (Fig. 3). To demonstrate multiscale patterning capabilities, we designed and realized patterns in thin-film coatings on 3D sloped surfaces, featuring lateral dimensions ranging from 100 µm to 200 nm (Fig. 3a–e). Here we used a silicon substrate (100) in which we KOH-etched sloped 3D surfaces (Fig. 3a). We printed the scaffolds with multiscale dimensions on the KOH-etched silicon surfaces using a uniform exposure dose and scan speed (see Methods–Scaffold fabrication). After sputtering a 50 nm thick layer of Ni on the sample, we performed lift-off to define the patterned features with lateral sizes ranging from 100 µm to 200 nm. Dimensional analysis (Fig. 3b) revealed excellent size fidelity of the resulting patterns with a linear fit between designed and measured dimensions. Representative SEM images showing both microscale (Fig. 3d) and nanoscale (Fig. 3e) patterns further validated the pattern quality. The deviation between the designed and measured feature dimensions of the patterns remained below 80 nm across all scales, while manual errors in the SEM measurements likely contributed to these deviations. We further quantified pattern fidelity and feature-to-feature variation in sub-micrometer circular and square patterns fabricated in a 20 nm thick Cr layer on a glass substrate and found that features with nominal diameters down to 400 nm can be reproducibly defined, with measured standard deviations below 40 nm across the evaluated nominal dimensions (Supplementary Note 7 and Fig. S7). To benchmark pattern fidelity, we compared the line-edge roughness of patterns defined by the SALO process against those defined by an i-line lithography mask in combination with reactive ion etching (RIE) of the thin-film coating (Supplementary Fig. S8). Both methods yielded comparable 3σ line-edge roughness values of ~12 nm and similar power spectral density (PSD) profiles. This confirms that patterns defined by the SALO mask can achieve resolution and roughness metrics that are on par with those achievable by conventional planar lithography, even when the patterns are defined on non-planar surfaces.

To investigate if the SALO approach is compatible with lithographic techniques other than TPP, and to explore the resolution limits of patterns defined by the SALO mask, we used e-beam lithography (CSAR 6200.09 e-beam resist patterned by Voyager, Raith GmbH, Germany) to define high-aspect-ratio scaffolds for the SALO process (see details in Methods–Lithography process). This enabled us to pattern the thin-film coating with features as small as 50 nm (Fig. 3f), illustrating that the achievable resolution is determined primarily by the scaffold-defining lithography method. Additionally, by applying an isotropic O₂-plasma etch (Oxford Plasmalab 80 + RIE, 90 m Torr pressure, 10 W RF, 200 W ICP) to shrink the TPP-printed scaffold, we achieved features as small as 27 nm in a 30 nm thick Ag coating (Fig. 3g). It should be noted that the minimum feature size achievable by SALO depends on the patterning technique employed for realizing the scaffold mask. While electron beam lithography readily provides the possibility to realize scaffolds with sub-100 nm resolution on planar substrate surfaces, direct TPP printing of conformal scaffolds is typically limited to structures with smallest dimensions of around 200 nm. However, as demonstrated in the example in Fig. 3g, applying an oxygen plasma treatment to shrink the TPP printed scaffold mask enables lift-off patterning of sub-100 nm features on the surface of a 3D structure. These results highlight the compatibility of the SALO approach with different lithographic patterning tools, beyond TPP 3D printing, thereby underscoring its broad applicability, versatility, and scalability, with potential applications even in wafer-level micro and nanofabrication.

### Scaffold designs for efficient lift-off

**Fig. 4:**
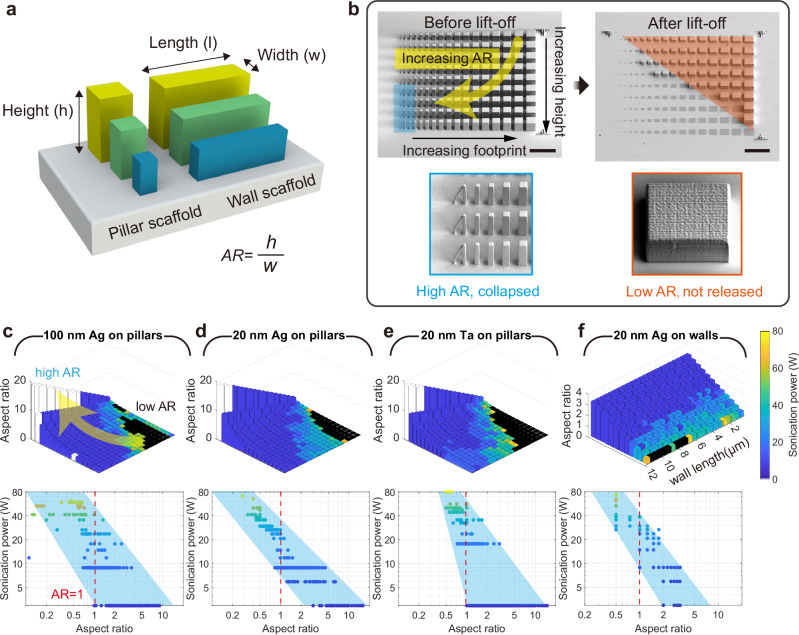
Scaffold (3D lift-off mask) designs for efficient lift-off. **a** Evaluation of scaffold designs consisting of pillar and wall structures with varying heights (*h*) and widths (*w*), on a planar silicon surface. **b** SEM of printed pillar scaffolds before and after lift-off, showcasing the effects of varying heights (*h*) and widths (*w*) and different *w*/*h* aspect ratios. The blue area indicates pillar scaffolds that collapsed before lift-off due to an excessively high-aspect ratio, while the orange area indicates pillar scaffolds that could not be lifted off even with the highest available ultrasonic power, as they did not have a sufficiently high-aspect ratio. Scale bar: 20 μm. *Top panel:* Lift-off statistics presented as 3D bar charts for pillar scaffolds with sputtered thin-film coating consisting of (**c**) 100 nm thick Ag, (**d**) 20 nm thick Ag, (**e**) 20 nm thick Ta, and (**f**) wall scaffolds with 20 nm thick Ag. The x and y-axes represent the positions of the evaluated scaffold structures in a and b, and the height of each bar represents the designed aspect ratio (height/width of side length of squared pillars; height/minimum width of walls). The color of each bar corresponds to the minimum ultrasonic power required for successful lift-off, as shown in the color map. *Bottom panel:* Scatter plots showing the experimentally determined ultrasonic power needed for lift-off as a function of the aspect ratio for the corresponding scaffold types in combination with the indicated thin-film coating. Each data point represents a different evaluated scaffold structure, and the red line indicates scaffold structures with an aspect ratio of 1. The blue shaded region illustrates a clear inverse correlation between scaffold aspect ratio and the required ultrasonic power for lift-off. Source data are provided as a Source Data file.

The aspect ratio of the scaffold features is a critical design parameter that facilitates successful SALO, which is defined here as the ratio between the height of a scaffold structure (*h*) and the width of the attachment area between the scaffold structure and the substrate surface (*w*) (Fig. 4a). During sonication in liquid IPA, cavitation occurs in a multi-bubble regime, generating microjets randomly throughout the medium^46^. As aspect ratios increase, mechanical leverage also increases, raising the likelihood of scaffold detachment at the scaffold–substrate interface under shock loading. The use of different designs for the scaffold structures that can or cannot be lifted off during the sonication step enables selective lift-off of pre-defined scaffold structures during SALO. To systematically explore the relation between the scaffold design and the lift-off of scaffold structures during SALO (i.e., the design space for suitable scaffold structures), we printed scaffold structures with varying designs on planar silicon 2D substrates and evaluated the ultrasound power required to lift-off the different scaffold designs. Therefore, we printed arrays of pillar and wall scaffold structures with varying heights (*h*), widths (*w*), and *h*/*w* aspect ratios using TPP 3D printing (Fig. 4a). By immersing the substrate with the scaffolds in an IPA bath and gradually increasing the ultrasonic power, we identified the minimum ultrasonic power needed to lift off each scaffold geometry and correlate this threshold with the aspect ratio of the respective scaffold structure. SEM images of pillar scaffolds before and after lift-off are shown in Fig. 4b. In this experiment, we observed three distinct regions with different behavior of the scaffold structures after printing and during sonication: (1) Scaffold structures with excessively high-aspect ratios collapse before sonication (blue area in Fig. 4b, white bar in Fig. 4c–f); (2) scaffold structures with intermediate aspect ratios are successfully and cleanly lifted off from the surface during sonication; (3) scaffold structures with too low aspect ratios remain adhered to the substrate surface after sonication (orange area in Fig. 4b, black bar in Fig. 4c–f). As the lift-off behavior of the scaffold structures also depends on the type and thickness of the thin-film coating that is patterned by the SALO process, we performed experiments to explore the design space for different scaffold designs using various thin-film coating materials and coating thicknesses (Fig. 4c–f), including pillars with a 100 nm thick Ag coating (Fig. 4c), pillars with a 20 nm thick Ag coating (Fig. 4d), pillars with a 20 nm thick Ta coating (Fig. 4e), and walls with a 20 nm thick Ag coating (Fig. 4f), all deposited by sputtering. The top panel in Fig. 4c–f presents 3D bar graphs where the y-axis denotes the h/w aspect ratio of the scaffold designs, and the color of the bars indicates the ultrasonic power required to lift off the respective scaffold structure. White bars represent collapsed scaffolds before sonication, while black bars indicate scaffold structures that failed to lift off. The bottom panel in Fig. 4c–f displays scatter plots of the lift-off power versus the h/w aspect ratio of the respective scaffold structure. In all cases, including the 100 nm thick Ag coating (Fig. 4c), the 20 nm thick Ag coating (Fig. 4d), the 20 nm thick Ta coating (Fig. 4e) on the pillar scaffolds, and the 20 nm thick Ag coating on the wall scaffolds (Fig. 4f), the results show a strong inverse relationship between h/w aspect ratio and the ultrasonic power required to lift off the scaffold structures. While thicker film coatings require slightly higher ultrasonic power to lift off scaffolds with identical designs, the overall trend remains consistent across different coating materials and scaffold designs. Notably, wall-formed scaffolds exhibit similar lift-off behavior as pillars, including the impact of the *h*/*w* aspect ratios of these scaffold structures, and the extended lengths of the wall-formed scaffolds have negligible impact on their lift-off behavior. Across all experiments, scaffold structures with h/w aspect ratios above 1 consistently enabled reliable lift-off (Fig. 4c–f (lower panel), marked by vertical red dashed lines in the scatter plots) using moderate ultrasonic power (~40 W). A summary of the critical detachment thresholds and geometric limits is provided in Supplementary Table S1 for rapid reference. These results also confirm that the SALO process can handle film thicknesses up to at least 100 nm with high fidelity. The results obtained with the circular scaffold are consistent with those for the rectangular-pillar scaffold (Supplementary Fig. S9). The localized structural damage and eventual lift-off of the pillars are most consistently explained by the bending moments induced by cavitation microjet impingement (Supplementary Fig. S10 and Note 10.1) This mechanical fracture mode is further supported by semi-quantitative finite element simulations, which align closely with the experimentally observed damage patterns (Supplementary Note 10.2).

For realizing patterns with large dimensions in thin-film coatings using the SALO process, large-area scaffold structures can be printed that are only partially attached to the substrate, thereby forming a shell structure (Supplementary Fig. S11). This can be achieved by TPP 3D printing of shell-type scaffold structures, in which the inner part of the printed polymer scaffold structure is not crosslinked and thus this inner part is not mechanically attached to the substrate surface. For such scaffold structures with only narrow attachments to the substrate surface along the periphery of the scaffold, effective lift-off is achieved during sonication, with similar lift-off behavior as wall scaffolds. Similar to the influence of the height-to-width aspect ratio in single-wall scaffolds, the lift-off behavior of shell-type scaffold structures is also governed by their geometric aspect ratio, specifically, the ratio between the scaffold height and the width of the outer rim in contact with the substrate. It should be noted that to ensure optimal lift-off reliability, shell-type scaffolds should be stored under yellow-light lithography or dark conditions prior to sonication. This prevents prolonged ambient ultraviolet (UV) light from inadvertently curing the trapped liquid resin. To evaluate the practical process window of the SALO process using shell-type scaffolds, we performed storage experiments of scaffold masks prior to performing the lift-off process (Supplementary Note 12), confirming that samples with shell-type scaffolds remained fully capable of successful lift-off even after 20 days of storage in a standard laboratory environment, provided they are shielded from direct sunlight. We found that exposure to direct sunlight can accelerate unintended curing of the resin inside the shell of the scaffold. Any polymer residue remaining after lift-off of the scaffold mask induced by prolonged exposure to ambient light can be removed using an O_2_ plasma cleaning step. To confirm that the SALO process and the subsequent cleaning procedure do not cause surface contaminations that could be problematic for subsequent processes, we used X-ray photoelectron spectroscopy (XPS) analysis and found no significant polymer contamination on the patterned surfaces (Supplementary Note 13).

Taken together, our findings highlight the critical role of the geometry of the scaffold structures in a SALO mask, and we established the aspect ratio between the height of the scaffold structure and the width of the attachment area between the scaffold and the substrate as a key design parameter to tune the lift-off efficiency across different scaffold designs, thin-film coating processes and thin-film coating thicknesses. The consistent, predictable behavior observed underscores the robustness and scalability of the SALO technique for patterning of thin-film coatings on the exterior surfaces of complex 3D structures.

### Multi-material patterning by sequential SALO and by selective SALO

To realize different patterned thin-film materials on the same substrate (multi-material patterning) using SALO, two approaches can be employed: (1) sequential lift-off and (2) selective lift-off.

**Fig. 5:**
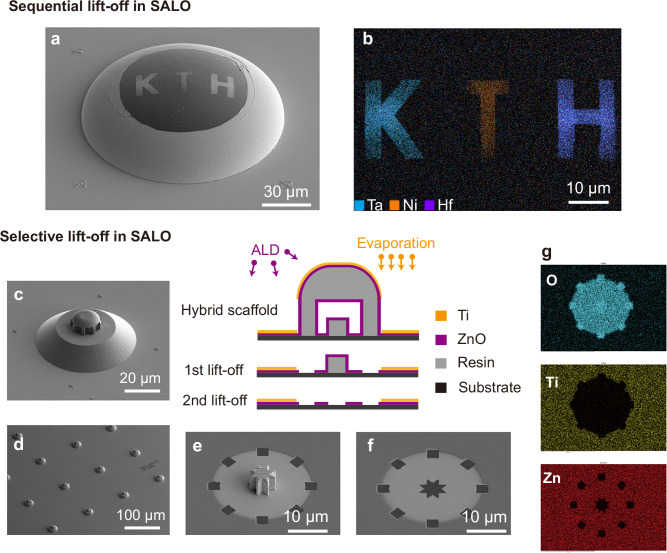
Sequential and selective SALO processes. **a**, **b** Sequential SALO patterning of multiple thin-film materials (Ta, Ni and Hf) on curved 3D surfaces (repeat scaffold printing, thin-film deposition, and lift-off). **a** SEM image of a 3D surface with the patterned letters ‘KTH’, each letter patterned in a different thin-film material. **b** Corresponding EDS map showing that each letter consists of a different thin-film material (Ta (cyan) for “K”, Ni (orange) for “T”, and Hf (purple) for “H”). **c** Selective SALO using hybrid scaffolds with different aspect ratios, combined with a conformal (ALD) and a directional (evaporation) coating. Sequential lift-off is achieved by selectively removing scaffolds of different aspect ratios by tuning the sonication power. **d** Hybrid scaffold array. **e** Pattern after the 1st lift-off. **f** pattern after 2nd lift-off. **g** EDX maps for O, Ti and Zn confirm the patterned SiO₂ substrate, 20 nm evaporation Ti layer and 20 nm ALD ZnO layer, respectively.

In (1) the sequential SALO approach, multiple thin-film coatings are patterned sequentially on the surface of a 3D structure, with the subsequent patterns aligned to the already patterned structures (Fig. 5a). To demonstrate this approach, we used a hollow shell-type scaffold design to facilitate large-area lift-off (see Supplementary Fig. S11 for details on the scaffold fabrication process). Using sequential SALO, we sputtered and patterned first a 30 nm thick layer of Ni, then a 30 nm thick layer of Ta, and then a 30 nm thick layer of Hf. Elemental mapping via EDS confirmed the presence of the Ta (cyan), Ni (orange), and Hf (purple) layers in the multi-material pattern (Fig. 5b). This repeated lift-off sequence highlights the compatibility of our SALO process with sequential lift-off patterning of multiple thin-film coating materials, including metals and dielectrics.

In (2) the selective SALO approach, pre-defined regions can be selectively lifted off and patterned while other defined regions of the same scaffold mask do not lift off. This is achieved by using different ultrasound thresholds for lifting off different parts of the scaffold mask (Fig. 5c–f). Thus, different masking regions can be released and patterned in a defined order, enabling the addition of a second thin-film material using a single patterning step. This lowers overlay error between the patterns in the two materials, protects delicate 3D topography, shortens process time, and lets the user define the order of materials in a stack without the need for sequential patterning. To demonstrate selective SALO on 3D topographies, we realized a mask with different types of scaffolds that release at different ultrasonic power thresholds (Fig. 5c, d). In this hybrid scaffold (Fig. 5c), scaffolds of different aspect ratios respond to different ultrasonic powers: The upper cap scaffold, having a smaller contact area and higher susceptibility to bending moments, detaches at lower ultrasonic power, while the underlying pillar scaffold remains intact. We demonstrate sequential SALO release with two deposited thin films and one single exposure (Figs. 5d–5f). The hybrid scaffold mask contained two types of scaffolds with two different aspect ratios in a single mask (Fig. 5d). We coated the mask first with a 20 nm thick conformal ZnO ALD coating and then with a 20 nm tick Ti layer using a directional evaporation step (Fig. 5c). In an initial lift-off step, low-power sonication removes only the cap scaffolds (Fig. 5e), thereby patterning part of the ZnO ALD coating and the Ti layer, while the pillar scaffolds remain. Next, a higher-power sonication step removed the remaining pillar scaffolds (Fig. 5f), thereby patterning the remaining part of the ZnO ALD coating. EDX elemental maps of O, Ti, and Zn confirmed the material-specific patterns (Fig. 5g). This process can also be used to deposit and pattern an additional thin firm coating in between the two selective sonication steps (not shown). Thus, the staged selective SALO approach enables a “single scaffold pattern, multiple layers lift-off”, featuring build-in self-alignment of the two material patterns and thereby reducing cumulative overlay alignment errors, and shortening process cycle time.

Taken together, our results here underscore the power of SALO as a platform for customizable, high-resolution, and material-agnostic patterning across complex 3D topographies. The ability to selectively integrate and pattern multiple materials in a layers-by-layer fashion opens opportunities for realizing functional 3D-printed nanodevices, photonic structures, and multifunctional 3D structures with engineered surface functionalities.

### Realization of functional structures

To demonstrate the versatility of the SALO process in fabricating functional structures, we realized two example devices featuring micro- and nanoscale dimensions: (1) Metal nanowires on 3D structures; and (2) optical wire-grid gratings patterned on an optical fiber tip. Metal nanowires patterned on 3D structures are promising candidates for applications in resistive strain sensors and superconducting nanowire single-photon detectors (SNSPDs), where integration on non-planar surfaces could enable electrical and optical coupling schemes and device architectures. Wire-grid gratings integrated on fiber tips have broad applications in compact polarization optics, wavelength filters, and refractive index sensors, particularly in biomedical or remote-sensing environments where miniaturization and conformal integration are essential.

**Fig. 6:**
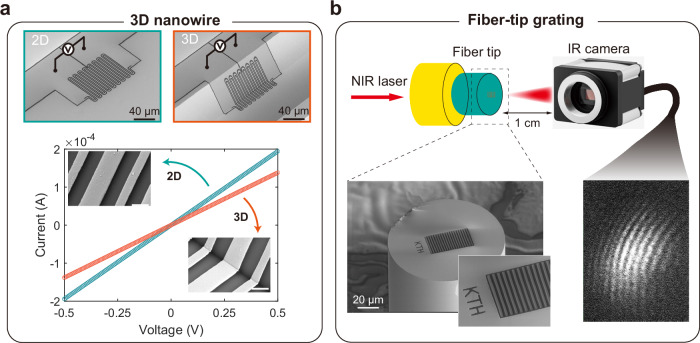
Electrical and optical characterization of 3D nanowires and fiber-tip gratings. **a** Measured current-voltage. (*I*–*V*) curves of nanowires patterned on both 2D (left/teal) and 3D (right/orange) surfaces. The insets show SEM images of representative nanowires on a planar substrate (left, teal box) and on the surface of a 3D structure (right, orange box) (scale bar: 1 µm). The evaluated nanowires had equal length and consisted of a 5 nm thick layer of Cr and a 90 nm thick layer of Au. The *I*–*V* curves verify the electrical conductivity of the nanowires. **b** Characterization of a fiber-tip grating. The top schematic illustrates the experimental setup where a near-infrared (NIR) laser is directed onto the grating fabricated on the tip of an optical fiber. An infrared (IR) camera captures the resulting diffraction pattern placed 1 cm away from fiber-tip surface with the grating. The SEM image (left) shows the fabricated grating on the fiber tip (scale bar: 20 µm; inset shows a closer view), and the diffraction image (right) displays the resulting diffraction pattern, indicating correct function of the fabricated grating on the fiber tip. Source data are provided as a Source Data file.

For the nanowire demonstration we patterned a gold nanowire on a silicon substrate surface with sloped sidewalls by KOH etching (slope sidewall angle of 54.7°). The sputtered nanowire, consisting of a 5 nm thick Cr adhesion layer and a 90 nm thick Au layer features a width of 900 nm and a total length of 1080 µm and is placed on the sloped surface and crossing the sharp edge of the sloped surface (Fig. 6a, right image). We performed electrical measurements of the nanowire via a voltage sweep from –500 mV to +500 mV. The nanowire exhibited a resistance of 3.01 kΩ (equivalent to 6.2 Ω·m), slightly higher than the 2.58 kΩ (equivalent to 5.2 Ω·m) of a reference sample consisting of an identical nanowire design placed on a planar surface (Fig. 6a, left image). Both current-voltage (I–V) curves showed good linearity, indicating reliable film continuity and electrical contacts, and the measured characteristics are consistent with those of comparable nanowire devices reported in the literature^47^^,^. The increased resistance in the 3D nanowire is likely due to minor non-uniformities in coating thickness or roughness near sharp edges, which could be further mitigated by improving the etching smoothness of the underlying silicon structures.

For the demonstration of the optical wire-grid gratings patterned on an optical fiber tip (Fig. 6b), we used a single-mode optical fiber with a 125 µm cladding diameter (Thorlabs, Inc., USA). We patterned a 30 nm thick Au grating using ion sputtering (SEC Co., Ltd., Korea), featuring a pitch of 3 µm and a linewidth of approximately 1.5 µm conformally on the fiber end. We carried out the optical characterization of the grating by inserting light in the fiber using a near-infrared (NIR) laser diode with a wavelength of 1550 nm, with the diffraction pattern of the light exiting the fiber tip being recorded by an NIR camera placed 1 cm in front of the fiber tip. The resulting pattern exhibited a curvature consistent with results from our simulations (Supplementary Fig. S14), confirming the structural and optical fidelity of the patterned grating. The optical response of the wire-grid gratings also is consistent with the behavior of comparable devices reported in the literature^48^. Beyond this specific demonstration, 3D photonics appears to be a particularly promising application space for SALO; for instance, ALD has previously been integrated with 3D-printed micro-optics to produce highly conformal anti-reflective coatings without compromising the underlying surface topography^49^. Combined with SALO, this conformal coating capability could enable the fabrication of more advanced 3D photonic architectures.

Together, our electrical and optical demonstrators establish the SALO process as a viable route for fabricating functional, high-resolution devices. Nanowire resistance measurements and optical diffraction patterns confirm the expected electrical and optical performance, underscoring the validity of the approach as a proof of concept. Beyond these demonstrations, our method is readily extendable to a wide range of photonic, electronic, and sensing devices requiring reliable patterning of conformal thin films on arbitrarily shaped 3D structures.

## Discussion

We established Scaffold-Architected Lift-Off (SALO) as a general method that enables lithographic lift-off patterning of conformal thin films on intricate geometries and the exterior surfaces of complex 3D structures. The SALO method utilizes the flexibility of 3D printing for sacrificial scaffold fabrication and a controlled mechanical lift-off process, enabling the patterning of a diverse range of coated thin-film materials. SALO is suitable for patterning thin-film coatings deposited by ALD, PVD, and related deposition methods that provide nm-scale thickness control of the deposited material. We also demonstrated the utility of the SALO process for creating functional structures by realizing metal nanowires and fiber-tip gratings, showcasing its versatility, precision and reliability. We evaluated SALO against two practically relevant characteristics for patterning on non-planar substrates and 3D topographies: Pattern flexibility and preservation of thin-film quality. Here, pattern flexibility refers to the ability to define target linewidths and spacings with maintained registration across curved and stepped surfaces, while achieving continuous coverage of the functional film. Preservation of thin-film quality means that the patterned thin films retain their as-deposited properties, without degradation from chemical, thermal, or irradiation processes during patterning. Conventional approaches that combine lithography with thin-film etching or high-energy beam sculpting often improve one of these characteristics at the expense of the other, for example, sacrificing coverage uniformity or degrading film properties during etching steps. In our implementation, the SALO process maintains film integrity because pattern definition and release do not expose the functional layers to energetic etchants or direct ion beams. The scaffold mask can be written by two-photon polymerization or electron beam lithography, enabling multiscale patterning from millimeter to nanometer ranges on curved and stepped surfaces. By repeating the scaffold, deposit, and release cycle, SALO produces sequential stacks with clean interfaces and conformal coverage on high-curvature features without intermediate etch steps. Additionally, hybrid scaffold designs further enable selective lift-off, which makes it possible to produce complex multi-material layouts on 2D and 3D geometries in a single exposure.

In this work, we successfully demonstrated the SALO process on a diverse range of substrates, including silicon surfaces with planar and 3D topographies, glass domes, TPP-printed 3D polymer structures, and optical glass fiber facets. While this highlights the broad applicability of the SALO patterning process, the technique is naturally constrained by the physical requirements of the TPP printing and lift-off workflows. Specifically, the TPP printing relies on precise laser focusing, meaning that substrates with extreme optical properties, such as highly reflective metal or excessively rough surfaces, can cause laser light scattering or interference artifacts that locally modify the exposure dose during TPP printing. Additionally, the substrate surface must possess sufficient mechanical robustness to withstand the ultrasonic agitation required during the lift-off process. As a result, applying SALO to highly reflective or rough surfaces, or to unconventional or fragile materials like soft polymers or highly porous substrates, will require process optimization. A summary of potential failure modes arising from poor adhesion of the deposited thin films, reflection-induced exposure non-uniformity when TPP printing the scaffold mask, and incomplete resist development of the TPP-printed scaffold mask is provided in Supplementary Note 15 and Fig. S15.

Taken together, SALO overcomes key limitations of existing conformal patterning approaches for 3D structures and presents a significant advancement in lithographic patterning of thin-film coatings on the exterior surfaces of 3D structures. To accommodate broader process windows (e.g., increased deposition temperatures), the organic photoresist for the scaffold mask may be replaced by hybrid organic-inorganic photoresists, which have been shown to offer improved thermal and mechanical rigidity^50,51^. Building on this, the SALO process could be further expanded by incorporating thermal treatments, such as calcination or pyrolysis^52^, of the 3D scaffold mask. This may enable more thermally robust scaffold architectures, provide a route towards further improving the achievable scaffold mask resolution, and facilitate the realization of multi-compositional 3D microstructures.

While printing the scaffold masks in the current work typically takes of the order of tens of minutes (for exact printing times see Supplementary Note 16). Scaffold mask writing by TPP can be readily scaled to speeds that are feasible for printing on wafer-scale areas using emerging high-throughput printing strategies based on parallelized laser beam generation^53–55^. Most recently, metalens-array-enabled TPP printing with spatially adaptive illumination has pushed parallel laser writing beyond ${10}^{8}$ voxels ${{{{\rm{s}}}}}^{-1}$^53^, making this approach in principle feasible for wafer-scale and industrially relevant applications. By leveraging these readily available nanofabrication tools, we anticipate that wide adoption and exploration of SALO will pave the way for possibilities in fabricating sophisticated 3D micro- and nanostructures for diverse scientific and industrial applications.

## Methods

### Preparation of 3D silicon substrates

Angled Sidewall Structures in Figs. 2c, 3a, and 6a:

To fabricate 3D silicon surfaces with sloped sidewalls, a 100 nm thick, low-stress silicon nitride (Si_3_N_4_) layer was deposited onto a standard silicon wafer via plasma-enhanced chemical vapor deposition (PECVD; P5000, Applied Materials, USA). A 1.3 µm thick layer of S1813 photoresist (MicroChem, USA) was spin-coated and patterned using a maskless aligner (MLA150, Heidelberg Instruments) with an exposure dose of 250 mJ/cm². Development was performed in MF-319 developer (MicroChem, USA) for 30 s. The pattern was transferred to the Si_3_N_4_ layer using PlasmaPro 100 Cobra ICP-RIE system (Oxford Instruments, UK) with a C₄F₈/O₂ plasma at an etch rate of ~310 nm/min. After stripping the residual resist, the wafer was immersed in 5% tetramethylammonium hydroxide (TMAH) at 80 °C for 1 h to etch the exposed silicon. This wet etch yielded ~60 µm deep cavities with 54.7° angled sidewalls, corresponding to the crystallographic (111) planes (Fig. 3a). Finally, the remaining Si_3_N_4_ mask was removed in 7% buffered hydrofluoric acid (BHF) for 20 min.

#### Dome structures in Fig. S2c and S2d

For dome-shaped silicon microstructures, a 5 µm thick layer of ma-P 1240 photoresist (Micro Resist Technology GmbH, Germany) was spin-coated at 1000 rpm and soft-baked at 105 °C for 5 min. Patterning was performed with the MLA150 system using an exposure dose of 1000 mJ/cm², followed by development in ma-D 331 developer (Micro Resist Technology GmbH, Germany) for 70 s. To form hemispherical profiles, the developed resist was reflowed on a hotplate at 220 °C for 1 min. The resulting resist domes were used as etch masks in an SF₆/O₂ plasma (15/20 sccm) in the PlasmaPro 100 Cobra ICP-RIE system (Oxford Instruments, UK). The etching of the silicon surface with the resist domes was performed at 30 W RF power, 600 W ICP power, and 6 mTorr chamber pressure for 20 min. Afterwards, the resist was stripped, yielding dome-shaped silicon features approximately 100 µm in diameter and 12 µm in height.

### Scaffold mask fabrication

Prior to scaffold printing, all substrates were cleaned using oxygen plasma (PlasmaPro 100 Cobra, 20 W RF) for 5 min to ensure surface cleanliness and activation. The scaffold structures, both solid and shell-type, and the 3D polymer structures in this work were printed via two-photon lithography using a commercial laser direct writing system (Photonic Professional GT2, Nanoscribe GmbH) equipped with a 780 nm wavelength femtosecond laser. The laser operates at a repetition rate of 80 MHz with a pulse duration of ~100 fs. A 63× oil-immersion objective with a numerical aperture (NA) of 1.4 was used to focus the laser beam. According to the diffraction limit, the diffraction-limited spot size is ~0.34 μm (estimated as 0.61λ/NA). In this study, an laser power of 20 mW was applied, which corresponds to a pulse energy of ~0.25 nJ according to the irradiance calculation^52,56^. Consequently, the peak intensity (irradiance) at the center of the focal spot is estimated to be approximately 1.4 TW/cm². Solid scaffolds were printed with dimensions comparable to the target structures. For larger features with x/y dimensions >10 µm, as shown in Figs. 5a and S11, shell-type scaffolds with a wall thickness of 3 µm, dome thickness of 2 µm and wall height of 5 µm were used to reduce printing time while maintaining lift-off capability. Shell-type scaffolds contain uncured photoresist and should be protected from ambient light to ensure clean removal during SALO. Otherwise, oxygen plasma may be needed to eliminate hardened residues. Solid structures were printed with a laser power of 20 mW, and 1000 µm/s laser scan speed, whereas shell-type scaffolds were printed at 10,000 µm/s. For finer features, the laser power was adjusted accordingly, while maintaining the laser scan speed. Only solid scaffolds underwent a UV flood exposure for 15 min.

### Preparation of 3D polymer structures

Fused silica substrates (25 mm × 25 mm × 0.7 mm) were used for direct laser writing of polymer scaffolds. All the 3D polymer structures (Figs. 1, 2a, 2b, 2d, 5a, and Figs. S2a, S2b, S4b, S11) were first treated with oxygen plasma to remove organic residues and introduce surface hydroxyl groups. To promote adhesion between the printed polymer structures and the substrate, silanization was performed using a 3-aminopropyltriethoxysilane (APTES, Aldrich Chemistry, USA) solution (50 mL ethanol + 500 µL APTES). Substrates were immersed in the solution for 30 min at room temperature, rinsed with isopropanol to remove excess silane, and dried under a nitrogen stream.

A drop of IP-Dip photoresist (Nanoscribe GmbH, Germany) was then deposited onto the silanized surface. 3D structures were printed using a Nanoscribe PPGT2 system at 20 mW laser power and 10,000 µm/s scan speed. Post-print development was performed by tilting the sample and immersing it in propylene glycol monomethyl ether acetate (PGMEA) for ~13 min, followed by rinsing in isopropanol for ~3 min. A final UV flood exposure of 15 min (irradiance at the sample plane was measured to be 14 mW/cm², corresponding to a total exposure dose of 12.6 J/cm²) ensured complete polymerization and solvent removal.

### Lithography process

E-beam lithography (Fig. 3f):

A positive-tone resist CSAR 6200.09 (Allresist GmbH, Germany) was spin-coated at 3000 rpm to obtain a ~200 nm thick resist layer, soft-baked at 180 °C for 3 min, and patterned using a Voyager e-beam lithography system (Raith GmbH, Germany), dose of 308 μC/cm^2^, 30 kV. Development was carried out in AR 600–546 (1 min) followed by a 20 s IPA rinse.

i-line lithography (Fig. S4):

S1813 photoresist (MicroChem, USA) was spin-coated at 4000 rpm and soft-baked at 110 °C for 1 min. Exposure was performed using a maskless aligner (MLA150, Heidelberg Instruments, Germany) at a dose of 250 mJ/cm². The resist was developed in MF-319 (MicroChem, USA) for 25 s, followed by a DI water rinse and nitrogen drying.

### Sonication-Based Lift-Off

Lift-off experiments were conducted using a benchtop ultrasonic bath (VWR USC300D, Avantor, Inc., USA) operating at 45 kHz. The bath provides nine discrete power settings from 10% to 100% of a nominal maximum electrical output power of 80 W. Because the instrument reports power as a percentage, all sonication conditions are reported as the selected setting (%) rather than an assumed delivered acoustic power (W). Sonication was performed in IPA at room temperature.

Unless otherwise stated, the ultrasonic bath was filled with IPA to a fixed liquid volume of 20 mL and allowed to equilibrate to room temperature (~23 °C) before use. Samples were immersed in a glass beaker containing IPA and placed at the center of the bath, with the beaker supported on a holder so that the sample is not in direct contact with the bottom of the bath. IPA was used as received, non-degassed prior to use, across all experiments. The bath temperature was monitored during sonication (monitored at the start and end of each 2 min sonication interval) and maintained within 22–27 °C.

For systematic evaluation of scaffold lift-off thresholds, the scaffold structures were sonicated at fixed power levels for 2 min, repeated 3 times to mitigate standing wave effects in the ultrasonic bath and ensure consistent exposure of the samples. For general lift-off demonstrations, a fixed power setting of 40 W for 5 min was sufficient to lift off most scaffold structures. Optical microscopy was used to monitor the process, and results of the final structures were confirmed via SEM imaging.

### Finite element simulations

Stationary finite element simulations were performed using COMSOL Multiphysics 6.4 (COMSOL AB, Sweden). Each scaffold pillar was modeled as a square cross-section cantilever beam with a fixed base footprint of 5 × 5 µm² and varying height to systematically sweep the aspect ratio (AR = h/w). The pillar material was assigned the linear elastic properties of IP-Dip photoresist, with a Young’s modulus of 2.9 GPa and a Poisson’s ratio of 0.38^57^, as specified by the manufacturer. A uniform pressure load was applied to one lateral face of the pillar to represent the equivalent hydrodynamic loading from cavitation microjet impingement. The base of the pillar was assigned a fixed boundary condition to represent the rigid substrate interface. The von Mises stress was spatially averaged over the entire basal cross-section and used as the metric for evaluating interface detachment. A free tetrahedral mesh with a maximum element size of 1 µm was used throughout the pillar volume to ensure sufficient spatial resolution, especially at the interface region between the pillar and the substrate.

## Supplementary information

Supplementary Information

Transparent Peer Review file

## Source data

Source Data

## Data Availability

The data that support the plots within this paper and other findings of this study are available from the corresponding author upon request. Source data are provided with this paper.
