# Supplementary material for: Lithographic patterning of conformal thin films on 3D structures using Scaffold-architected Lift-off masks
Source: Nat Commun. 2026 Jul 14;17:6201. doi: 10.1038/s41467-026-75538-z (PMC13369500; doi:10.1038/s41467-026-75538-z)
Supplement: Supplementary file 1 — Supplementary Information [file 41467_2026_75538_MOESM1_ESM.pdf]

Supplementary information

Lithographic Patterning of Conformal Thin Films on 3D Structures Using Scaffold-Architected Lift-Off Masks

Xinxin Liu[1], Zifan Che[1], Zofia Maj[1], Lee-Lun Lai[1], Kristinn B. Gylfason[1], Valentin Dubois[1], Shyamprasad N. Raja[1], Göran Stemme[1], and Frank Niklaus*[1]

[1]Department of Micro and Nanosystems (MST), School of Electrical Engineering and Computer Science (EECS), KTH Royal Institute of Technology, Stockholm 11423, Sweden

*Corresponding author: Frank Niklaus, frank@kth.se

Table of Contents:

Supplementary Note 1-16
Supplementary Fig. 1-15
Supplementary Table 1-2
Supplementary References

Supplementary Note 1. Influence of substrate surface and surface pre-treatment on scaffold mask lift-off

To evaluate the influence of different substrate surfaces on the lift-off behavior of the scaffold structures, we compared representative interfaces with different pre-treatments and chemistries, including O_2 plasma-cleaned glass, SiO_2-coated glass, TPP-printed polymer (IP-Dip), and silanized glass (Fig. S1). For each case, we examined both the water contact angle before scaffold deposition and the resulting lift-off behavior during sonication. O_2 plasma-cleaned and SiO_2-coated surfaces with low water contact angles of ≤10° enabled clean interfacial detachment of the scaffold, whereas silanized surface (contact angle of ~50°) and printed polymer surfaces (contact angle of ~80°) featured much stronger scaffold adhesion and led to fracture of the scaffold near its base during sonication, rather than clean removal of the scaffold from the surface. These results indicate that the scaffold lift-off mode is governed primarily by the interfacial adhesion state between the scaffold and the surface material, rather than by macroscopic wettability alone, which agrees with previous studies by Izard et al. and Jelinek et al[1,2]. The observed strong scaffold adhesion on silanized surface and polymer surfaces is consistent with stronger interfacial interactions, potentially including covalent anchoring in the case of silanized surfaces and polymer-polymer interpenetration or photochemically induced coupling in the case of printed polymer surfaces[3]. The resulting interfacial bond strength can exceed the cohesive strength of the scaffold material, shifting the lift-off mode from interfacial debonding between the scaffold and the substrate surface, to structural fracture of the scaffold material near the scaffold base. Therefore, on polymer surfaces we used a deposited thin intermediate SiO_2 layer before scaffold printing to ensure well-defined scaffold lift-off.

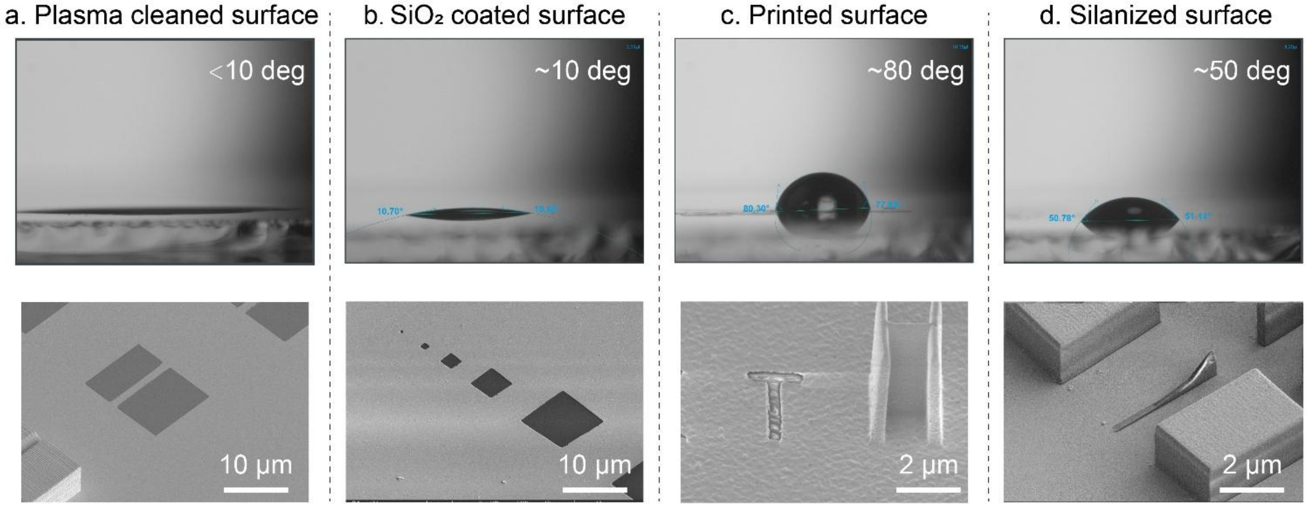

Supplementary Fig. S1. Correlation between substrate wettability and SALO behavior. Water contact angles were measured on four different surfaces based on ITO-coated glass substrate: **a** | O_2 plasma-cleaned surface, <10°. **b** | SiO_2-coated surface, ~10°. **c** | 1 μm-thick TPP-printed polymer surface (IP-Dip), as printed, ~80°. **d** | Silanized surface, ~50°. For (a) and (b), solid scaffolds with an aspect ratio (AR) of ~1 detached cleanly during sonication. For (c) and (d), solid scaffolds with an AR of ~1 did not release, while solid scaffolds with an AR of ~8 fractured at the base during sonication, instead of detaching from the substrate. Upper panels show the water droplets during contact-angle measurements, and lower panels show the corresponding state of the scaffolds after sonication. The features visible in the two lower-right panels are fractured scaffold remnants remaining on the substrate after lift-off.

Supplementary Note 2. Patterning on curved surfaces

The thin film coatings in these experiments consisted of 50 nm thick sputtered Ni. For the glass substrate with the curved 3D structure consisting of polymer resin (the same material as the 3D printed scaffold), a 10 nm thick interlayer of SiO_2 was applied before the scaffold printing to weaken adhesion of the scaffold to the surface and prevent covalent bonding between the polymer surface and the printed scaffold, thereby enabling clean detachment and lift-off of the scaffold structures during sonication. In both cases, the SALO process produced high-resolution, high-fidelity thin film patterns that conformed to the underlying 3D surfaces.

The dome-like silicon surfaces shown in Supplementary Fig. S2 were fabricated by photoresist reflow followed by pattern-transfer from the resist to the silicon surface using inductively coupled plasma reactive ion etching (ICP-RIE) (see Methods for fabrication details). As a result, the design freedom of the curvature of the resulting silicon surface is limited by process-dependent factors such as the photoresist reflow behavior and the subsequent dry etching selectivity and etch transfer characteristics between the photoresist and the silicon. Therefore, the silicon domes in Supplementary Fig. S2b are not one-to-one curvature-matched counterparts to the TPP printed polymer dome structures shown in Fig. S2d.

Collectively, to demonstrate the capability of defining scaffold masks on pre-existing 3D topographies, we successfully applied the SALO process to initially non-planar 3D substrates, including KOH-etched silicon pyramids, convex silicon domes, and dimensionally constrained end-face of an optical fiber.

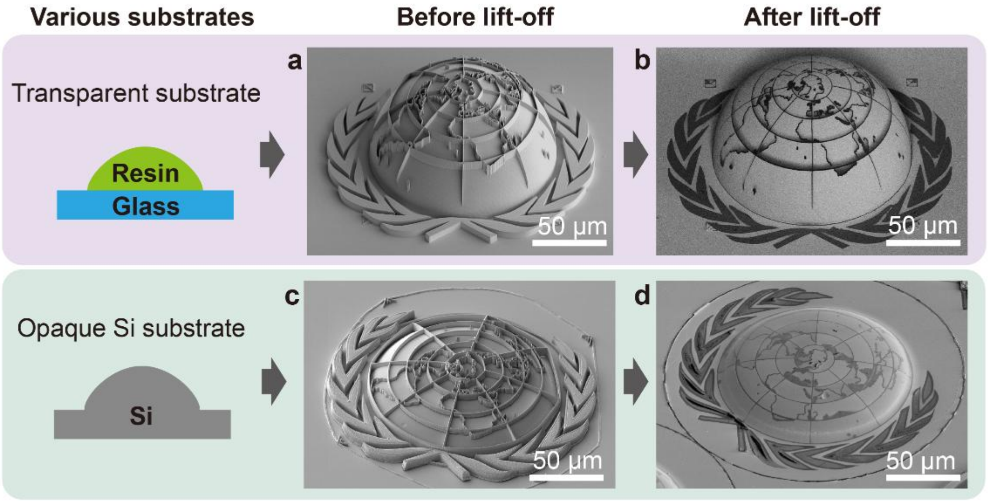

Supplementary Fig. S2. SALO of thin film coatings on curved surfaces a, b | Patterning of the United Nations logo in a 50 nm thick sputtered Ni coating on a curved surface consisting of TPP polymer (resin) on a glass substrate. **a |** Before lift-off. **b |** After lift-off. **c, d |** Patterning of the United Nations logo in a 50 nm thick sputtered Ni coating on a curved silicon surface. **c |** Before lift-off. **d |** After lift-off.

Supplementary Note 3. Alignment accuracy

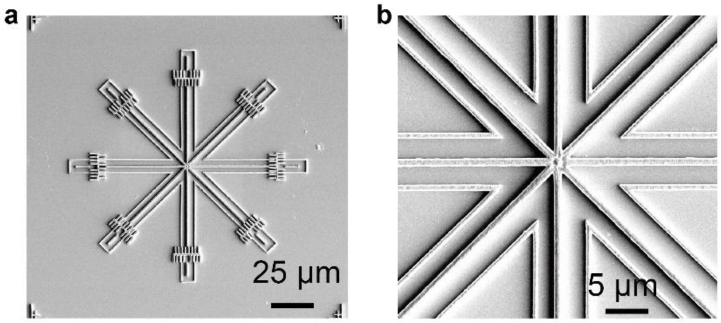

Supplementary Fig. S3. Characterization of alignment accuracy of the TPP printing system. a | A nested star-shaped calibration pattern was used to evaluate lithography overlay alignment errors. **b** | Within a 140 µm × 140 µm printing area, the lateral (x–y) overlay misalignment was found to be less than ±1 µm.

Supplementary Note 4. SALO patterning of coatings deposited by ALD

Here our SALO process offers capability to remove conformal films with spatial selectivity, including on curved and shielded surfaces where etching is impractical. We first test SALO against geometries that typically defeat lift-off with non-vertical masks. To illustrate 2D patterning with non-vertical masks, we implemented SALO on slanted masks and capped (closed) masks. In this experiment, a 20nm ALD Al_2O_3 layer is deposited at 170°C after scaffold printing. As shown in Fig. S4a, stool-like micropillar arrays and inclined microplates (≈60°) are patterned with preserved fidelity, indicating that mask non-verticality does not compromise the process. During design-space screening we observed that a subset of low-aspect-ratio scaffolds did not release, which fall outside the effective lift-off window. Furthermore, we demonstrated process robustness in large-area 3D arrays (Fig. S4b): mm-scale patterns comprising 100 µm × 30 µm disks with sub-micrometre features are reproduced across the array.

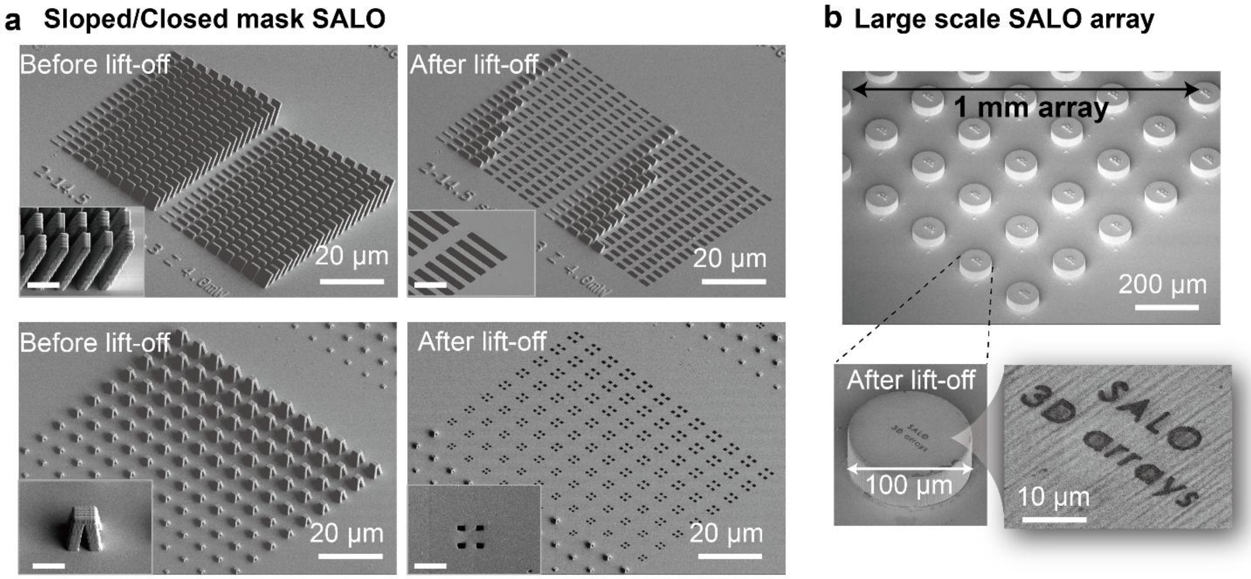

Supplementary Fig. S4. SALO of thin-film coatings on 2D and 3D arrays using an ALD conformal layer. **a** | 2D patterning with non-vertical masks: slanted-mask lift-off (top) and capped-mask lift-off (bottom). Scale bar, 5 µm (insets). **b** | 3D array patterning by SALO yielding sub-µm minimum features across millimetre-scale arrays.

Supplementary Note 5. Thin film integrity at the pattern edges

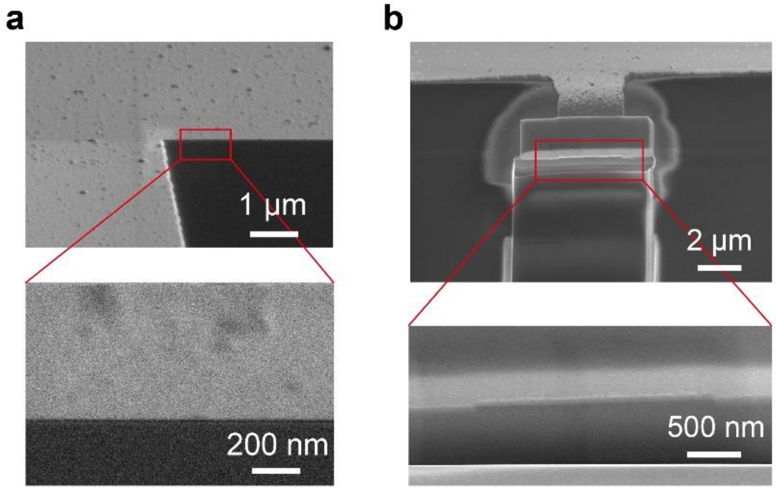

Supplementary Fig. S5. Edge integrity of SALO-patterned ALD films. a | Close-up SEM image of a SALO-patterned 25 nm ALD ZnO film, highlighting the edges of the pattern. No evidence of film delamination, cracking, or film discontinuity is observed. **b** | FIB-milled cross-sectional image of the patterned ZnO film at the edge region, confirming conformal film coverage and an intact film-substrate interface without observable delamination.

Supplementary Note 6. Uniform thin film coverage

For highly recessed or high-aspect-ratio scaffold mask geometries, surface-reaction-limited deposition processes such as ALD avoid the geometric shadowing associated with line-of-sight coating processes, resulting in uniform film thickness across the scaffold profile, as shown in Fig. S6. In contrast, when more directional line-of-sight thin film coating processes are used, such as PVD, the overhangs, sidewall angles and spacings of the scaffold mask become important design parameters for ensuring thin film coverage near the scaffold base. AFM topography and line-profile analysis of a sample corresponding to the thinnest ALD layer (15 nm thick HfO_2) we have patterned by SALO, further confirming that the patterned ALD layer thickness was consistent with the nominal thickness (Fig. S6c).

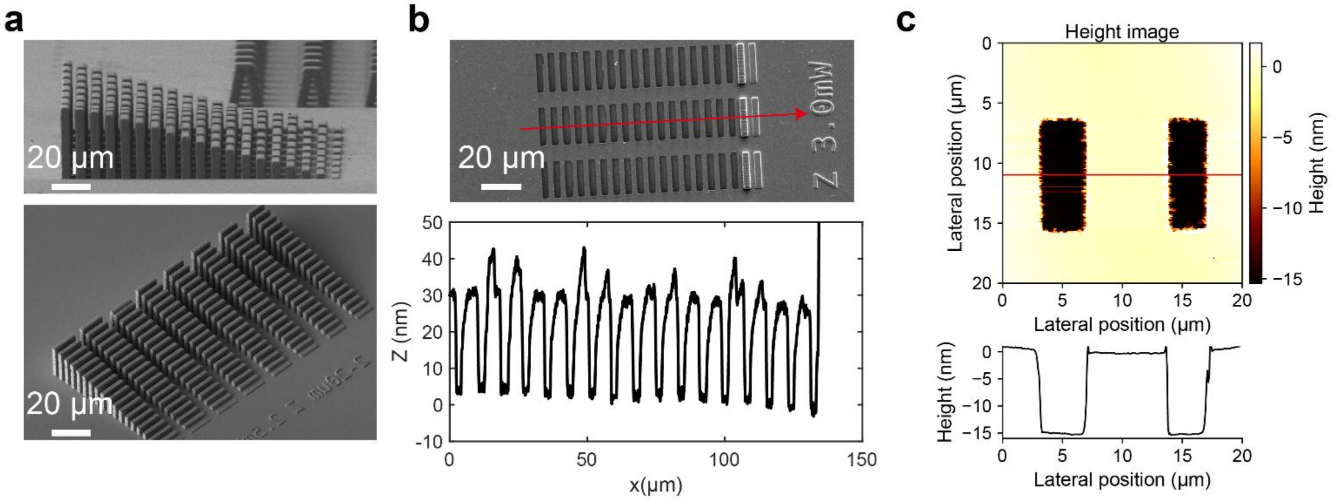

Supplementary Fig. S6 | High-aspect-ratio scaffold geometry and conformal film coverage. a | SEM images of representative high-aspect-ratio scaffolds fabricated by TPP printing. **b** | Top-view SEM of SALO-patterned 25 nm ZnO thin film which was deposited inside the deep trench of the very-high aspect ratio scaffolds. The red arrow indicates the line-scan direction and the corresponding height profile, showing a constant ALD coating thickness of about 25 nm across the gaps that were surrounded by scaffolds with different aspect ratios. The spikes in the line-scan are due to particles at the surface (confirmed by SEM imaging) that were present before the ALD layer was deposited. **c** | AFM thickness measurement of the SALO-patterned 15 nm thick ALD HfO_2 layer, used as a representative sample to verify the actual ALD layer thickness.

Supplementary Note 7. Patterning accuracy of sub-micrometer scale features

Using TPP printed (IP-Dip) scaffolds on a glass substrate and subsequent SALO-patterning of a 20 nm thick sputtered Cr film, we quantified the deviation between the nominal pattern diameter of the scaffold mask and the actual feature size of the resulting circular pattern in the thin film. The results show that circular features with nominal diameters down to 400 nm can be reproducibly defined, with measured standard deviations below 40 nm across the evaluated nominal dimensions (Fig. S7). For pillar diameters smaller than 400 nm, the printed scaffold approaches a dose-defined pillar-like regime where the effective feature size and its standard deviation depend heavily on primarily by voxel size and exposure conditions rather than the CAD design.

We also show that reliable shape differentiation is maintained for feature sizes of approximately 1 μm for the evaluated scaffold geometries, whereas at smaller dimensions the voxel geometry and proximity effects increasingly dominate the shape of the printed structure (see circle vs square comparison in Fig. S7a).

Importantly, the ultimate resolution and dimensional fidelity are limited by the specific TPP laser printing system used and by the printing process calibration, since the voxel size and printing accuracy depend on multiple interconnected parameters, such as the exposure dose, system optics, and proximity effects, and these parameters vary across TPP tools and photoresists.

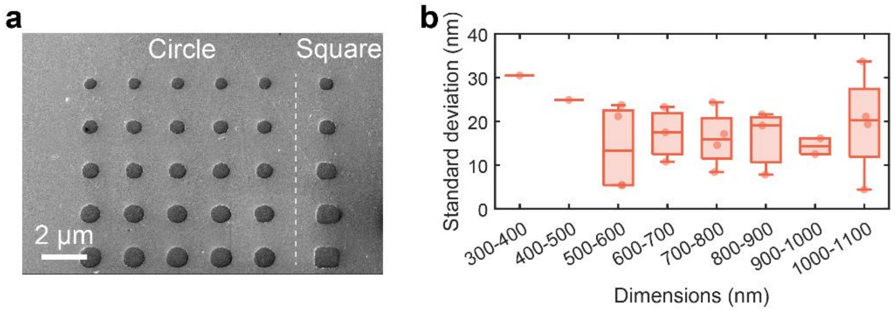

Supplementary Fig. S7 | Dimensional accuracy of patterned features and variability at the sub-micrometer scale. a | SEM image of representative sub-micrometer sized circular and square patterns used for dimensional metrology (scale bar: 2 μm). **b** | Standard deviation of the measured diameter of circular patterns as a function of the nominal diameters, showing dimensional variability across the evaluated range of pattern diameters. At least five different pattern samples were measured for each nominal diameter to calculate the standard deviation.

Supplementary Note 8. Edge roughness of patterns

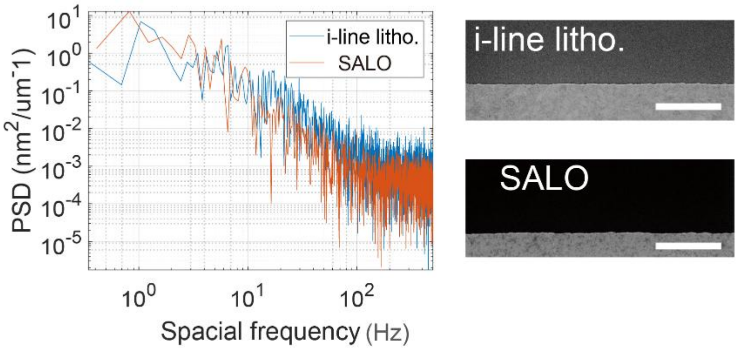

Supplementary Fig. S8. Comparison of line edge roughness between patterns realized by i-line lithography and SALO. | i-line lithography: 15 nm of ALD HfO_2 was etched by RIE (PlasmaPro 100 Cobra) for 55 s. SALO (100 nm sputtered Ag film patterned on SiO_2 surface), lithography details are described in Methods (lithography part). SEM images show top views of line patterns on SiO_2 surface, used for power spectral density (PSD) comparison. Scale bar 100 nm.

Supplementary Note 9. Lift-off statistics of circular pillar scaffolds

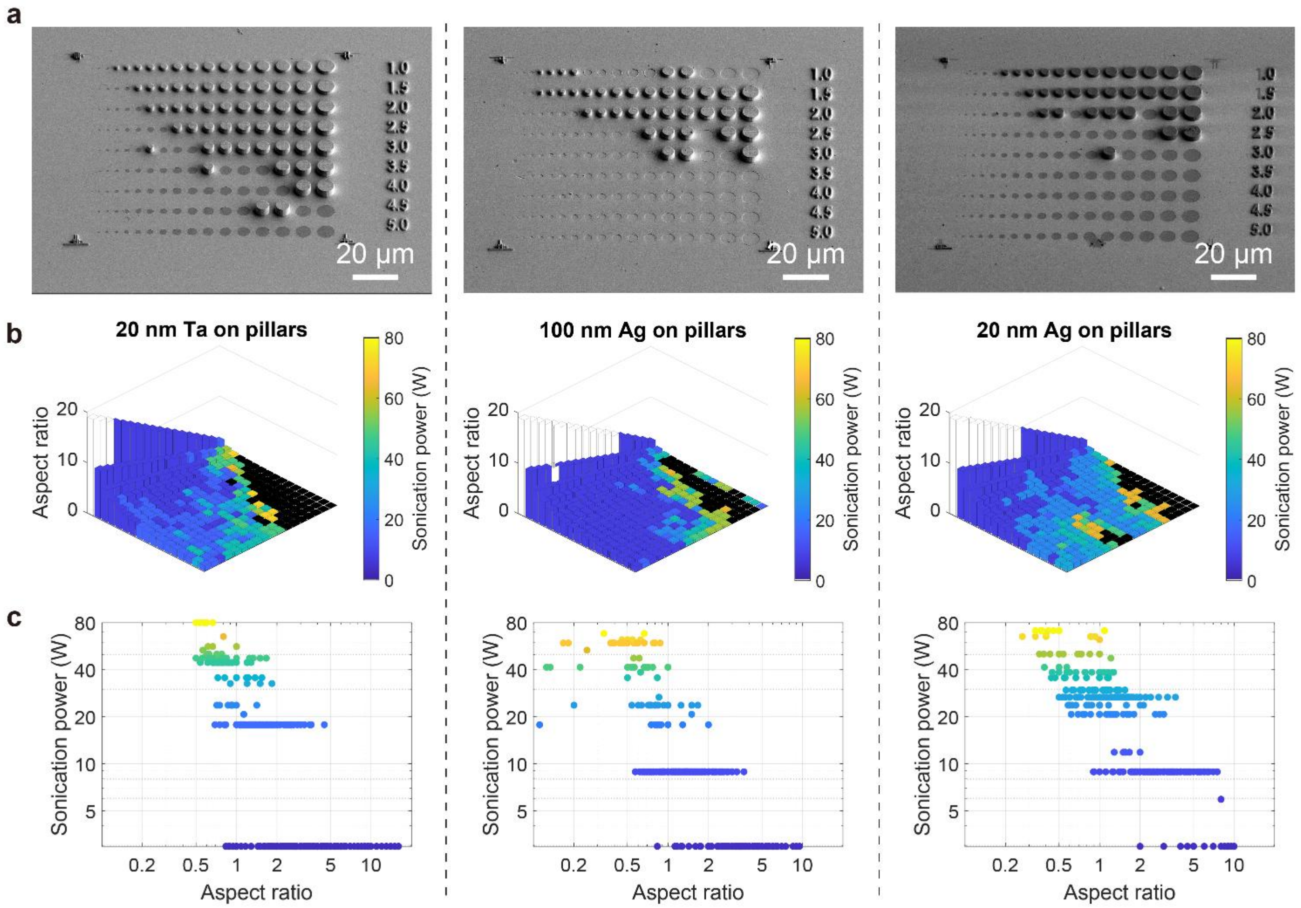

Supplementary Fig. S9. SALO statistics of circular pillar scaffolds. a | SEM image of different evaluated scaffolds after SALO. **b** | Lift-off statistics concerning aspect ratio. **c** | Sonication power needed for lift-off as a function of scaffold aspect ratio.

Supplementary Note 10. Modelling and simulations

To examine whether the proposed microjet-driven mechanism for lift-off is consistent with the observed lift-off behavior of the scaffold during sonication, we developed a semi-quantitative mechanical model evaluating the hydrodynamic forces exerted by ultrasonic cavitation. In ultrasonic surface cleaning, the violent collapse of asymmetric cavitation bubbles near a solid surface generate high-velocity microjets, which are widely recognized as the primary hydrodynamic mechanism responsible for dislodging surface particles and structures[4,5]. In our experiments, we observed a strong and systematic aspect ratio (AR)-dependence of the scaffold micro-pillars that are detached during sonication: High-AR pillars consistently failed at lower sonication power than lower-AR pillars. This selective behavior is consistent with a mechanism involving lateral bending moments, which scale with AR^2, rather than compressive loading from isotropic shock waves. Therefore, based on our analysis, as outlined below, using established ultrasonic cleaning models and our experimental observations, we believe that microjet-induced lateral impact is likely the dominant physical driver for the observed structural failure of the scaffolds during sonication.

10.1. Validation of Microjet Impingement Model

For ultrasonic cavitation with frequencies in the tens of kilohertz regime, reported microjet velocities are on the order of 100 m/s in water[5,6], and the corresponding impact duration is therefore expected to be in the sub-microsecond to microsecond range based on the characteristic jet length. In isopropanol (IPA), the jet velocity is expected to be lower than in water because the higher vapor pressure can modify cavitation dynamics. The microsecond-scale load is within a timescale at which the dynamic bending response mode of a pillar-shaped scaffold mask becomes relevant. Based on the pillar dimensions of the scaffold used in this study, the natural oscillation period of the pillars is estimated to be of the order of tens to hundreds of nanoseconds using Euler-Bernoulli cantilever beam theory, comparable to the duration of the water hammer phase, suggesting that dynamic effects may partially contribute to the peak stress. Specifically, when the impact duration closely matches the structure's natural period, it triggers dynamic amplification, resulting in a transient peak stress that is significantly higher than the stress caused by an equivalent static load.

Our transient simulations (Supplementary Fig. S10a) show a clear dynamic response of the pillars when applying a step pressure load, where the peak stress can be amplified by a factor of maximum 2 (no damping in the system) due to inertial effects, compared to the static case. To assess the transient structural response of the pillar under impulsive microjet loading, we performed time-dependent simulations using Rayleigh damping with the stiffness-proportional coefficient beta set to 5×10^{-9}, and 1×10^{-9} s, representing overdamped and underdamped conditions, respectively. Since the dynamic stress amplification factor under sudden step loading is limited to a maximum of 2 for undamped systems, and decreases with increasing damping, the static pressure simulations adopted here represent a conservative lower bound.

To evaluate the structural integrity of the micro-pillars under the influence of cavitation-induced microjets, the impact of a microjet is modeled as a sustained drag force. According to the classification by Liu et al[7], the first phase of the jet impact is characterized by the water hammer pressure:

$$p_{hammer} \approx \rho c v \tag{1}$$

where $\rho \approx 786\mathrm{kg}\cdot\mathrm{m}^{-2},\ c = 1170 m \cdot s^{-1}$ for IPA, and v is the microjet velocity in dependence of the

sonication power.

To approximate the hydrodynamic loading relevant to the SALO process, we selected a microjet velocity within the lower end of values reported for near-wall cavitation-driven flows. Prior studies have reported jet-impact or jet-associated local flow velocities ranging from several meters per second to several tens of meters per second in controlled cavitation systems, including about 5~80 m/s [8] in near-wall bubble studies and local flow velocities on the order of 10 m/s [9] near the tip of a laser-generated microjet. Because the local microjet velocity in a laboratory ultrasonic bath is highly transient and difficult to determine directly, we used 10 m/s as a conservative order-of-magnitude estimate rather than as a direct measurement of the flow field in our system. The scaffold lift-off process can be modeled as a two-stage physical process: First, upon the initial impingement of the microjet, a transient water hammer pressure ($P_{WH} = \rho c v \approx 9.2$ MPa for IPA) delivers an intense, impulsive shock. This causes instantaneous high-stress concentrations at the edges of the base of the pillar, crack formation by effectively exceeding the interfacial adhesion strength between the pillar and the substrate surface, and dislodging the IP-Dip pillar scaffolds at their base. Second, the transient shock dissipates into a steady radial liquid flow with a much lower stagnation pressure ($P_{dyn} = 0.5\rho v^2 \approx$ 39 kPa). Rather than inducing further structural fracture, this residual hydrodynamic flow primarily serves to flush away the already detached scaffolds, clearing them from the underlying thin films and completing the lift-off process[10].

10.2. Corroboration with Experimental Scaling Laws

The most compelling evidence for the hypothesis that the lift-off threshold of the scaffold pillars is governed by beam-bending mechanics is its strong dependence on the aspect ratio (AR) of the pillars, which is consistent with beam theory mechanics. For a cantilever beam under a distributed pressure p (representing the microjet impact), the maximum stress at the pillar base (at the interface between the pillar and the substrate surface) scales as:

$$\sigma_{max} = \frac{M \cdot c}{I} = 3p \cdot \frac{h^2}{w^2} \tag{2}$$

where M = (p * w * h) * (h/2) is the bending moment; c = w/2 is the distance to the neutral axis; I = $w^4/12$ is the area moment of inertia; h is the pillar height and w is the pillar width. However, since the interfacial failure stress between IP-Dip photoresist and glass, as measured by Izard et al[2], represents the spatial average over the entire cross-section of the area of the pillar base, and the bending stress is linearly distributed across the neutral axis of the pillar, the cross-sectionally averaged stress magnitude σ_{ave} is theoretically predicted to be 1/2 of the maximum stress.

$$\sigma_{ave} = 1.5p \cdot AR^2 \tag{3}$$

This is consistent with our FEM simulation results shown in Supplementary Fig. S10b, validating our simulation framework. The deviation between the FEM results and the analytical Euler-Bernoulli beam theory at low AR is primarily due to the breakdown of the slender beam assumptions. For short pillars (low AR), transverse shear deformation, which is neglected in Euler-Bernoulli theory, becomes significant and comparable to bending deformation.

Given a constant adhesion stress threshold σ_{adh}, the pressure by the microjets needed for scaffold detachment is:

$$p \propto AR^{-2} \tag{4}$$

Assuming the effective acoustic pressure increases approximately with the square root of the selected bath power setting ($p_{applied} \propto \sqrt{P}$), the power threshold for lift-off of the scaffold pillars should theoretically

follow a power law of

$$P_{applied} \propto AR^{-4} \quad (5)$$

To validate the scaffold lift-off mode, we compared our calculated average stress at the pillar base with the detachment thresholds reported by Izard et al[2], where the required detaching shear stress σ_{adh} for a scaffold with a footprint of 100 μm^2 were 12 MPa, which was then used as a detachment threshold in our FEM simulation (Fig. S10b, red line). Because this value was reported for a specific scaffold geometry and interfacial system, it should be regarded here as a semi-quantitative reference rather than a universal detachment criterion. A purely analytical derivation of the power-AR relationship remains challenging. However, our experimental data (Fig. 4) reveals a strong linear correlation on a logarithmic scale (log Sonication Power vs. log AR), demonstrating that the structural stability of the scaffold pillars drops sharply at higher aspect ratios. This trend is consistent with the theoretical AR^{-4} dependence of bending stress in cantilever structures. To contextualize our experimental failure observations, we performed a parametric FEM sweep over the full scaffold array geometry used in the sample shown in Fig. 4, varying both pillar aspect ratio and sonication power ($\propto p^2$) (Fig. S10c).The resulting von Mises stress heatmap (Fig. S10d) shows that, when 12 MPa (dashed line in Fig. S10d) is taken as a reference interfacial detachment threshold, the experimentally observed failure boundary corresponds closely in order of magnitude to the cross-sectionally averaged stress at the pillar base. This analysis supports that the pillar detachment observed in the sample in Fig. 4 is governed by interfacial stress failure between the pillar and the substrate, driven by flexural bending under directed lateral loading.

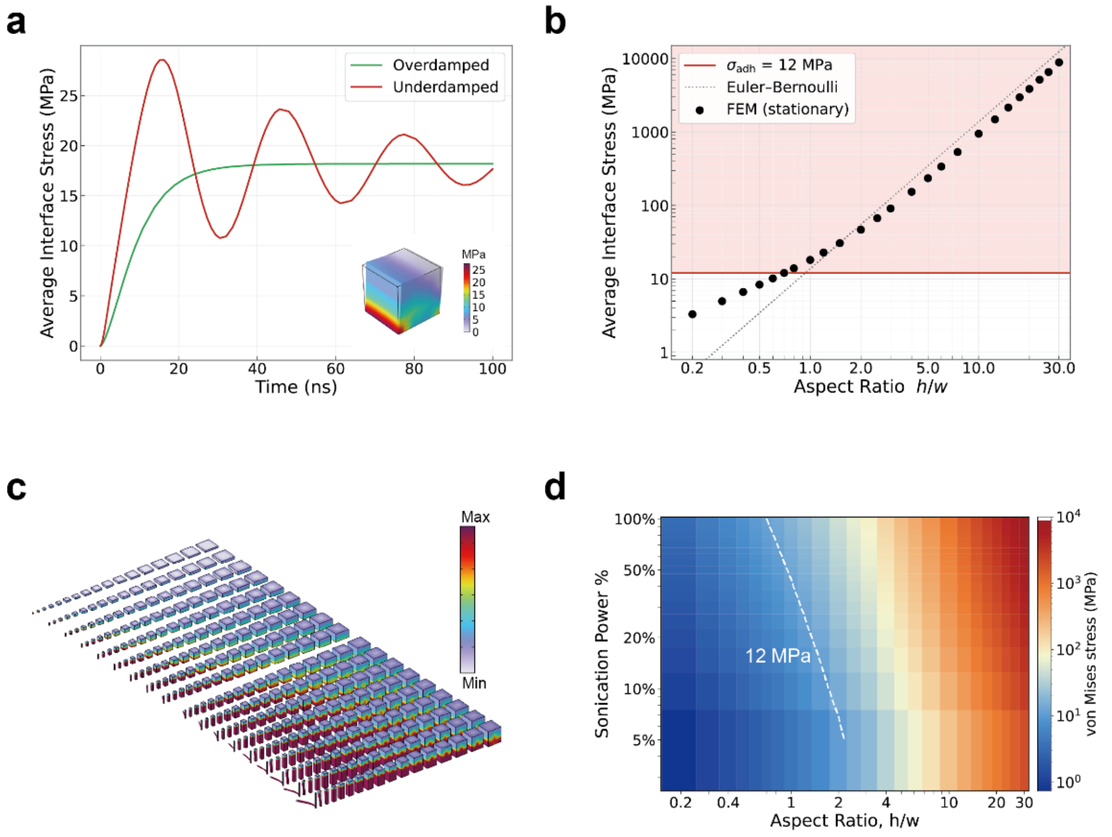

Supplementary Fig. S10. COMSOL simulation of resulting interface stress in dependence of sonication power and aspect ratio (AR) of scaffold pillars. a | Transient interfacial stress response of a single scaffold pillar (AR = 1) under a step pressure load of 9.2 MPa, simulated under two Rayleigh damping conditions (underdamped: $\beta = 1\times10^{-9}$ s; overdamped: $\beta = 5\times10^{-9}$ s). The peak dynamic stress is 1~2× the static value, depending on the damping condition, with the underdamped case approaching the theoretical dynamic amplification factor of 2. Inset: Representative von Mises stress distribution in the pillar. **b |** Stationary FEM simulations of average interfacial von Mises stress as a function of pillar aspect ratio (AR = h/w), with a uniform lateral step pressure load of 9.2 MPa applied to one sidewall. FEM results (filled circles) show good agreement with the Euler–Bernoulli analytical prediction (dotted line). The red horizontal line marks the experimentally measured adhesion strength (by Izard et al[2]) at the interface between the pillars and the substrate surface; pillars with aspect ratios above this threshold (shaded region) are predicted to detach. **c |** 3D rendering of the full scaffold array geometry (identical to the pillar arrays shown in Fig. 4) used for parametric stress simulations, illustrating the variation in pillar height and stress distribution across the pillar array. **d |** Heatmap of simulated average von Mises stress at the pillar-substrate interface as a function of pillar aspect ratio and sonication power. The dashed white line marks the 12 MPa detachment threshold, in good agreement with the experimentally observed detachment boundaries of the pillars in the sample shown in Fig. 4.

Supplementary Note 11. Large area SALO patterning with shell-type scaffolds

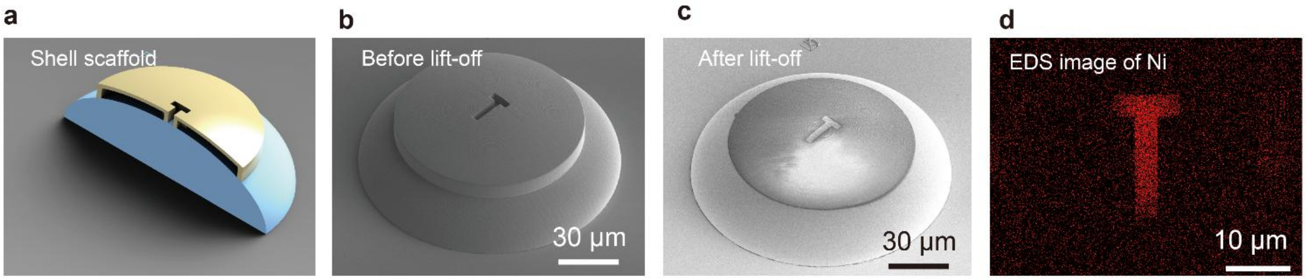

Supplementary Fig. S11. SALO process of large area patterning using shell-type scaffold. a | Schematic cross-sectional view of shell-type scaffold. **b** | SEM image of printed shell-type scaffold before lift-off. **c** | Conformal pattern after lift-off using shell-type scaffold. **d** | EDS image of letter "T" patterned in 20 nm thick Ni layer.

Supplementary Note 12. Effects of storage conditions of shell-type scaffolds on lift-off results

To determine the practical processing window of shell-printed scaffold masks and to assess whether storage before lift-off increases the risk of residual uncrosslinked resin, we carried out a controlled storage study on samples after scaffold printing and prior to the SALO process. Representative SEM images in Fig. S12 show patterned areas after the lift-off process was performed on samples with freshly printed scaffold masks, and for samples in which the scaffold masks were stored for 10 or 20 days under four different conditions, respectively: (1) Ambient laboratory light at room temperature (23 °C) in air (30% RH). (2) Dark at room temperature (23 °C) in air (30% RH). (3) Dark at 3 °C in air (30% RH). (4) Ambient laboratory light at room temperature (23 °C) under vacuum (10% RH). All samples used 10 μm × 10 μm × 10 μm scaffolds printed in shell mode on an ITO-coated glass substrate to pattern a 20 nm thick sputtered Cr layer.

As shown in Fig. S12, under all evaluated conditions, the shell scaffolds successfully lifted off without any measurable degradation of the resulting pattern. However, the presence of polymer residues remaining in the patterned areas (prior location of the scaffolds) was primarily dependent on light exposure during storage of the samples after scaffold printing and before lift-off. While samples based on freshly printed scaffolds (row 1, fresh) exhibited no significant polymer residues after the lift-off process, samples with scaffolds stored under ambient laboratory light for 10 and 20 days (row 1 and row 4, 10 and 20 days) showed minor polymer residues, consistent with the partial curing of the trapped uncrosslinked resin. In contrast, storing the samples in a dark environment (row 2 and row 3) markedly reduced these polymer residues after lift-off of the scaffold. Within the evaluated range, no clear and reproducible effect of temperature or humidity on the amount of residual polymer was observed. These results indicate that light exposure during storage of samples carrying shell-printed scaffolds, prior to lift-off, is the dominant factor promoting residual polymer formation in the lift-off patterned areas. As shown in the rightmost column of Fig. S12, a brief intermediate oxygen plasma clean (RF power 20 W, 50 mTorr, O_2 flow 20 sccm, 2 min) serves as a highly effective and universal rescue step to remove any residual organic traces induced by prolonged curing.

Finally, we found that incidental exposure to direct sunlight of samples with shell-printed scaffold structures must be strictly avoided, as the high-intensity UV component of sunlight can rapidly cure the uncured resin encapsulated by the shell structure, and compromise the subsequent lift-off process.

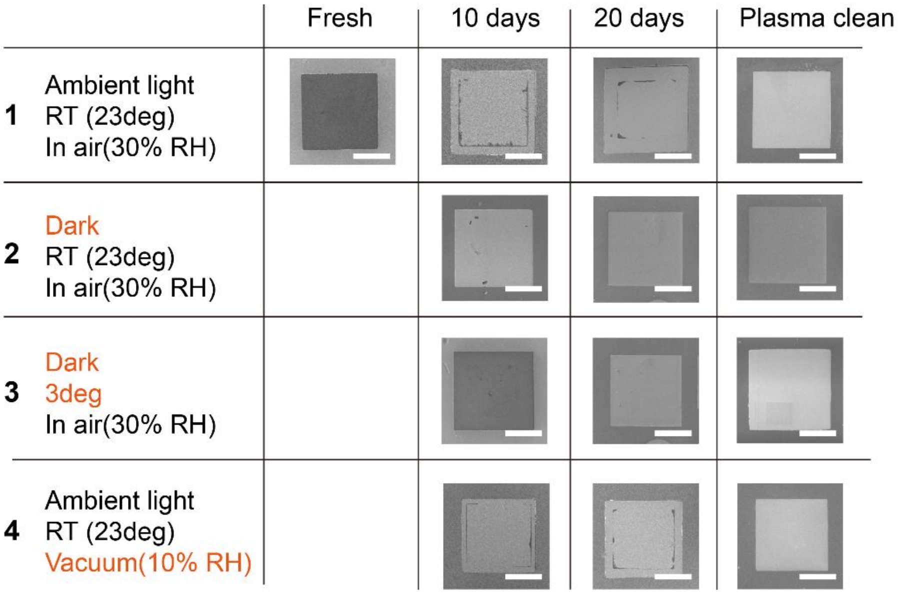

Fig. S12. Storage conditions of shell-printed scaffold masks and its effects on polymer residues from the scaffold structures after the lift-off process. Representative SEM images of SALO-patterned areas after the scaffold lift-off are shown for samples processed (film deposition and lift-off) immediately after scaffold printing ("Fresh") or stored after scaffold printing and before film deposition and lift-off under different environmental conditions. Row **1**: Ambient laboratory light at room temperature (23 °C) in air (30% RH). Row **2**: Dark at room temperature (23 °C) in air (30% RH). Row **3**: Dark at 3 °C in air (30% RH). Row **4**: Ambient laboratory light at room temperature (23 °C) under vacuum (10% RH). Storage condition differences were marked in orange. Columns correspond to samples processed fresh, or after storage for 10 or 20 days, followed by lift-off. The rightmost column ("Plasma clean") shows the corresponding samples after a brief intermediate O_2 plasma treatment. Fresh samples are shown only for condition 1. All samples used ITO-coated glass substrates with shell mode printed scaffolds (10 μm × 10 μm × 10 μm) to pattern 20 nm thick sputtered Cr films. Scale bar 5 μm.

Supplementary Note 13. Surface analysis after the SALO process

To evaluate potential organic contamination following scaffold removal by sonication in an IPA bath, we performed X-ray photoelectron spectroscopy (XPS) measurements immediately after the SALO process and drying, without any additional plasma cleaning (Fig. S13). In these experiments, we used a glass substrate with an Indium Tin Oxide (ITO) surface coating and employed the SALO process to pattern a 20 nm thick sputtered Cr layer. The XPS measurements were carried out in ultrahigh vacuum ($<1\times10^{-8}$ Torr) using a monochromated (hν = 1486.69 eV) Al Kα X-Ray source (Kratos Axis Supra+, Kratos Analytical Ltd, Manchester, UK) with charge neutralization. Binding energies were calibrated to C 1s peak at 285.0 eV. Survey spectra were collected at a power of 75 W, 40 mA emission current, while core level spectra and images were collected at a power of 600 W with varying acquisition time depending on required signal-to-noise.

To evaluate potential polymer residues, XPS analysis was performed on a SALO-patterned region (P1, exposed ITO) and an adjacent unpatterned reference region (P2, remaining Cr-coated surface) on the same substrate. The XPS survey spectra (Fig. S13c) confirms the distinct surface compositions, showing strong In 3d signals in P1 and Cr 3p signals in P2. As shown in Fig. S13d, the C 1s spectral shapes and peak positions on both the patterned and unpatterned surfaces are nearly identical. They predominantly consist of typical adventitious carbon features, dominated by C–C bonds at ~284.8 eV. Crucially, when compared to the reference spectrum of the printed IP-Dip photoresist (sample in Fig. S1c), both P1 and P2 regions lack the distinct, intense oxygen-containing functionalities (such as C–O and C=O bonds) that are characteristic of the polymer[11,12]. These results indicate that no significant polymer residue is detectable by XPS on the tested SALO-patterned surfaces.

It is important to note that these measurements were conducted on 100 µm × 100 µm (P1) areas, which were patterned using a scaffold mask that was TPP printed using the shell printing mode. This area size was selected because reliable chemical analysis requires a targeted surface area that is significantly larger than the nominal beam diameter, which in our XPS system is 15 µm in spectroscopy mode.. Analysis of small, patterned regions would result in signal convolution from the adjacent unpatterned areas. When smaller patterns are realized using the SALO process, the TPP printing mode results in solid scaffolds without enclosed volumes of uncrosslinked resin. Based on this design choice, we expect that these small, patterned areas are at least as clean (and likely cleaner) as the characterized areas (P1) for which large scaffold structures were used that were TPP printed using the shell printing mode, featuring scaffolds containing uncrosslinked resin.

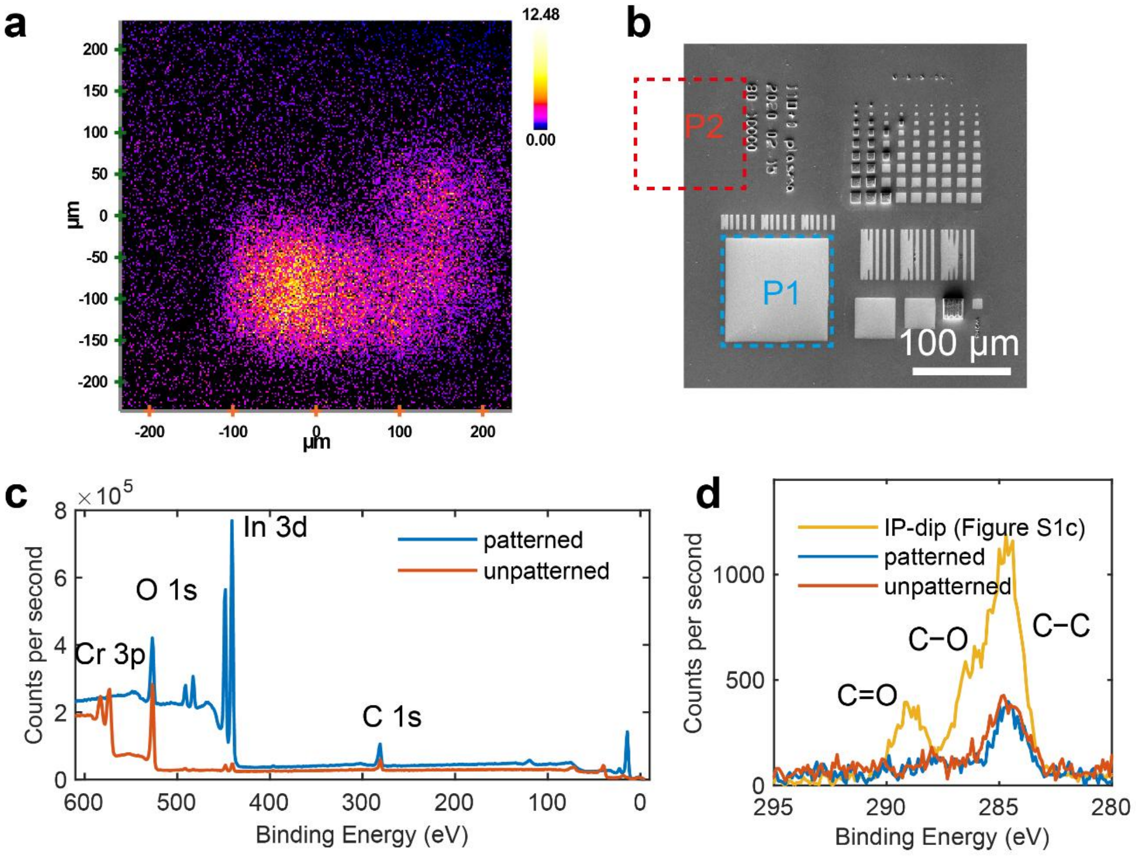

Supplementary Fig. S13. XPS analysis comparing carbon-based surface contamination of patterned vs unpatterned areas on an ITO-coated glass substrate. **a** | XPS surface map used to locate the patterned and unpatterned areas on the substrate, acquired at a fixed binding energy of 441.5 eV. The color scale indicates the photoelectron count rate (cps) in the sample plane. The substrate consists of an ITO-coated glass substrate (Nanoscribe standard substrate for 63x DILL mode) and the SALO patterned layer is 20 nm of sputtered Cr. The Indium signal serves as an indicator of the exposed ITO surface. **b** | SEM image of the XPS sample showing the patterned region and the measurement locations, two representative positions (P1 and P2) were selected for point spectroscopy. **c** | XPS survey (wide-scan) spectra acquired from patterned and unpatterned areas (step size: 1 eV), providing an overview of elemental composition. **d** | High-resolution C 1s spectrum acquired with a 0.1 eV step size to assess carbon-based surface species and residual organics in the two areas, compared with reference sample of the printed IP-Dip photoresist (sample in Fig. S1c). A baseline correction was applied to the spectrum for comparison.

Supplementary Note 14. Finite difference time domain (FDTD) simulations

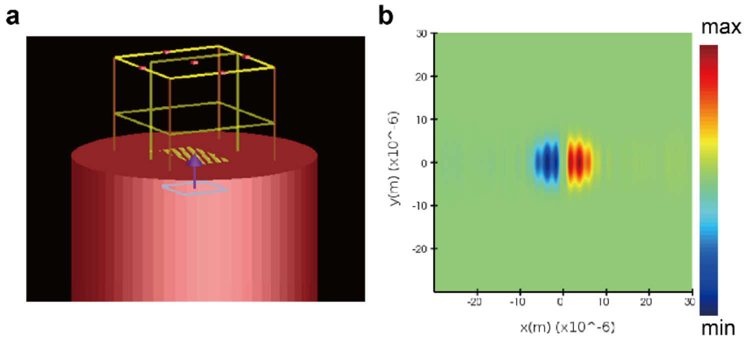

Supplementary Fig. S14. FDTD simulation of the fiber tip grating. a | Setup for finite difference time domain simulation. The mode source was selected to fundamental mode guided in the optical fiber. The top monitor located 40 µm above the fiber tip recorded the E-field magnitude profile across the propagation axis of the fiber. **b** | Simulated direction pattern of the electric field.

Supplementary Note 15. SALO process failure modes

Summary of three representative SALO failure mechanisms and mitigation strategies:

1. Thin Film Delamination
   - Mechanism & Limitation: When depositing metals with weak surface adhesion directly onto certain inert substrates, such as the sputtered Au films shown here, the interfacial adhesion energy between the Au layer and the substrate is often overcome by the internal stress of the thin film and/or the mechanical force applied during the ultrasonic lift-off process. This can result in delamination or tearing of the deposited thin film at the pattern edges (Fig. S15a).
   - Mitigation: This is a fundamental issue relating to thin film adhesion rather than specific to the SALO process. This failure mechanism is readily mitigated by optimized the thin film deposition process, such as the use of an adhesion layer (e.g., 2-5 nm of Ti or Cr) prior to depositing the metal layer (here Au).

2. Laser Exposure Dose Non-uniformity Due to Reflective Surfaces During Scaffold Printing
   - Mechanism & Limitation: Two-photon polymerization (TPP) is sensitive to local light intensities. When printing on reflective substrate surfaces, such as reflective metal films, the focused laser beam interferes with laser light reflected by the substrate surface, creating standing waves and localized intensity hotspots at the substrate interface. This proximity effect can induce unintended over-crosslinking or “scalloping” of the photoresist near the base of the scaffold, which can severely distort and compromise the scaffold geometry (Fig. S15b). This effect limits the applicability of SALO on reflective mirror-like surfaces if no mitigation measures are taken.
   - Mitigation: The optical interferences caused by reflective surfaces can be mitigated by applying a thin anti-reflective coating (ARC)[13,14] prior to TPP printing, or by implementing adaptive exposure strategies that dynamically reduce the laser power precisely at the substrate interface to compensate for the energy of the reflected laser light.

3. Incomplete Development of the Photoresist Near the Base of the Scaffold
   - Mechanism & Limitation: Incomplete photoresist development can result in non-crosslinked photoresist residues trapped near the base of the scaffold (i.e., the interface critical for the SALO process). The resist residues locally prevent coverage of the substrate surface with the deposited thin film, thereby compromising pattern integrity (Fig. S15c). Incomplete resist development can, for example, be caused by restricted transport of the liquid developer (e.g., PGMEA) in scaffolds with high aspect-ratio or dense geometries.
   - Mitigation: This limitation can be overcome by optimizing the resist development protocol. Specifically, extending the development time or introducing gentle agitation of the developer bath can improve removal of unexposed photoresist from dense, high-aspect-ratio regions.

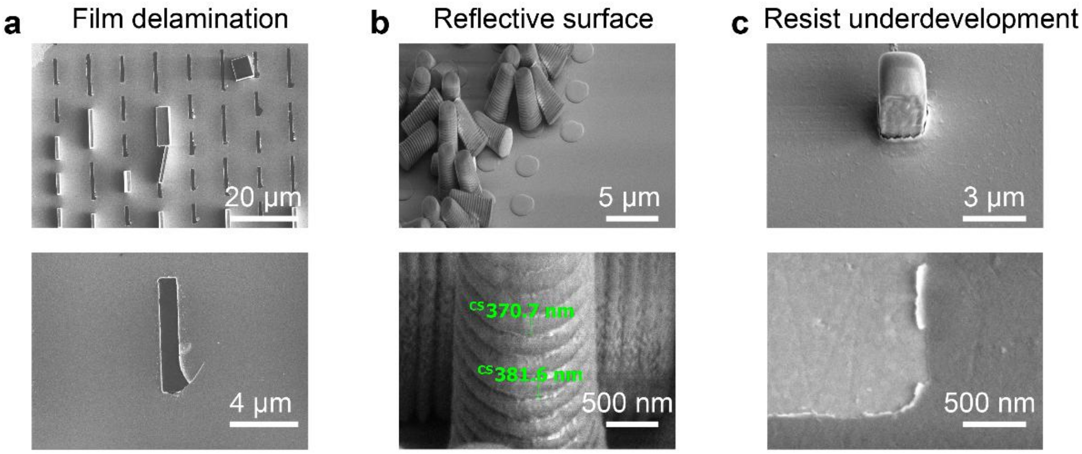

Supplementary Fig. S15 | Possible failure modes and their causes. a | Representative example of observed delamination of a SALO-patterned 25 nm thick sputtered Au film without Ti or Cr adhesion layer. **b** | Effects of exposure dose non-uniformity near the scaffold base during scaffold TPP printing caused by reflections from reflective substrate surface (here sputtered 20 nm Cr), which can compromise the scaffold mask (upper image) by unintended photoresist cross-linking or scalloping (lower image). **c** | Example of incomplete scaffold-mask development leaving resist residues at the scaffold base (here after sputtering 20 nm of Cr). The resist residues can shadow the thin film deposition (upper image) and cause residual flakes at the edges of the patterned film after the SALO process (lower image).

Supplementary Table S1: SALO process results in dependence of scaffold mask aspect ratio (AR) and deposited material and layer thickness of samples shown in Fig. 4 and Supplementary Fig. S9.

**Thin Film and Scaffold Features**	**Collapse AR**	**Lift-off AR**	**Non-releasable AR**
100 nm Ag on circular pillar scaffold	7	0.6-6	<0.6
20 nm Ag on circular pillar scaffold	11	0.5-10	<0.5
20 nm Ta on circular pillar scaffold	17	0.6-15	<0.6
100 nm Ag on square pillar scaffold	9	0.5-8	<0.5
20 nm Ag on square pillar scaffold	17	0.5-16	<0.5
20 nm Ta on square pillar scaffold	16	0.5-15	<0.5
20 nm Ag on wall scaffold	-	>0.4	<0.4

Supplementary Note 16. Scaffold mask printing times and scalability

As detailed in Table S2, the fabrication times for the various scaffold masks demonstrated in our study takes of the order of tens of minutes, reflecting the inherent throughput limitations of conventional serial, single laser beam TPP printing. While patterning scaffold masks on a full 100 mm or 200 mm diameter wafer using our direct write TPP laser tool would be prohibitively slow, the SALO lift-off process scheme is compatible with wafer-scale substrates. To achieve throughputs suitable for wafer-level scaffold-mask printing, recent hardware advances in laser direct writing provide a viable path to substantially increased laser writing speeds. For example, the utilization of digital micromirror devices (DMD) by Somers et al. enable parallel multi-beam laser patterning without compromising diffraction-limited resolution[15]. This parallelization was subsequently massively scaled up by Ouyang et al. using digital holography to operate 2,000 programmable laser foci simultaneously, achieving a printing speed of 2.0×10^6 voxels/s and volumetric processing rates up to 54.0 mm^3/hour.[16] Most recently, a breakthrough employing metalens arrays and spatially adaptive illumination has pushed the printing speed to an unprecedented 1.49×10^8 voxels/s[17]. By adopting such state-of-the-art ultrafast TPP laser writing strategies, the fabrication of SALO scaffold masks is readily scalable to wafer level and industrially relevant speed.

Supplementary Table S2: Representative scaffold mask printing times (non-optimized processes).

Scaffold	Printing Area	Printing Speed	Printing Time
Fig. 1	130 µm × 130 µm	1 000 µm/s	15 min
Fig. 2b	140 µm × 140 µm	1 000 µm/s	18 min
Fig. 3a	100 µm × 600 µm	1 000 µm/s	13 min
Fig. 5c	20 µm × 20 µm	1 000 µm/s	7 min
Fig. 6a	200 µm × 400 µm	10 000 µm/s	10 min
Fig. 6b	30 µm × 60 µm	10 000 µm/s	20 s

Supplementary Reference

1. Jelinek, A., Rossegger, E., Schlögl, S., Kiener, D. & Alfreider, M. Probing local adhesion: A miniaturized multi-photon lithography design demonstrated on silanized vs. untreated surfaces. *Materials & Design* **242**, 112994 (2024).
2. Izard, A. G. *et al.* Enhanced adhesion in two-photon polymerization direct laser writing. *AIP Advances* **10**, 045217 (2020).
3. Han, J. Y., Warshawsky, S. & DeVoe, D. L. In situ photografting during direct laser writing in thermoplastic microchannels. *Sci Rep* **11**, 10980 (2021).
4. Lamminen, M. O., Walker, H. W. & Weavers, L. K. Mechanisms and factors influencing the ultrasonic cleaning of particle-fouled ceramic membranes. *Journal of Membrane Science* **237**, 213–223 (2004).
5. Jia, W. *et al.* Numerical simulation study on the characteristics and mechanism of ultrasonic cavitation cleaning of crude oil sediments. *Ultrason Sonochem* **122**, 107583 (2025).
6. Plesset, M. S. & Chapman, R. B. Collapse of an initially spherical vapour cavity in the neighbourhood of a solid boundary. *J. Fluid Mech.* **47**, 283–290 (1971).
7. Lu, X., Chen, C., Dong, K., Li, Z. & Chen, J. An equivalent method of jet impact loading from collapsing near-wall acoustic bubbles: A preliminary study. *Ultrason Sonochem* **79**, 105760 (2021).
8. Ohl, C.-D. *et al.* Sonoporation from jetting cavitation bubbles. *Biophys J* **91**, 4285–4295 (2006).
9. Sankin, G. N., Yuan, F. & Zhong, P. Pulsating Tandem Microbubble for Localized and Directional Single Cell Membrane Poration. *Phys Rev Lett* **105**, 078101 (2010).
10. Kim, W., Kim, T.-H., Choi, J. & Kim, H.-Y. Mechanism of particle removal by megasonic waves. *Appl. Phys. Lett.* **94**, 081908 (2009).
11. Greczynski, G. & Hultman, L. X-ray photoelectron spectroscopy: Towards reliable binding energy referencing. *Progress in Materials Science* **107**, 100591 (2020).
12. Hantsche, H. High resolution XPS of organic polymers, the scienta ESCA300 database. By G.

Beamson and D. Briggs, Wiley, Chichester 1992, 295 pp., hardcover, £ 65.00, ISBN 0-471-93592-1. *Advanced Materials* **5**, 778–778 (1993).

13. Park, E.-M. *et al.* Investigation of the effects of bottom anti-reflective coating on nanoscale patterns by laser interference lithography. *Thin Solid Films* **519**, 4220–4224 (2011).
14. Astrauskytė, D. *et al.* Anti-Reflective Coatings Produced via Atomic Layer Deposition for Hybrid Polymer 3D Micro-Optics. *Nanomaterials* **13**, (2023).
15. Geng, Q., Wang, D., Chen, P. & Chen, S.-C. Ultrafast multi-focus 3-D nano-fabrication based on two-photon polymerization. *Nat Commun* **10**, 2179 (2019).
16. Ouyang, W. *et al.* Ultrafast 3D nanofabrication via digital holography. *Nat Commun* **14**, 1716 (2023).
17. Gu, S. *et al.* 3D nanolithography with metalens arrays and spatially adaptive illumination. *Nature* **648**, 591–599 (2025).
