# Supplementary material for: Lithographic patterning of conformal thin films on 3D structures using Scaffold-architected Lift-off masks
Source: Nat Commun. 2026 Jul 14;17:6201. doi: 10.1038/s41467-026-75538-z (PMC13369500; doi:10.1038/s41467-026-75538-z)
Supplement: Supplementary file 2 — Transparent Peer Review file [file 41467_2026_75538_MOESM2_ESM.pdf]

nature portfolio

Peer Review File

Lithographic Patterning of Conformal Thin Films on 3D Structures Using Scaffold-Architected Lift-Off Masks

Corresponding Author: Professor Frank Niklaus

This file contains all reviewer reports in order by version, followed by all author rebuttals in order by version.

Version 0:

Reviewer comments:

Reviewer #1

(Remarks to the Author)
This paper reports an approach for patterning conformal thin films on complex 3D surfaces using a 3D-printed sacrificial scaffold as a lift-off mask (SALO). The method demonstrates compatibility with various deposition techniques, multi-scale patterning capability (down to sub-30 nm), and applicability to a wide range of substrate geometries. However, I don't think coating a metal layer on a 3D printed surfaces is novel enough to be published in Nature Communications with the comments bellow.

1. The authors should strengthen the Introduction and Discussion to place SALO in context with the most relevant prior work. Several literatures intersect SALO: sacrificial-structure removal via sonication/solvent, use of printed sacrificial scaffolds for patterning, methods for patterning conformal ALD films (including ALD lift-off and etch routes), and lift-off based on sacrificial 3D masks. The current references list many relevant publications but the manuscript understates overlap and does not make clear what is truly novel. Please explicitly compare SALO with the most closely related prior reports (for example, reports that use 3D printed sacrificial structures + ultrasonic removal, grayscale polymerization + ultrasonic removal, and ALD lift-off strategies). For each, state how SALO differs (e.g., mask design strategy, selectivity by geometry, compatibility with ALD conformal coatings, resolution achieved, multi-material selective release, and process windows). If any prior work already demonstrated selective staged detachment by geometric design or sonic thresholding, this should be acknowledged and compared quantitatively.
2. The central claim is that scaffold aspect ratio (h/w) controls detachment threshold due to hydrodynamic mechanical leverage from cavitation microjets and shock waves. The experiments in Figure 4 show empirical correlations but do not establish a physics-based model nor quantify the forces involved. Please provide a simple mechanical model or scaling argument that links cavitation impulse (or microjet pressure), scaffold geometry (lever arm, bending stiffness of the printed polymer), and adhesion strength to predict a lift-off threshold. This need not be a full computational fluid-structure simulation, but a semi-quantitative mechanics estimate (orders of magnitude) will greatly strengthen the claim of predictability and transferability between labs/equipment. If possible, include finite element or analytical beam bending estimates showing that for typical cavitation impulse the scaffold will fracture/detach at the observed h/w thresholds. This will help readers estimate how to port SALO to different resins, solvents, sonicators and scales.
3. The manuscript states that reduced scaffold-substrate adhesion is important (e.g., 10 nm SiO_2 sputtered on printed polymer). Yet many demonstrations rely on substrate pre-treatments (APTES silanization for resin, SiO_2 for polymer surfaces). The extent to which SALO is general across substrate chemistries is unclear. Please provide XPS/contact-angle data to characterize the surface treatments you used, and show whether a thin adhesion/intermediate layer (e.g., 10 nm SiO_2) is generally required when patterning on polymer substrates or only beneficial in some cases.
4. Cavitation in IPA and removal of polymer scaffolds may leave organic residues on the patterned film that can affect device performance (adhesion, contact resistance, optical loss). Provide surface chemical analysis (XPS, ToF-SIMS or FTIR) on patterned areas after SALO and after recommended rinsing steps, to show absence (or quantify level) of organic contamination. Include data for both small features and large area shell lifts.
5. The shell type scaffolds rely on uncrosslinked interior resin that must remain uncrosslinked until sonication. The authors note that ambient light over days can crosslink these regions and prevent removal. This is an important practical limitation. Please provide quantitative tests: how long can shell type scaffolds be stored in ambient light/dark/packaged conditions before lift off remains successful? Does ambient humidity or temperature affect this window? Is intermediate oxygen plasma always an effective rescue step? These are critical process control data for users.
6. Most significant thing is, the so-called 3D surfaces are printed with the masks, while all the 3D surfaces needed to be

coated with a metal layer is initially 3D surfaces. The situations of this paper is useless unless the authors can printed the masks on an existed 3D structures. Or, many methods can fabricate this masks if the substrate should be fabricated first.

Some other minor comments are as follows：

1. Provide more complete sonication details: bath model and geometry are given, but please add: sample placement (center/edge), use of degassed IPA or not, bath fill volume, whether samples were fixed or floating, actual power calibration method (how the “%” maps to W), and bath temperature during runs. These details are important for reproducibility.
2. However, the manuscript does not provide an in-depth explanation of why mechanical lift-off can overcome the continuous sidewall coverage of ALD films without causing fractures. It is recommended to analyze the interfacial structure at the lift-off edge via cross-sectional TEM or FIB cross-sectioning to verify whether film fracture or a specific delamination mode occurs at the scaffold edges.
3. The manuscript mentions that shell-type scaffolds must be protected from ambient light to prevent unwanted crosslinking. This is a notable practical constraint that should be emphasized in the main text, not only in the Methods.
4. The excellent performance of SALO is demonstrated with several materials (Ag, Ni, Ta, Hf, Au, Al_2O_3, ZnO). Were there any specific material combinations (scaffold/thin-film/substrate) or film stress states that proved challenging for the SALO process, leading to delamination or incomplete lift-off? Are there material properties that would make a system incompatible with SALO?
5. The process requires the substrate with the printed scaffold to be conformally coated. For structures with extreme topographies, such as deep trenches or high-aspect-ratio pillars, could the scaffold itself shadow the deposition, leading to non-uniform coating on the substrate near the scaffold's base? If so, how can this be mitigated in the scaffold design?

Reviewer #2

(Remarks to the Author)
This manuscript introduces SALO, which is a scaffold-architected lift-off technique with the aim to enable high-resolution patterning of conformal thin films (in particular including ALD) on complex 3D surfaces. The approach is indeed original, well demonstrated, supported by strong imaging and well communicated. Crucially, the ability to pattern ALD on steep or even vertical surfaces is particularly worth mentioning. Although really impressive, several aspects need clarification before publication in Nature Communications.

Mechanistic basis of lift-off is a bit underrepresented: The key parameter ($h/w > 1$) is well supported by empirical data. However, physical explanation remains more qualitative. A deeper rationale or discussion of limitations would strengthen the claim of generality. I know this is challenging. However, at least a comment in that direction would be needed.

ALD-patterning claims need more convincing evidence: ALD is a true major highlight, but the examples are kind of limited. I wonder whether the authors have aspect for inclusion such as (i) film integrity after lift-off; (ii) performance on highly challenging geometries or (iii) information on potential failure modes (e.g. delamination or crack formation). That would strengthen that really important case. At least a comment would be needed.

Resolution and fidelity quantification is too shallow: Although the presented SEM images look truly excellent and impressive, the manuscript somehow lacks aspects like statistical relevant information of feature deviation, a discussion of possible error sources and in best case more examples on very small features. Inclusion by either images or at least a short discussion would be really important because resolution is a central claim in that frame.

Proof-of-concept devices: The nanowire and fiber-tip grating are truly impressive and confirm patterning. However, I wonder whether the authors have also information on the device performance, which should be very strong. Hence, if the authors have information in that direction, please include them or have a least a discussion about further plans.

Scalability and throughput: The usual questions for the readership are: (i) what are realistic print times; (ii) is wafer-scale patterning really feasible; or (iii) what about strategies for scaling up the process. A short discussion would broaden the manuscript essentially.

Reviewer #3

(Remarks to the Author)
The work provides significant developments in combining lithography and lift-off tools, pushing them towards the 3D realm at micro- and nano-scales. The experimental study is sufficiently novel and substantial. On the other hand, it is too much hyped and overstressed with some points, which are not accurate and sometimes even contradictory. Thus, it needs a revision.

1. IP-Dip resin was chosen. Why specifically, and what benefits and limitations does it have for the reported study? Would other, let's say pure single acrylate or hybrid materials such as ORMOCERs or ORMOSILs, have advantages due to their different adhesion and resilience? The choice of specific material should be clearly introduced, and a comparison with alternatives could be provided. The simple claim that the IP-Dip has weak adhesion to the substrate is not qualitatively nor quantitatively provided, nor is the influence of the substrate discussed. It appears that adhesion changes could allow for tuning/extending the release during lift-off, correct?
2. Though micro- to nano-scaled patterns on 3D surfaces are claimed, judging from Figure 3, it is contradictory. Namely, the TPP scaffolds reach only 200 nm, whereas high-resolution sub-100 nm features are created by EBL on a flat surface. Thus,

there is no direct evidence of 100 nm on conformal surfaces, as stated.
3. TPP, as an acronym, was introduced twice, but the parameters to replicate the experiment were not provided. Focusing conditions (used objective's NA) are missing, and the mentioned powers in mW do not reveal the actual intensity at the focus, which is essential for inducing the Two-Photon Absorption employed for Two-Photon Polymerization (TPP). Thus, it renders the experiment non-reproducible. It is clear within the community that the intensity is the essential factor determining the non-linear light-matter interaction, enabling 3D structuring, not the average power. This concept was explained in a tutorial paper in the field of Multiphoton 3D lithography. Nat Rev Methods Primers 5, 15 (2025). https://doi.org/10.1038/s43586-025-00386-y . Thus, the exposure conditions should be provided adequately.
4. Figure 4 has lots of information. But besides being visually nice, it's not very easy to extract the specific values for further use. Perhaps a table should be created to summarize the critical parameters, allowing the reader to easily and rapidly find the limits.
5. Figure 5 shows impressive 3D structures used as sacrificial masks, but the produced multi-material structures are on flat or almost flat surfaces. Thus, though the proposed approach is transferable to multi-material deposition, it almost loses its 3D option (at least within the limitations of the study). This must be clearly explained, without overemphasizing that it can be applied to various materials. Since then, it has lost its 3D capacity (not yet proven).
6. ALD has been used in combination with TPP for micro-optical applications, showing its excellent performance as anti-reflective coatings, for instance: Anti-Reflective Coatings Produced via Atomic Layer Deposition for Hybrid Polymer 3D Micro-Optics, Nanomaterials 13(16), 2281 (2023); https://doi.org/10.3390/nano13162281. Authors could consider discussing it and also foreseeing if the SALO technique could be beneficial for optics/photonics. Importantly, in that case, hybrid materials were used, which can withstand higher temperatures and are more mechanically rigid.
7. Could the approach be further expanded by utilizing post-processing solutions, such as thermal annealing (calcination or pyrolysis), to transform the scaffolds into inorganic structures and increase their resilience to etching or lift-off? See a report on that: Laser additive manufacturing of Si/ZrO2 tunable crystalline phase 3D nanostructures. Opto-Electron. Adv. 5, 210077 (2022); https://doi.org/10.29026/oea.2022.210077.
8. Is the approach applicable to all substrates? Or is it limited to some, and what are the criteria for it?
9. 14 mW UV flood exposure sounds unreasonably low. Is it true, or what exactly do these numbers in mW mean?
10. In Supplement's Figure 1, the shape of curved surfaces is different, why? It seems it was supposed to be a parallel comparison, but it its misleading. Also, what is intended to show with the resin mask is that it is possible to combine the removed and non-removed parts by adjusting the AR of the structures? It should be elaborated more on that for easier understanding.

All in all, the manuscript is relatively good, but must be further improved in order to fit the level of the chosen journal.

Version 1:

Reviewer comments:

Reviewer #1

(Remarks to the Author)
I don't think the author well response my question, and the innovation of the present study is not well demonstrated compared to those previous papers.

Reviewer #3

(Remarks to the Author)
The substantially revised version adequately addresses the raised questions.

Reviewer #4

[Editorial Note: Reviewer #4 was recruited to look over the responses to Reviewers #1 and #2.

(Remarks to the Author)
This reviewer read the revised manuscript first, before looking at the previous reviewers' reports, and author's reply/revision. The approach produces a pre-designed 3D Scaffold-Architected Lift-Off (SALO) mask on designated surfaces, then deposits desired materials, and finally lifts the mask. The outcomes are the negatives defined by the mask. The resultant films are conformal to the mask design, with impressive patterns on sloped surfaces with angles up to 90°. The multiscale dimensions from 0.1 mm to tens of nm scale are truly impressive. In my opinion, two aspects of this work warrant a publication on Nature Communication: (a) the outcomes of their lithography, which has high spatial accuracy crossing multidimensions, from nano-, meso-, to macro-size; (b) the science opportunities this report would generate for peers. At the end of the day, the quality of data and thought-provoking work comprises the type of paper that should reach the readers of Nature.

The two reviewers before me covered most of the science and technological issues already. The authors made a comprehensive and good faithed effort to address them point-by-point. I found the replies and revisions to be satisfactory. I recommend the following to improve the broader impacts to benefit Nature's readers.

(1) The concept of "3D surface" is technically mis-used. By stretching, they can be called "supported 3D surfaces" or better yet, they should be referred to as "exterior surfaces of 3D structures."

(2) The thickness of the films, especially among those designed structures, should be accurately measured, e.g., using atomic force microscopy (AFM). It may not be the same as that measured during deposition using QCM.
(3) SiN or Si3N4? I suspect it was the latter.
(4) Discussion and Method cited no references. When past work is mentioned, citing the representative references would be appropriate.
(5) Minor editorial issues, there should be a space between a number and its unit, e.g., 30 nm not 30nm. The manuscript uses them both somewhat randomly, e.g., in Figure 3.

Overall, the work is impressive and thought-provoking. I recommend publication after the above points are addressed.

Version 2:

Reviewer comments:

Reviewer #4

(Remarks to the Author)
The authors addressed my concerns point-by-point. I am satisfied with the revised manuscript, and recommend this work for publication.

Open Access This Peer Review File is licensed under a Creative Commons Attribution 4.0 International License, which permits use, sharing, adaptation, distribution and reproduction in any medium or format, as long as you give appropriate credit to the original author(s) and the source, provide a link to the Creative Commons license, and indicate if changes were made.
In cases where reviewers are anonymous, credit should be given to 'Anonymous Referee' and the source.
The images or other third party material in this Peer Review File are included in the article’s Creative Commons license, unless indicated otherwise in a credit line to the material. If material is not included in the article’s Creative Commons license and your intended use is not permitted by statutory regulation or exceeds the permitted use, you will need to obtain permission directly from the copyright holder.
To view a copy of this license, visit https://creativecommons.org/licenses/by/4.0/

Point-by-Point Response to the Comments of the Reviewers

We thank the referees for the constructive and insightful comments. To address the comments, we have performed additional experiments and analysis. Based on the new results and the referee comments we have substantially improved our revised manuscript. Furthermore, we have extensively edited the manuscript to improve the writing. Our individual detailed responses to the referee comments are provided in the following.

Please note that all page and line numbers referenced in this response letter correspond to the **marked-up version** of the revised manuscript.

Reviewer #1:

Reviewer comment:

> ***This paper reports an approach for patterning conformal thin films on complex 3D surfaces using a 3D-printed sacrificial scaffold as a lift-off mask (SALO). The method demonstrates compatibility with various deposition techniques, multi-scale patterning capability (down to sub-30 nm), and applicability to a wide range of substrate geometries. However, I don't think coating a metal layer on a 3D printed surfaces is novel enough to be published in Nature Communications with the comments below.***

Our response:

We thank the reviewer for this comment. We agree that, when viewed in a simplified form as coating a metal layer on a 3D-printed structure, the approach would not in itself constitute sufficient novelty. However, our approach enables capabilities that go significantly beyond conventional coating and patterning approaches:

Our work introduces a novel patterning strategy that enables multi-scale lift-off of conformal thin films, including atomic layer deposited (ALD) film, on complex 3D topographies and structures, which is not possible using conventional lift-off processes. Our scaffold-architected lift-off (SALO) process employs sacrificial scaffold masks with 3D design features that causes the scaffold to break and release at predetermined release interfaces, thereby enabling deterministic lift-off patterning of conformal thin film coatings. We demonstrate that the SALO strategy can be applied to patterning multiple thin-film materials on different types of complex 3D substrates. Furthermore, SALO enables selective lift-off of hybrid scaffold masks for multi-material patterning with a single mask layer.

To address this comment in the revised Manuscript, we have modified the Introduction section to more clearly highlight the novelty of our approach. With these clarifications and the detailed responses to all reviewer comments provided below, we believe the manuscript is now well suited for Nature Communications.

<u>Revised Manuscript, Page 2, Line 13</u>:

"To address these limitations, here we report a patterning strategy that enables multi-scale lift-off of conformal thin films, including atomic layer deposited (ALD) films, on complex 3D topographies and structures, which is not possible using conventional lift-off processes. We demonstrate the capabilities of our approach by lift-off patterning of diverse thin films on a range of 3D surfaces, including silicon pyramids with steep 54.7° sidewalls, dome-shaped silicon structures, glass, and TPP-printed 3D polymer structures. Our approach uses TPP printing of sacrificial scaffolds as a lift-off mask, combined with a mechanically driven lift-off process using sonication in an ultrasonic bath. By purposefully designing 3D features in the scaffold mask that promote its mechanical detachment during lift-off, selective scaffold removal is achieved via controlled sonication,

thereby enabling clean, damage-free patterning of conformal thin film coatings on complex 3D topographies and structures. We term this method Scaffold-Architected Lift-Off (SALO).”

1. ***The authors should strengthen the Introduction and Discussion to place SALO in context with the most relevant prior work. Several literatures intersect SALO: sacrificial-structure removal via sonication/solvent, use of printed sacrificial scaffolds for patterning, methods for patterning conformal ALD films (including ALD lift-off and etch routes), and lift-off based on sacrificial 3D masks. The current references list many relevant publications but the Manuscript understates overlap and does not make clear what is truly novel. Please explicitly compare SALO with the most closely related prior reports (for example, reports that use 3D printed sacrificial structures + ultrasonic removal, grayscale polymerization + ultrasonic removal, and ALD lift-off strategies). For each, state how SALO differs (e.g., mask design strategy, selectivity by geometry, compatibility with ALD conformal coatings, resolution achieved, multi-material selective release, and process windows). If any prior work already demonstrated selective staged detachment by geometric design or sonic thresholding, this should be acknowledged and compared quantitatively.***

Our response:

We thank the reviewer for this comment and the opportunity to strengthen the Introduction and Discussion parts. In response to this comment, we have expanded our literature review and added four new references (Refs. 37, 38, 39, 40, and 41 in the revised manuscript), discussing other sonication-based release methods and established ALD patterning strategies. We have expanded the comparison with the most relevant prior methods in the main text and clarified where SALO differs in release mechanism, mask design, and compatibility with conformal coatings. We have expanded the literature discussion to better reflect the relevant overlap and distinctions. If this reviewer has been thinking of specific additional prior work that we may have missed, we would be happy to learn about these and include them in Introduction part of our manuscript.

To address this comment in the revised Manuscript, we have updated the respective parts of the Introduction section:

<u>Revised Manuscript, Page 2, Line 21</u>:

“Prior studies have shown that ultrasonic agitation can assist the removal or detachment of TPP-printed microstructures, such as helical microswimmers from substrates.[1] Ultrasonic agitation has also been used to remove overhang supports of TPP-printed structures[2], and to clean off arrays of printed polymer pillars from silicon surfaces[3]. However, in these reports, sonication is used only to separate or clean off polymer structures from substrate surfaces. In contrast, our SALO process utilizes TPP-printed scaffold masks with 3D design features that cause the scaffold to break and releases at predetermined release interfaces when exposed to sonication. This approach enables high-resolution lift-off patterning of conformal thin films, including ALD coatings. While ALD is well suited for coating complex 3D surfaces because of the extremely uniform and pinhole-free surface coverage of ALD processes, the same coating conformality also closes the sidewall pathway of conventional lift-off resist masks that is critically important in solvent-based lift-off processes. One report has addressed this problem by employing a conventional lift-off mask and manually scratching openings in the deposited ALD film to provide solvent access to the underlying lift-off resist[4], which however is not a scalable process. Another report has proposed the use of inkjet-printed growth-inhibiting surface layers that result in area-selective ALD and allow subsequent lift-off[5], however this approach suffers from limited resolution in the micrometer range and is specific to the used ALD process.”

2. The central claim is that scaffold aspect ratio (h/w) controls detachment threshold due to hydrodynamic

mechanical leverage from cavitation microjets and shock waves. The experiments in Figure 4 show empirical correlations but do not establish a physics-based model nor quantify the forces involved. Please provide a simple mechanical model or scaling argument that links cavitation impulse (or microjet pressure), scaffold geometry (lever arm, bending stiffness of the printed polymer), and adhesion strength to predict a lift-off threshold. This need not be a full computational fluid-structure simulation, but a semi-quantitative mechanics estimate (orders of magnitude) will greatly strengthen the claim of predictability and transferability between labs/equipment. If possible, include finite element or analytical beam bending estimates showing that for typical cavitation impulse the scaffold will fracture/detach at the observed h/w thresholds. This will help readers estimate how to port SALO to different resins, solvents, sonicators and scales.

Our response:

We thank the reviewer for this constructive comment. To elucidate the physical mechanism behind the pillar detachment, we evaluated the hydrodynamic forces at play and found that cavitation microjet impingement provides a plausible dominant contribution to the observed lift-off behavior. We introduced a semi-quantitative mechanical model alongside new transient finite element simulation. By estimating the equivalent order-of-magnitude loading of the scaffold pillars from a microjet impact, our simple beam-based analysis suggests that the resulting bending stress scales quadratically with the aspect ratio of the pillar, consistent with our experimental results. This analysis aligns with our experimental observation that high-aspect-ratio pillars fail first.

To address this comment in the revised Manuscript and Supplementary Information, we have added the new simulation results, analysis and discussions.

<u>Revised Manuscript, Page 8, Line 38</u>:

"The localized structural damage and eventual lift-off of the pillars are most consistently explained by the bending moments induced by cavitation microjet impingement (Supplementary Figure S10 and section 11.1) This mechanical fracture mode is further supported by semi-quantitative finite element simulations, which align closely with the experimentally observed damage patterns (Supplementary section 11.2)."

<u>Revised Manuscript, Page 15, Line 9</u>:

"**Finite element simulations**

Stationary finite element simulations were performed using COMSOL Multiphysics 6.4 (COMSOL AB, Sweden). Each scaffold pillar was modeled as a cantilever beam with a square cross-section and a fixed footprint at its base of 5 × 5 μm^2, and varying the pillar height to systematically sweep its aspect ratio (AR = h/w). The pillar material was assigned the linear elastic properties of cured IP-Dip resin, with a Young's modulus of 2.9 GPa and a Poisson's ratio of 0.38, as specified by the manufacturer. A uniform pressure load was applied to one lateral face of the pillar to represent the equivalent hydrodynamic loading from cavitation microjet impingement. The base of the pillar was assigned a fixed boundary condition to represent the rigid interface between the pillar and the substrate. The von Mises stress was spatially averaged over the entire basal cross-section and used as the metric for evaluating interface detachment. A free tetrahedral mesh with a maximum element size of 1 µm was used throughout the pillar volume to ensure sufficient spatial resolution of the simulations, especially at the interface region between the pillar and the substrate."

<u>Revised Supplementary Information, Page 12, Line 1</u>:

"**Modelling and simulations**

To examine whether the proposed microjet-driven mechanism for lift-off is consistent with the observed lift-off behavior of the scaffold during sonication, we developed a semi-quantitative mechanical model evaluating

the hydrodynamic forces exerted by ultrasonic cavitation. In ultrasonic surface cleaning, the violent collapse of asymmetric cavitation bubbles near a solid surface generate high-velocity microjets, which are widely recognized as the primary hydrodynamic mechanism responsible for dislodging surface particles and structures[6,7]. In our experiments, we observed a strong and systematic aspect ratio (AR)-dependence of the scaffold micro-pillars that are detached during sonication: High-AR pillars consistently failed at lower sonication power than lower-AR pillars. This selective behavior is consistent with a mechanism involving lateral bending moments, which scale with AR^2, rather than compressive loading from isotropic shock waves. Therefore, based on our analysis, as outlined below, using established ultrasonic cleaning models and our experimental observations, we believe that microjet-induced lateral impact is likely the dominant physical driver for the observed structural failure of the scaffolds during sonication.

11.1. Validation of Microjet Impingement Model

For ultrasonic cavitation with frequencies in the tens of kilohertz regime, reported microjet velocities are on the order of 100 m/s in water[7,8], and the corresponding impact duration is therefore expected to be in the sub-microsecond to microsecond range based on the characteristic jet length. In isopropanol (IPA), the jet velocity is expected to be lower than in water because the higher vapor pressure can modify cavitation dynamics. The microsecond-scale load is within a timescale at which the dynamic bending response mode of a pillar-shaped scaffold mask becomes relevant. Based on the pillar dimensions of the scaffold used in this study, the natural oscillation period of the pillars is estimated to be of the order of tens to hundreds of nanoseconds using Euler-Bernoulli cantilever beam theory, comparable to the duration of the water hammer phase, suggesting that dynamic effects may partially contribute to the peak stress. Specifically, when the impact duration closely matches the structure's natural period, it triggers dynamic amplification, resulting in a transient peak stress that is significantly higher than the stress caused by an equivalent static load.

Our transient simulations (Supplementary Figure S10a) show a clear dynamic response of the pillars when applying a step pressure load, where the peak stress can be amplified by a factor of maximum 2 (no damping in the system) due to inertial effects, compared to the static case. To assess the transient structural response of the pillar under impulsive microjet loading, we performed time-dependent simulations using Rayleigh damping with the stiffness-proportional coefficient beta set to 5×10^{-9}, and 1×10^{-9} s, representing overdamped and underdamped conditions, respectively. Since the dynamic stress amplification factor under sudden step loading is limited to a maximum of 2 for undamped systems, and decreases with increasing damping, the static pressure simulations adopted here represent a conservative lower bound.

To evaluate the structural integrity of the micro-pillars under the influence of cavitation-induced microjets, the impact of a microjet is modeled as a sustained drag force. According to the classification by Liu et al[9], the first phase of the jet impact is characterized by the water hammer pressure:

$$p_{hammer} \approx \rho c v$$

where $\rho \approx 786 \mathrm{kg} \cdot \mathrm{m}^{-2}$, $c = 1170 m \cdot s^{-1}$ for IPA, and v is the microjet velocity in dependence of the sonication power.

To approximate the hydrodynamic loading relevant to the SALO process, we selected a microjet velocity within the lower end of values reported for near-wall cavitation-driven flows. Prior studies have reported jet-impact or jet-associated local flow velocities ranging from several meters per second to several tens of meters per second in controlled cavitation systems, including about 5~80 m/s [10] in near-wall bubble studies and local flow velocities on the order of 10 m/s [11] near the tip of a laser-generated microjet. Because the local microjet velocity in a laboratory ultrasonic bath is highly transient and difficult to determine directly, we used 10 m/s as a conservative order-of-magnitude estimate rather than as a direct measurement of the flow field in our system. The scaffold lift-off process can be modeled as a two-stage physical process: First, upon the initial impingement of the microjet, a transient water hammer pressure ($P_{WH} = \rho c v \approx 9.2$ MPa for IPA) delivers an

intense, impulsive shock. This causes instantaneous high-stress concentrations at the edges of the base of the pillar, crack formation by effectively exceeding the interfacial adhesion strength between the pillar and the substrate surface, and dislodging the IP-Dip pillar scaffolds at their base. Second, the transient shock dissipates into a steady radial liquid flow with a much lower stagnation pressure ($P_{dyn} = 0.5\rho v^2 \approx 39$ kPa). Rather than inducing further structural fracture, this residual hydrodynamic flow primarily serves to flush away the already detached scaffolds, clearing them from the underlying thin films and completing the lift-off process[12].

11.2. Corroboration with Experimental Scaling Laws

The most compelling evidence for the hypothesis that the lift-off threshold of the scaffold pillars is governed by beam-bending mechanics is its strong dependence on the aspect ratio (AR) of the pillars, which is consistent with beam theory mechanics. For a cantilever beam under a distributed pressure p (representing the microjet impact), the maximum stress at the pillar base (at the interface between the pillar and the substrate surface) scales as:

$$\sigma_{max} = \frac{M \cdot c}{I} = 3p \cdot \frac{h^2}{w^2}$$

where M = (p * w * h) * (h/2) is the bending moment; c = w/2 is the distance to the neutral axis; I = $w^4/12$ is the area moment of inertia; h is the pillar height and w is the pillar width. However, since the interfacial failure stress between IP-Dip photoresist and glass, as measured by Izard et al[13], represents the spatial average over the entire cross-section of the area of the pillar base, and the bending stress is linearly distributed across the neutral axis of the pillar, the cross-sectionally averaged stress magnitude σ_{ave} is theoretically predicted to be 1/2 of the maximum stress.

$$\sigma_{ave} = 1.5p \cdot AR^2$$

This is consistent with our FEM simulation results shown in Supplementary Figure S10b, validating our simulation framework. The deviation between the FEM results and the analytical Euler-Bernoulli beam theory at low AR is primarily due to the breakdown of the slender beam assumptions. For short pillars (low AR), transverse shear deformation, which is neglected in Euler-Bernoulli theory, becomes significant and comparable to bending deformation.

Given a constant adhesion stress threshold σ_{adh}, the pressure by the microjets needed for scaffold detachment is:

$$p \propto AR^{-2}$$

Assuming the effective acoustic pressure increases approximately with the square root of the selected bath power setting ($p_{applied} \propto \sqrt{P}$), the power threshold for lift-off of the scaffold pillars should theoretically follow a power law of

$$P_{applied} \propto AR^{-4}$$

To validate the scaffold lift-off mode, we compared our calculated average stress at the pillar base with the detachment thresholds reported by Izard et al[13], where the required detaching shear stress σ_{adh} for a scaffold with a footprint of 100 μm^2 were 12 MPa, which was then used as a detachment threshold in our FEM simulation (Figure S10b, red line). Because this value was reported for a specific scaffold geometry and interfacial system, it should be regarded here as a semi-quantitative reference rather than a universal detachment criterion. A purely analytical derivation of the power-AR relationship remains challenging. However, our experimental data (Figure 4) reveals a strong linear correlation on a logarithmic scale (log Sonication Power vs. log AR), demonstrating that the structural stability of the scaffold pillars drops sharply at higher aspect ratios. This trend is consistent with the theoretical AR^{-4} dependence of bending stress in

cantilever structures. To contextualize our experimental failure observations, we performed a parametric FEM sweep over the full scaffold array geometry used in the sample shown in Figure 4, varying both pillar aspect ratio and sonication power ($\propto p^2$) (Figure S10c).The resulting von Mises stress heatmap (Figure S10d) shows that, when 12 MPa (dashed line in Figure S10d) is taken as a reference interfacial detachment threshold, the experimentally observed failure boundary corresponds closely in order of magnitude to the cross-sectionally averaged stress at the pillar base. This analysis supports that the pillar detachment observed in the sample in Figure 4 is governed by interfacial stress failure between the pillar and the substrate, driven by flexural bending under directed lateral loading.

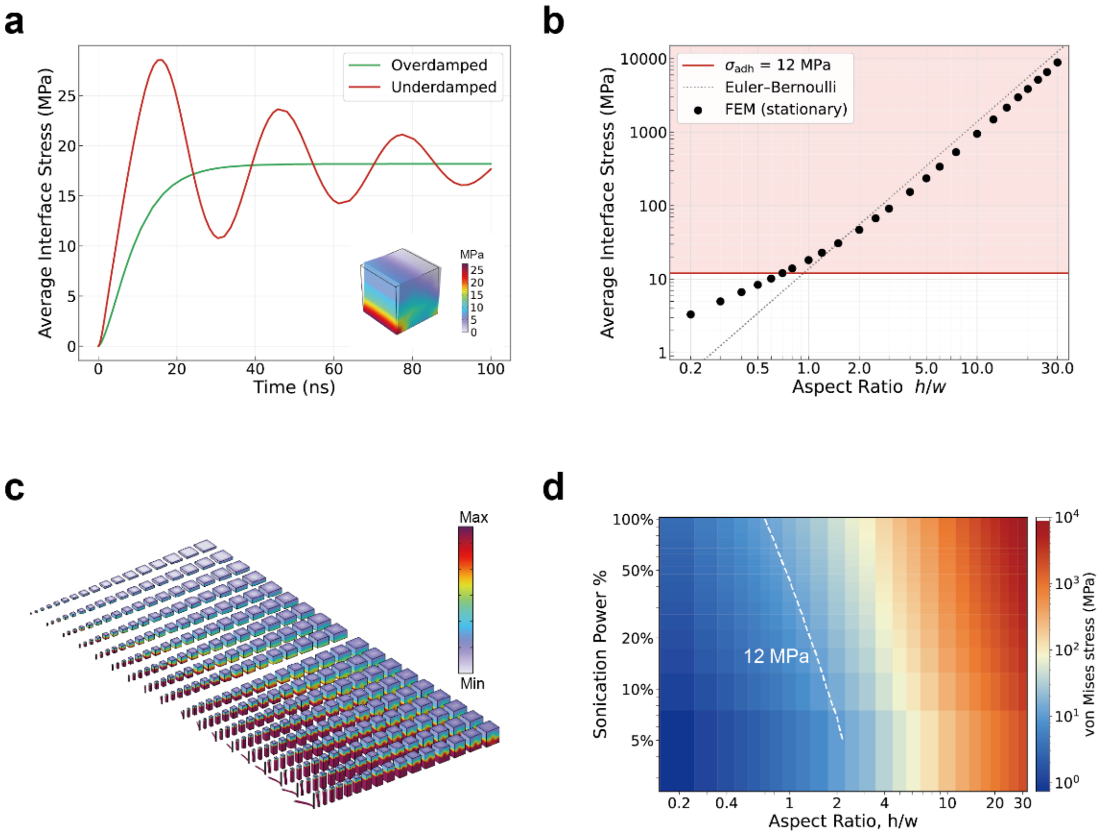

Figure S10. COMSOL simulation of resulting interface stress in dependence of sonication power and aspect ratio (AR) of scaffold pillars. a | Transient interfacial stress response of a single scaffold pillar (AR = 1) under a step pressure load of 9.2 MPa, simulated under two Rayleigh damping conditions (underdamped: $\beta = 1\times10^{-9}$ s; overdamped: $\beta = 5\times10^{-9}$ s). The peak dynamic stress is 1~2× the static value, depending on the damping condition, with the underdamped case approaching the theoretical dynamic amplification factor of 2. Inset: Representative von Mises stress distribution in the pillar. **b |** Stationary FEM simulations of average interfacial von Mises stress as a function of pillar aspect ratio (AR = h/w), with a uniform lateral step pressure load of 9.2 MPa applied to one sidewall. FEM results (filled circles) show good agreement with the Euler–Bernoulli analytical prediction (dotted line). The red horizontal line marks the experimentally measured adhesion strength (by Izard et al[13]) at the interface between the pillars and the substrate surface; pillars with aspect ratios above this threshold (shaded region) are predicted to detach. **c |** 3D rendering of the full scaffold array geometry (identical to the pillar arrays shown in Figure 4) used for parametric stress simulations, illustrating the variation in pillar height and stress distribution across the pillar array. **d |** Heatmap of simulated average von Mises stress at the pillar-substrate interface as a function of pillar aspect ratio and sonication power. The dashed white line marks the 12 MPa detachment threshold, in good agreement with the experimentally observed detachment boundaries of the pillars in the sample shown in Figure 4.

3. The Manuscript states that reduced scaffold-substrate adhesion is important (e.g., 10 nm SiO2 sputtered on printed polymer). Yet many demonstrations rely on substrate pre-treatments (APTES silanization for resin, SiO2 for polymer surfaces). The extent to which SALO is general across substrate chemistries is unclear. Please provide XPS/contact-angle data to characterize the surface treatments you used, and show whether a thin adhesion/intermediate layer (e.g., 10 nm SiO2) is generally required when patterning on polymer substrates or only beneficial in some cases.

Our response:

We thank the reviewer for this insightful comment. We agree that substrate chemistry plays a critical role in determining the lift-off mode of scaffold masks in the SALO process. To directly answer the reviewer question: A thin intermediate layer, such as 10 nm of sputtered SiO_2, is generally required when patterning scaffold masks on polymer or resin surfaces, whereas it is only an optional enhancement (or unnecessary) on inorganic surfaces (e.g., silicon or glass).

To address this comment and elucidate the importance of adhesion layers and surface treatments, we have included new contact angle measurements in the revised manuscript (Supplementary Figure S1), evaluating the effects of various surface treatments on the scaffold release process during SALO. Our results reveal that the lift-off mode of the scaffold mask (cohesive fracture of the scaffold structure vs. delamination of the scaffold from the substrate surface) is governed primarily by the interfacial adhesion between the scaffold mask and the substrate surface, rather than by differences in contact angle values of the substrate surface prior to scaffold deposition:

1. Scaffold masks on polymer/resin surfaces (a thin intermediate SiO_2 layer is required): When printing IP-Dip scaffolds directly on polymer surfaces, typically strong covalent cross-linking and polymer chain interpenetration[14] between the polymer surface and the P-Dip resin occur at the interface. This can create an interfacial bond between the polymer surface material and the scaffold materials that matches or exceeds the fracture strength of the scaffold material itself, inevitably leading to structural fracture within the scaffold in the lift-off process. Here, we introduced the 10 nm thick intermediate SiO_2 layer that acts as diffusion barrier between the surface polymer and the scaffold polymer. This layer physically blocks polymer chain interpenetration between the surface polymer and the scaffold polymer and provides in a reduced interfacial adhesion energy between the polymer scaffold and the intermediate SiO_2 layer (by hydrogen bonds and van der Waals interactions), thereby enabling clean detachment of the scaffold mask at the interface to the SiO_2 surface during lift-off.

2. Scaffold masks on inorganic surfaces (a thin intermediate SiO_2 layer is not required): For inorganic substrate surfaces such as O_2 plasma-treated glass or silicon, there is no additional intermediate SiO_2 layer required to reduce the interfacial adhesion energy between the substrate surface and the polymer scaffold to enable clean detachment of the scaffold during lift-off.

By adequately preparing the substrate surface we can achieve highly deterministic scaffold mask lift-off in the SALO process on chemically diverse substrate surfaces: Surface silanization is used to reliably anchor the scaffold structure on inorganic surfaces, whereas a thin intermediate SiO_2 layer is applied on polymer surfaces to facilitate clean scaffold detachment.

To address this comment in the revised Manuscript and Supplementary Information, we have added our new experimental data and analysis, including contact-angle characterization of surfaces after different surface treatments (Figure S1).

<u>Revised Manuscript, Page 3, Line 13</u>:

“We found that when printing the scaffold mask directly on a polymer surfaces, depositing a thin intermediate

inorganic layer, such as 10 nm of sputtered SiO_2, is generally required, acting as a diffusion barrier and preventing strong covalent cross-linking and polymer chain interpenetration between the polymer surface and the IP-Dip resin[14]. This approach confines the lift-off mode of the scaffold mask to clean interfacial delamination rather than fracture within the scaffold itself. To evaluate the influence of different substrate surfaces and surface pre-treatments on the lift-off behavior of the scaffold mask, we performed comparative contact-angle and scaffold adhesion experiments and found that the surface material and its pre-treatment are important factors for reliable scaffold lift-off (Supplementary section 1)."

Revised Supplementary Information, Page 2, Line 1:

"**Influence of substrate surface and surface pre-treatment on scaffold mask lift-off**

To evaluate the influence of different substrate surfaces on the lift-off behavior of the scaffold structures, we compared representative interfaces with different pre-treatments and chemistries, including O_2 plasma-cleaned glass, SiO_2-coated glass, TPP-printed polymer (IP-Dip), and silanized glass (Figure S1). For each case, we examined both the water contact angle before scaffold deposition and the resulting lift-off behavior during sonication. O_2 plasma-cleaned and SiO_2-coated surfaces with low water contact angles of ≤10° enabled clean interfacial detachment of the scaffold, whereas silanized surface (contact angle of ~50°) and printed polymer surfaces (contact angle of ~80°) featured much stronger scaffold adhesion and led to fracture of the scaffold near its base during sonication, rather than clean removal of the scaffold from the surface. These results indicate that the scaffold lift-off mode is governed primarily by the interfacial adhesion state between the scaffold and the surface material, rather than by macroscopic wettability alone, which agrees with previous studies by Izard et al. and Jelinek et al[13,15]. The observed strong scaffold adhesion on silanized surface and polymer surfaces is consistent with stronger interfacial interactions, potentially including covalent anchoring in the case of silanized surfaces and polymer-polymer interpenetration or photochemically induced coupling in the case of printed polymer surfaces[14]. The resulting interfacial bond strength can exceed the cohesive strength of the scaffold material, shifting the lift-off mode from interfacial debonding between the scaffold and the substrate surface, to structural fracture of the scaffold material near the scaffold base. Therefore, on polymer surfaces we used a deposited thin intermediate SiO_2 layer before scaffold printing to ensure well-defined scaffold lift-off."

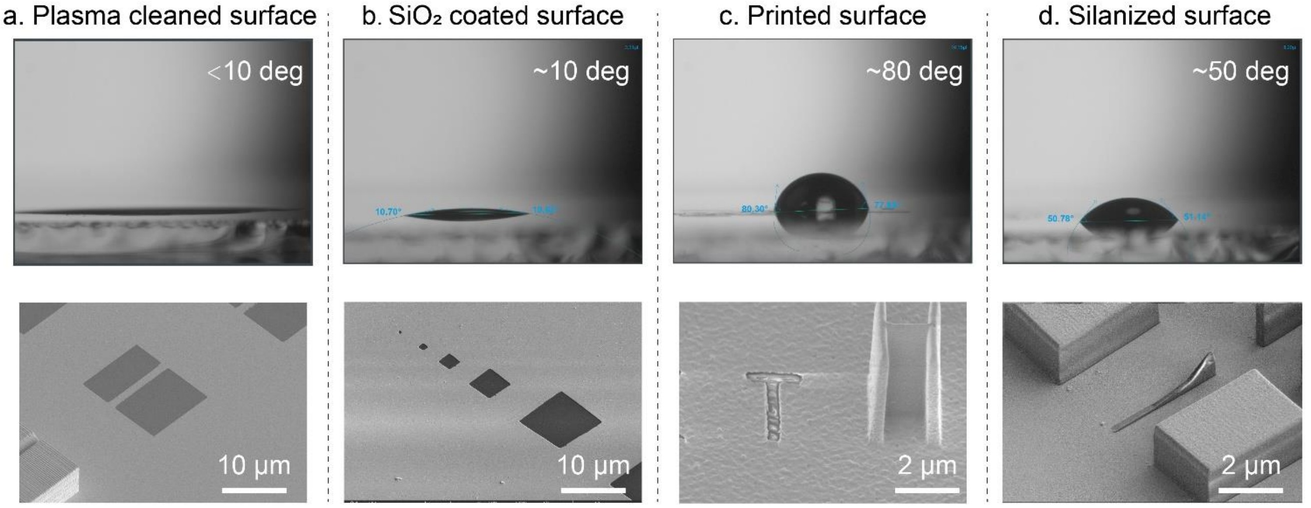

Figure S1. Correlation between substrate wettability and SALO behavior. Water contact angles were measured on four different surfaces based on ITO-coated glass substrate: (a) O_2 plasma-cleaned surface, <10°. (b) SiO_2-coated surface, ~10°. (c) 1 μm-thick TPP-printed polymer surface (IP-Dip), as printed, ~80°. (d) Silanized surface, ~50°. For (1) and (b), solid scaffolds with an aspect ratio (AR) of ~1 detached cleanly during sonication. For (c) and (d), solid scaffolds with an AR of ~1 did not release, while solid scaffolds with an AR of ~8 fractured at the base during sonication, instead of detaching from the substrate. Upper panels

show the water droplets during contact-angle measurements, and lower panels show the corresponding state of the scaffolds after sonication. The features visible in the two lower-right panels are fractured scaffold remnants remaining on the substrate after lift-off.

4. Cavitation in IPA and removal of polymer scaffolds may leave organic residues on the patterned film that can affect device performance (adhesion, contact resistance, optical loss). Provide surface chemical analysis (XPS, ToF-SIMS or FTIR) on patterned areas after SALO and after recommended rinsing steps, to show absence (or quantify level) of organic contamination. Include data for both small features and large area shell lifts.

Our response:

We thank the reviewer for this comment and we agree that verification of surface residues after scaffold removal is important for reproducibility and material quality.

To address this concern, we performed X-ray photoelectron spectroscopy (XPS) on samples after the lift-off and sample rinsing procedure. Specifically, we compared XPS spectra acquired from patterned and adjacent unpatterned regions on the same substrate under identical acquisition conditions. The survey scans and high-resolution C 1s spectra showed no detectable increase in carbon-related signals in the patterned areas relative to the unpatterned reference areas. The almost identical C 1s peak position and lineshape at both positions indicate that the SALO process does not introduce additional detectable organic residue within the sensitivity of our XPS measurements. We agree that it would be desirable to perform XPS verification on the smallest printed features, however in our XPS system the minimum selectable analysis aperture is 15 µm and for reliable chemical analysis, the area size has to be several times that of the nominal analysis diameter. Therefore, we performed point spectroscopy on 100 µm*100 µm patterns, which ensures that the acquired spectra predominantly originate from the patterned area rather than from the surrounding un-patterned surface.

We would like to note that for small scaffold features, our TPP printing process realizes solid scaffolds without enclosed volumes of non-crosslinked resin. Based on this design choice, we expect the patterned surface areas below the small scaffold features to be at least as clean (and likely cleaner) after lift-off as compared to the evaluated areas below the larger scaffold structures that were TPP printed using shell printing and containing non-crosslinked resin. To ensure proper representation of the XPS data, we explicitly state in the revised manuscript that direct XPS confirmation on the smallest features is limited by the lateral resolution of our instrument.

To address this comment in the revised Manuscript and Supplementary Information, we have included the new XPS measurements and a discussion of the results:

<u>Revised Manuscript, Page 8, Line 58</u>:

"To confirm that the SALO process and the subsequent cleaning procedure do not cause surface contaminations that could be problematic for subsequent processes, we used X-ray photoelectron spectroscopy (XPS) analysis and found no significant polymer contamination on the patterned surfaces (Supplementary section 13)."

<u>Revised Supplementary Information, Page 17, Line 1</u>:

"**Surface analysis after the SALO process**

To evaluate potential organic contamination following scaffold removal by sonication in an IPA bath, we performed X-ray photoelectron spectroscopy (XPS) measurements immediately after the SALO process and drying, without any additional plasma cleaning (Figure S12). In these experiments, we used a glass substrate

with an Indium Tin Oxide (ITO) surface coating and employed the SALO process to pattern a 20 nm thick sputtered Cr layer. The XPS measurements were carried out in ultrahigh vacuum ($<1\times10^{-8}$ Torr) using a monochromated ($h\nu$ = 1486.69 eV) Al Kα X-Ray source (Kratos Axis Supra+, Kratos Analytical Ltd, Manchester, UK) with charge neutralization. Binding energies were calibrated to C 1s peak at 285.0 eV. Survey spectra were collected at a power of 75 W, 40 mA emission current, while core level spectra and images were collected at a power of 600 W with varying acquisition time depending on required signal-to-noise.

To evaluate potential polymer residues, XPS analysis was performed on a SALO-patterned region (P1, exposed ITO) and an adjacent unpatterned reference region (P2, remaining Cr-coated surface) on the same substrate. The XPS survey spectra (Figure S12c) confirms the distinct surface compositions, showing strong In 3d signals in P1 and Cr 3p signals in P2. As shown in Figure S1c, the C 1s spectral shapes and peak positions on both the patterned and unpatterned surfaces are nearly identical. They predominantly consist of typical adventitious carbon features, dominated by C–C bonds at ~284.8 eV. Crucially, when compared to the reference spectrum of the printed IP-Dip photoresin (sample in Figure S1c), both P1 and P2 regions lack the distinct, intense oxygen-containing functionalities (such as C–O and C=O bonds) that are characteristic of the polymer[16,17]. These results indicate that no significant polymer residue is detectable by XPS on the tested SALO-patterned surfaces.

It is important to note that these measurements were conducted on 100 μm × 100 μm (P1) areas, which were patterned using a scaffold mask that was TPP printed using the shell printing mode. This area size was selected because reliable chemical analysis requires a targeted surface area that is significantly larger than the nominal beam diameter, which in our XPS system is 15 μm in spectroscopy mode. Analysis of small, patterned regions would result in signal convolution from the adjacent unpatterned areas. When smaller patterns are realized using the SALO process, the TPP printing mode results in solid scaffolds without enclosed volumes of uncrosslinked resin. Based on this design choice, we expect that these small, patterned areas are at least as clean (and likely cleaner) as the characterized areas (P1) for which large scaffold structures were used that were TPP printed using the shell printing mode, featuring scaffolds containing uncrosslinked resin.”

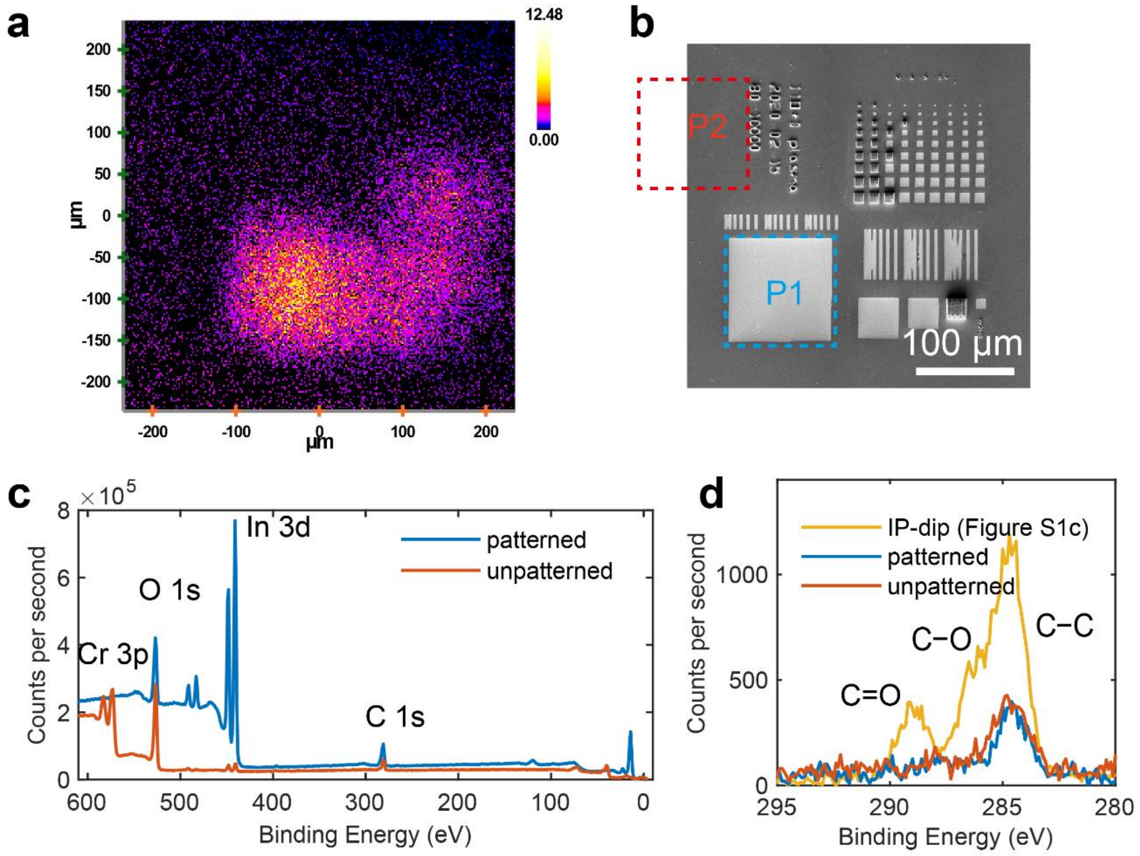

Figure S12. XPS analysis comparing carbon-based surface contamination of patterned vs unpatterned areas on an ITO-coated glass substrate. a | XPS surface map used to locate the patterned and unpatterned areas on the substrate, acquired at a fixed binding energy of 441.5 eV. The color scale indicates the photoelectron count rate (cps) in the sample plane. The substrate consists of an ITO-coated glass substrate (Nanoscribe standard substrate for 63x DILL mode) and the SALO patterned layer is 20 nm of sputtered Cr. The Indium signal serves as an indicator of the exposed ITO surface. **b** | SEM image of the XPS sample showing the patterned region and the measurement locations, two representative positions (P1 and P2) were selected for point spectroscopy. **c** | XPS survey (wide-scan) spectra acquired from patterned and unpatterned areas (step size: 1 eV), providing an overview of elemental composition. **d** | High-resolution C 1s spectrum acquired with a 0.1 eV step size to assess carbon-based surface species and residual organics in the two areas, compared with reference sample of the printed IP-Dip photoresin (sample in Figure S1c). A baseline correction was applied to the spectrum for comparison.

5. The shell type scaffolds rely on uncrosslinked interior resin that must remain uncrosslinked until sonication. The authors note that ambient light over days can crosslink these regions and prevent removal. This is an important practical limitation. Please provide quantitative tests: how long can shell type scaffolds be stored in ambient light/dark/packaged conditions before lift off remains successful? Does ambient humidity or temperature affect this window? Is intermediate oxygen plasma always an effective rescue step? These are critical process control data for users.

Our response:

We thank the reviewer for this constructive comment. In response to this comment, we performed new experiments, systematically varying storage conditions prior to performing the SALO process (light exposure, pressure, humidity, and temperature) to assess their effect on the lift-off process. Under all evaluated conditions, the shell scaffolds resulted in successful lift-off, and we observed no measurable degradation in lift-off success rate or structural integrity, indicating that the overall fabrication window is reasonably tolerant under standard laboratory handling. While lift-off remained successful in all cases, the amount of residues on the patterned areas after performing the SALO process depended primarily on the light-exposure history of the scaffolds during storage between the TPP printing and performing the lift-off process. For freshly printed scaffolds processed immediately, SEM inspection showed no residues. After 10 days of storage under ambient laboratory light at room temperature, we observed residues localized at the bottom of the shell scaffold, consistent with partial curing of trapped uncrosslinked resin during storage. In contrast, samples stored in the dark showed significantly less residues under otherwise comparable conditions. Within the evaluated parameter range, we did not observe a clear trend attributable to humidity or temperature. As a mitigation strategy, we found that a brief intermediate O_2 plasma clean (Cobra 100, Oxford instrument; RF power 20 W; pressure 50 mTorr; O_2 flow 20 sccm; substrate temperature 20 °C; 2 min) effectively removed the polymer residues after performing the lift-off process.

Finally, we clarify an important caveat related to sample handling: Some early experiments were performed during summer, and we later realized that incidental sunlight exposure during sample transfer between labs can accelerate unintended curing of uncrosslinked resin trapped inside shell-printed scaffolds. We now clearly distinguish such sunlight exposure from exposure to “standard ambient laboratory light” in the revised Manuscript.

To address this comment in the revised Manuscript and Supplementary Information, we have included the new experimental results and the revised the Manuscript text accordingly.

<u>Revised Manuscript, Page 8, Line 52</u>:

“To evaluate the practical process window of the SALO process using shell-type scaffolds, we performed

storage experiments of scaffold masks prior to performing the lift-off process (Supplementary section 14), confirming that samples with shell-type scaffolds remained fully capable of successful lift-off even after 20 days of storage in a standard laboratory environment, provided they are shielded from direct sunlight. We found that exposure to direct sunlight can accelerate unintended curing of the resin inside the shell of the scaffold. Any polymer residues remaining after lift-off of the scaffold mask induced by prolonged exposure to ambient light can be removed using an O2 plasma cleaning step."

Revised Supplementary Information, Page 19, Line 1:

"**Effects of storage conditions of shell-type scaffolds on lift-off results**

To determine the practical processing window of shell-printed scaffold masks and to assess whether storage before lift-off increases the risk of residual uncrosslinked resin, we carried out a controlled storage study on samples after scaffold printing and prior to the SALO process. Representative SEM images in Figure S13 show patterned areas after the lift-off process was performed on samples with freshly printed scaffold masks, and for samples in which the scaffold masks were stored for 10 or 20 days under four different conditions, respectively: (1) Ambient laboratory light at room temperature (23 °C) in air (30% RH). (2) Dark at room temperature (23 °C) in air (30% RH). (3) Dark at 3 °C in air (30% RH). (4) Ambient laboratory light at room temperature (23 °C) under vacuum (10% RH). All samples used 10 μm × 10 μm × 10 μm scaffolds printed in shell mode on an ITO-coated glass substrate to pattern a 20 nm thick sputtered Cr layer.

As shown in Figure S13, under all evaluated conditions, the shell scaffolds successfully lifted off without any measurable degradation of the resulting pattern. However, the presence of polymer residues remaining in the patterned areas (prior location of the scaffolds) was primarily dependent on light exposure during storage of the samples after scaffold printing and before lift-off. While samples based on freshly printed scaffolds (row 1, fresh) exhibited no significant polymer residues after the lift-off process, samples with scaffolds stored under ambient laboratory light for 10 and 20 days (row 1 and row 4, 10 and 20 days) showed minor polymer residues, consistent with the partial curing of the trapped uncrosslinked resin. In contrast, storing the samples in a dark environment (row 2 and row 3) markedly reduced these polymer residues after lift-off of the scaffold. Within the evaluated range, no clear and reproducible effect of temperature or humidity on the amount of residual polymer was observed. These results indicate that light exposure during storage of samples carrying shell-printed scaffolds, prior to lift-off, is the dominant factor promoting residual polymer formation in the lift-off patterned areas. As shown in the rightmost column of Figure S13, a brief intermediate oxygen plasma clean (RF power 20 W, 50 mTorr, O_2 flow 20 sccm, 2 min) serves as a highly effective and universal rescue step to remove any residual organic traces induced by prolonged curing.

Finally, we found that incidental exposure to direct sunlight of samples with shell-printed scaffold structures must be strictly avoided, as the high-intensity UV component of sunlight can rapidly cure the uncured resin encapsulated by the shell structure, and compromise the subsequent lift-off process."

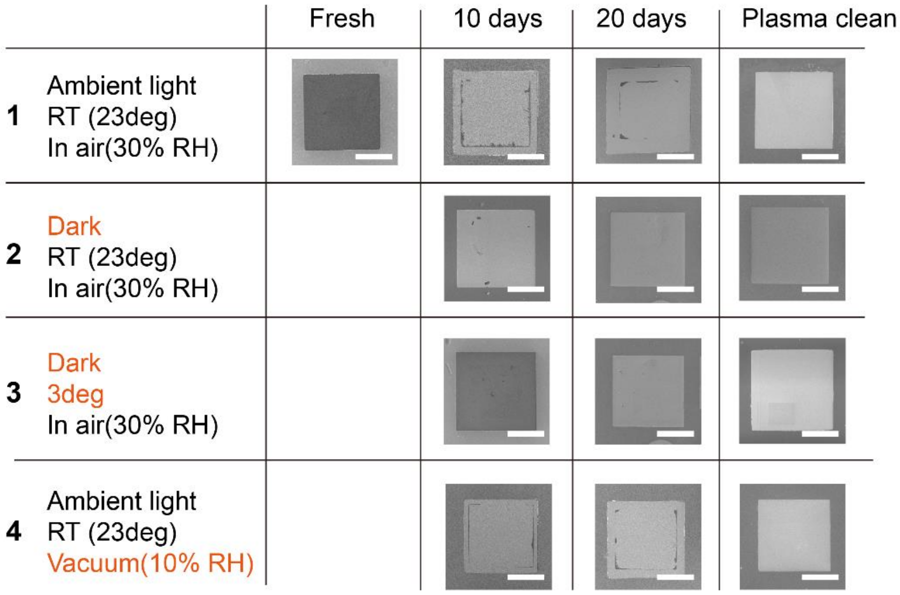

Figure S13. Storage conditions of shell-printed scaffold masks and its effects on polymer residues from the scaffold structures after the lift-off process. Representative SEM images of SALO-patterned areas after the scaffold lift-off are shown for samples processed (film deposition and lift-off) immediately after scaffold printing ("Fresh") or stored after scaffold printing and before film deposition and lift-off under different environmental conditions. Row **1**: Ambient laboratory light at room temperature (23 °C) in air (30% RH). Row **2**: Dark at room temperature (23 °C) in air (30% RH). Row **3**: Dark at 3 °C in air (30% RH). Row **4**: Ambient laboratory light at room temperature (23 °C) under vacuum (10% RH). Storage condition differences were marked in orange. Columns correspond to samples processed fresh, or after storage for 10 or 20 days, followed by lift-off. The rightmost column ("Plasma clean") shows the corresponding samples after a brief intermediate O_2 plasma treatment. Fresh samples are shown only for condition 1. All samples used ITO-coated glass substrates with shell mode printed scaffolds (10 μm × 10 μm × 10 μm) to pattern 20 nm thick sputtered Cr films. Scale bar 5 µm.

6. Most significant thing is, the so-called 3D surfaces are printed with the masks, while all the 3D surfaces needed to be coated with a metal layer is initially 3D surfaces. The situations of this paper is useless unless the authors can printed the masks on an existed 3D structures. Or, many methods can fabricate this masks if the substrate should be fabricated first.

Our response:

We thank the reviewer for this critical comment, which touches upon the core value proposition of our work. We fully agree that for a patterning method to be practically useful on "3D surfaces," it must demonstrate the capability to define masks directly onto pre-existing, non-planar substrates, rather than merely creating 3D resist structures on a flat wafer.

To directly address this concern, we emphasize that our method does exactly what the reviewer suggests is necessary. In our work we explicitly demonstrate high-resolution patterning on a variety of 3D structures and surface materials. For instance, we successfully applied the SALO process to silicon pyramids featuring steep 54.7° sidewalls that were fabricated using KOH etching (Figure 2c, 3a, 6a). We also demonstrated conformal patterning over convex dome-shaped 3D silicon structures (Figure S2). These examples prove that our SALO workflow is highly effective for patterning on complex substrates where the 3D topography exists prior to any mask definition. Regarding the experiments where both the 3D substrate and the scaffold masks were printed from polymers, we clarify that these were intentionally designed to systematically evaluate the geometric

adaptability of the SALO process across extreme topographical variations. To avoid any possible confusion for readers, we have revised the Manuscript to clearly distinguish the demonstrations on 3D silicon structures from those 3D surface structures that were printed to evaluate morphological versatility.

To address this comment in the revised Manuscript, we have in the introduction included to explicitly distinguish our demonstrations on pre-existing 3D substrates from those on printed topographies.

Revised Manuscript, Page 2, Line 15:

"We demonstrate the capabilities of our approach by lift-off patterning of diverse thin films on a range of 3D surfaces, including silicon pyramids with steep 54.7° sidewalls, dome-shaped silicon structures, glass, and TPP-printed 3D polymer structures."

Revised Supplementary Information, Page 2, Line 17:

"Collectively, to demonstrate the capability of defining scaffold masks on pre-existing 3D topographies, we successfully applied the SALO process to initially non-planar 3D substrates, including KOH-etched silicon pyramids, convex silicon domes, and dimensionally constrained end-face of an optical fiber."

***Some other minor comments are as follows*:**

7. Provide more complete sonication details: bath model and geometry are given, but please add: sample placement (center/edge), use of degassed IPA or not, bath fill volume, whether samples were fixed or floating, actual power calibration method (how the "%" maps to W), and bath temperature during runs. These details are important for reproducibility.

Our response:

We thank the reviewer for this comment. We agree that these operational details are important for reproducibility, and our original Methods description did not provide sufficient information to allow direct replication across different ultrasonic baths and laboratories.

To address this comment in the revised Manuscript, we have updated the Methods part about sonication.

Revised Manuscript, Page 14, Line 54:

"Lift-off experiments were conducted using a benchtop ultrasonic bath (VWR USC300D, Avantor, Inc., USA) operating at 45 kHz. The bath provides nine discrete power settings from 10% to 100% of a nominal maximum electrical output power of 80 W. Because the instrument reports power as a percentage, all sonication conditions are reported as the selected setting (%) rather than an assumed delivered acoustic power (W). Sonication was performed in IPA at room temperature.

Unless otherwise stated, the ultrasonic bath was filled with IPA to a fixed liquid volume of 20 mL and allowed to equilibrate to room temperature (~23 °C) before use. Samples were immersed in a glass beaker containing IPA and placed at the center of the bath, with the beaker supported on a holder so that the sample is not in direct contact with the bottom of the bath. IPA was used as received, non-degassed prior to use, across all experiments. The bath temperature was monitored during sonication (monitored at the start and end of each 2 min sonication interval) and maintained within 22–27°C."

8. However, the Manuscript does not provide an in-depth explanation of why mechanical lift-off can overcome the continuous sidewall coverage of ALD films without causing fractures. It is recommended to analyze the interfacial structure at the lift-off edge via cross-sectional TEM or FIB cross-sectioning to verify whether film fracture or a specific delamination mode occurs at the scaffold edges.

Our response:

We thank the reviewer for this comment. We fully agree that the ability to pattern highly conformal, continuous ALD films without causing random tearing or film fractures at the loft-off edges, is an important feature of the SALO process that warrants a deeper mechanistic explanation.

The fundamental reason that ALD films avoid catastrophic fractures in the SALO process lies in where the stress concentrates in the lift-off process. Unlike traditional solvent-based lift-off processes that dissolve the supporting resist mask and then aggressively tear the unsupported thin films, SALO relies on a localized shear force applied directly to the high-aspect-ratio scaffold mask. This macroscopic lateral force translates into an extreme stress concentration precisely at the base of the scaffold. Consequently, crack propagation is strictly confined to the mechanically weakest path, which is the deliberately weakened interface between the scaffold mask and the substrate surface, rather than propagating upward into the bulk of the conformal ALD film. The ALD layer on the surrounding substrate remains continuously supported and undisturbed during this highly localized interfacial debonding and lift-off process.

To visually validate this fracture mechanics model, we have performed the suggested FIB cross-sectioning at the lift-off edge of an ALD ZnO film (Supplementary Figure S5). The cross-sectional images provide unambiguous and direct evidence of the clean interfacial structure. The retained ALD film exhibits a continuous morphology and an intact interface with the underlying substrate, with no obvious micro-cracking or delamination observed in the inspected region, and no plastic deformation at the edges of the patterns.

To address this comment in the revised Manuscript and Supplementary Information, we have incorporated both a discussion of the lift-off mechanics and the new FIB cross-sectional analysis.

<u>Revised Manuscript, Page 5, Line 18</u>:

"The localized interfacial debonding of the scaffold mask cleanly shears off the patterned features along the scaffold perimeter without transferring destructive tensile stresses to the surrounding thin film, thereby enabling the patterning of highly conformal ALD coatings with clean edges and no evident cracking in the inspected cross-sections (see Supplementary Figure S5)."

<u>Revised Supplementary Information, Page 6, Line 1</u>:

"Thin film integrity at the pattern edges

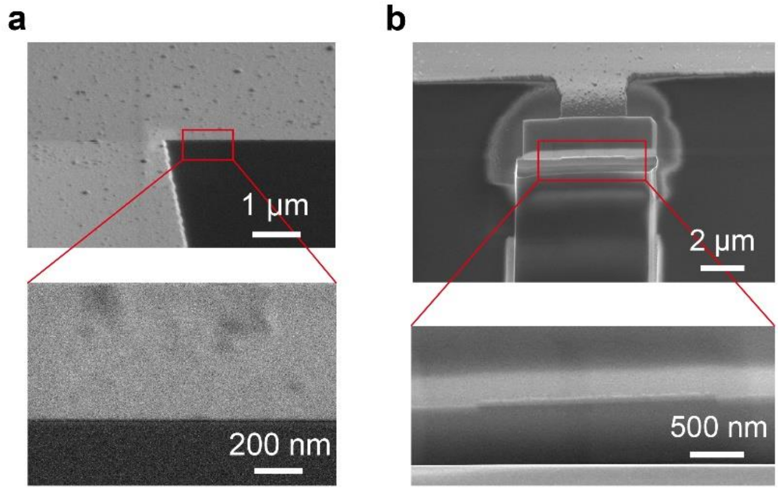

Figure S5. Edge integrity of SALO-patterned ALD films. a | Close-up SEM image of a SALO-patterned 25 nm ALD ZnO film, highlighting the edges of the pattern. No evidence of film delamination, cracking, or film discontinuity is observed. b | FIB-milled cross-sectional image of the patterned ZnO film at the edge region, confirming conformal film coverage and an intact film-substrate interface without observable

delamination.”

9. The Manuscript mentions that shell-type scaffolds must be protected from ambient light to prevent unwanted crosslinking. This is a notable practical constraint that should be emphasized in the main text, not only in the Methods.

Our response:

We thank the reviewer for this comment. We agree that the light sensitivity of the shell-type scaffolds is a key practical consideration for reproducibility and should be highlighted in the main text rather than being confined to the Methods section.

To address this comment in the revised Manuscript, we have added a dedicated sentence in the revised Manuscript to explicitly emphasize that shielding the samples from prolonged ambient light prior to the lift-off step is essential for preventing the unintended curing of the trapped internal resin.

<u>Revised Manuscript, Page 8, Line 50</u>:

“It should be noted that to ensure optimal lift-off reliability, shell-type scaffolds should be stored under yellow-light lithography or dark conditions prior to sonication. This prevents prolonged ambient UV light from inadvertently curing the trapped liquid resin.”

10. The excellent performance of SALO is demonstrated with several materials (Ag, Ni, Ta, Hf, Au, Al_2O_3, ZnO). Were there any specific material combinations (scaffold/thin-film/substrate) or film stress states that proved challenging for the SALO process, leading to delamination or incomplete lift-off? Are there material properties that would make a system incompatible with SALO?

Our response:

We thank the reviewer for this question. While the SALO process is highly versatile across various materials, we have identified that failure cases are almost exclusively governed by insufficient thin film adhesion to the substrate or inherently unfavorable stress in the thin film, rather than by the mechanical lift-off mechanism itself. We did not observe obvious SALO failure in the sputtered Cr films (high tensile stress) we evaluated, suggesting that film stress alone is not necessarily a problem if the film adheres well to the substrate surface. For example, highly tensile sputtered Cr films were successfully patterned without edge fracture or delamination. This robustness is consistent with our successful patterning of continuous ALD coatings: As long as the thin film securely adheres to the underlying substrate, the localized shear stress at the scaffold boundary cleanly breaks the film without propagating cracks into the retained thin film regions. For metals with low adhesion properties, such as Ag and Au, the strong shear forces during the SALO process can cause unintended delamination of the retained film regions if the metal is deposited directly onto the substrates (as illustrated in Supplementary Figure S15a). However, this limitation can be mitigated by adding a thin adhesion layer such as Ti or Cr, which avoids any delamination problems when patterning the thin Au layers using SALO.

To address this comment in the revised Manuscript and Supplementary Information, we added Supplementary section 16 and Figure S15 which discusses and illustrates three representative failure mechanisms, their physical origins, their practical limitations on the process, and the corresponding mitigation strategies.

<u>Revised Manuscript, Page 13, Line 24:</u>

“A summary of potential failure modes arising from poor adhesion of the deposited thin films, reflection-induced exposure non-uniformity when TPP printing the scaffold mask, and incomplete resist development of the TPP-printed scaffold mask is provided in Supplementary section 16 and Figure S15.”

Revised Supplementary Information, Page 22, Line 1:

“**SALO process failure modes**

Summary of three representative SALO failure mechanisms and mitigation strategies

1. Thin Film Delamination

- Mechanism & Limitation: When depositing metals with weak surface adhesion directly onto certain inert substrates, such as the sputtered Au films shown here, the interfacial adhesion energy between the Au layer and the substrate is often overcome by the internal stress of the thin film and/or the mechanical force applied during the ultrasonic lift-off process. This can result in delamination or tearing of the deposited thin film at the pattern edges (Figure S15a).
- Mitigation: This is a fundamental issue relating to thin film adhesion rather than specific to the SALO process. This failure mechanism is readily mitigated by optimized the thin film deposition process, such as the use of an adhesion layer (e.g., 2-5 nm of Ti or Cr) prior to depositing the metal layer (here Au).

2. Laser Exposure Dose Non-uniformity Due to Reflective Surfaces During Scaffold Printing

- Mechanism & Limitation: Two-photon polymerization (TPP) is sensitive to local light intensities. When printing on reflective substrate surfaces, such as reflective metal films, the focused laser beam interferes with laser light reflected by the substrate surface, creating standing waves and localized intensity hotspots at the substrate interface. This proximity effect can induce unintended over-crosslinking or “scalloping” of the photoresist near the base of the scaffold, which can severely distort and compromise the scaffold geometry (Figure S15b). This effect limits the applicability of SALO on reflective mirror-like surfaces if no mitigation measures are taken.
- Mitigation: The optical interferences caused by reflective surfaces can be mitigated by applying a thin anti-reflective coating (ARC)[18,19] prior to TPP printing, or by implementing adaptive exposure strategies that dynamically reduce the laser power precisely at the substrate interface to compensate for the energy of the reflected laser light.

3. Incomplete Development of the Photoresist Near the Base of the Scaffold

- Mechanism & Limitation: Incomplete photoresist development can result in non-crosslinked photoresist residues trapped near the base of the scaffold (i.e., the interface critical for the SALO process). The resist residues locally prevent coverage of the substrate surface with the deposited thin film, thereby compromising pattern integrity (Figure S15c). Incomplete resist development can, for example, be caused by restricted transport of the liquid developer (e.g., PGMEA) in scaffolds with high aspect-ratio or dense geometries.
- Mitigation: This limitation can be overcome by optimizing the resist development protocol. Specifically, extending the development time or introducing gentle agitation of the developer bath can improve removal of unexposed photoresist from dense, high-aspect-ratio regions.

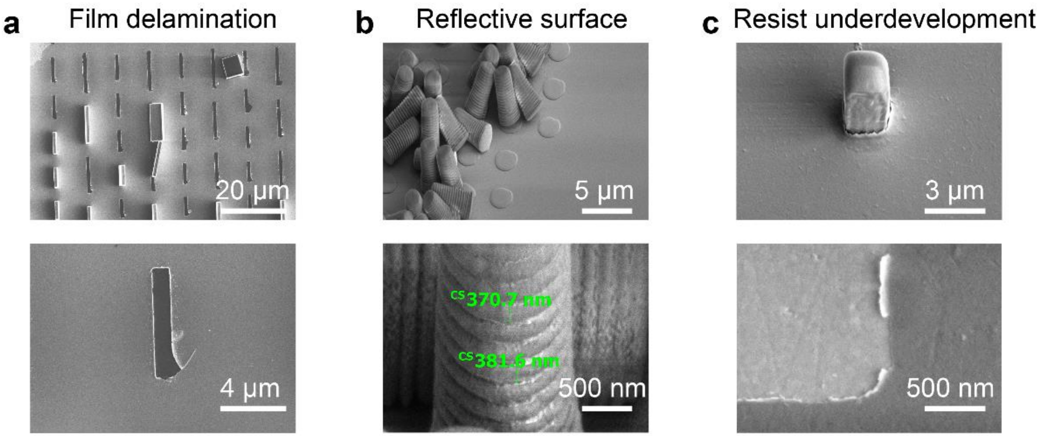

Figure S15 | Possible failure modes and their causes. a| Representative example of observed delamination of a SALO-patterned 25 nm thick sputtered Au film without Ti or Cr adhesion layer. b| Effects of exposure dose non-uniformity near the scaffold base during scaffold TPP printing caused by reflections from reflective substrate surface (here sputtered 20 nm Cr), which can compromise the scaffold mask (upper image) by unintended photoresist cross-linking or scalloping (lower image). c| Example of incomplete scaffold-mask development leaving resist residues at the scaffold base (here after sputtering 20 nm of Cr). The resist residues can shadow the thin film deposition (upper image) and cause residual flakes at the edges of the patterned film after the SALO process (lower image).

11. The process requires the substrate with the printed scaffold to be conformally coated. For structures with extreme topographies, such as deep trenches or high-aspect-ratio pillars, could the scaffold itself shadow the deposition, leading to non-uniform coating on the substrate near the scaffold's base? If so, how can this be mitigated in the scaffold design?

Our response:

We thank the reviewer for raising this important practical consideration. We fully agree that extreme 3D topography can introduce geometric shadowing, particularly near the scaffold base during highly directional deposition processes such as physical vapor deposition (PVD). The severity of this shadowing effect is fundamentally dependent on the chosen deposition technique and can be effectively managed through the geometric flexibility of the TPP printed scaffolds, for example by intentionally designing the scaffold geometries to avoid severe overhangs. In contrast, in surface-reaction-limited processes such as ALD, conformal coverage is maintained more readily than in directional PVD processes, including in the high-aspect-ratio geometries evaluated here. This conformal capability is intrinsically resistant to shadowing and is clearly demonstrated in our successful ALD patterning results (Supplementary Figure S6).

To address this comment in the revised Manuscript and Supplementary Information, we have added a profile measurement of an ALD-coated thin film on high-aspect-ratio scaffolds, confirming uniform coating thickness. We have also added a brief sentence in the main text referencing these new results.

<u>Revised Manuscript, Page 5, Line 21:</u>

"When patterning over complex 3D topographies, deposition non-uniformity can become a critical consideration. For surface-reaction-limited deposition processes such as ALD, however, the coatings are highly uniform, even in high-aspect-ratio scaffold geometries (Supplementary Figure S6). For more directional thin film deposition processes such as PVD, potential geometric shadowing can be mitigated through scaffold geometry design, as discussed in Supplementary Information section 6"

<u>Revised Supplementary Information, Page 7, Line 1</u>:

“**Uniform thin film coverage**

For highly recessed or high-aspect-ratio scaffold mask geometries, surface-reaction-limited deposition processes such as ALD avoid the geometric shadowing associated with line-of-sight coating processes, resulting in uniform film thickness across the scaffold profile, as shown in Figure S6. In contrast, when more directional line-of-sight thin film coating processes are used, such as PVD, the overhangs, sidewall angles and spacings of the scaffold mask become important design parameters for ensuring thin film coverage near the scaffold base.”

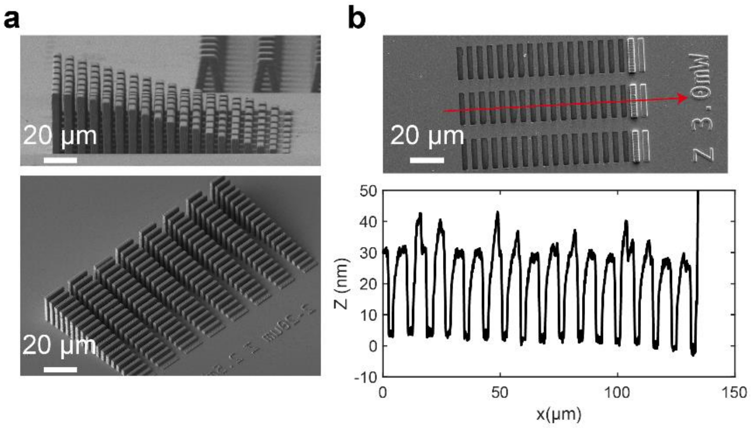

Figure S6 | High-aspect-ratio scaffold geometry and conformal film coverage. (a) SEM images of representative high-aspect-ratio scaffolds fabricated by TPP printing. (b) Top-view SEM of SALO-patterned 25 nm ZnO thin film which was deposited inside the deep trench of the very-high aspect ratio scaffolds. The red arrow indicates the line-scan direction and the corresponding height profile, showing a constant ALD coating thickness of about 25 nm across the gaps that were surrounded by scaffolds with different aspect ratios. The spikes in the line-scan are due to particles at the surface (confirmed by SEM imaging) that were present before the ALD layer was deposited.

Reviewer #2 (Remarks to the Author):

This Manuscript introduces SALO, which is a scaffold-architected lift-off technique with the aim to enable high-resolution patterning of conformal thin films (in particular including ALD) on complex 3D surfaces. The approach is indeed original, well demonstrated, supported by strong imaging and well communicated. Crucially, the ability to pattern ALD on steep or even vertical surfaces is particularly worth mentioning. Although really impressive, several aspects need clarification before publication in Nature Communications.

Our response:

We thank the reviewer for the positive evaluation of our Manuscript. We have addressed all comments below and revised the Manuscript accordingly.

1. Mechanistic basis of lift-off is a bit underrepresented: The key parameter (h/w > 1) is well supported by empirical data. However, physical explanation remains more qualitative. A deeper rationale or discussion of limitations would strengthen the claim of generality. I know this is challenging. However, at least a comment in that direction would be needed.

Our response:

We sincerely thank the reviewer for raising this important point regarding the mechanistic basis of the lift-off process. We completely agree that establishing a solid physical and mechanical rationale is crucial for understanding the process boundaries.

To address this, we have incorporated a detailed semi-quantitative mechanical model and corresponding numerical analyses into the revised Manuscript and Supplementary Information. Briefly, our analysis demonstrates that the structural failure (lift-off) of the scaffold is primarily governed by flexural bending induced by the directed momentum of ultrasonic cavitation microjets, rather than by uniform shock wave pressure. Our experimental data confirms that structural stability of the scaffold drops as the aspect ratio of the scaffold features increases, aligning with the theoretical dependence of bending stress in cantilever structures.

To address this comment in the revised Manuscript and Supplementary Information, we have added our new simulation results, analysis and discussions.

<u>Revised Manuscript, Page 8, Line 38</u>:

"The localized structural damage and eventual lift-off of the pillars are most consistently explained by the bending moments induced by cavitation microjet impingement (Supplementary Figure S10 and section 11.1) This mechanical fracture mode is further supported by semi-quantitative finite element simulations, which align closely with the experimentally observed damage patterns (Supplementary section 11.2)"

<u>Revised Manuscript, Page 15, Line 9</u>:

"**Finite element simulations**

Stationary finite element simulations were performed using COMSOL Multiphysics 6.4 (COMSOL AB, Stockholm, Sweden). Each scaffold pillar was modeled as a square cross-section cantilever beam with a fixed base footprint of 5 × 5 μm^2 and varying height to systematically sweep the aspect ratio (AR = h/w). The pillar material was assigned the linear elastic properties of IP-Dip photoresin, with a Young's modulus of 2.9 GPa and a Poisson's ratio of 0.38, as specified by the manufacturer. A uniform pressure load was applied to one lateral face of the pillar to represent the equivalent hydrodynamic loading from cavitation microjet impingement. The base of the pillar was assigned a fixed boundary condition to represent the rigid substrate

interface. The von Mises stress was spatially averaged over the entire basal cross-section and used as the metric for evaluating interface detachment. A free tetrahedral mesh with a maximum element size of 1 μm was used throughout the pillar volume to ensure sufficient spatial resolution, especially at the interface region between the pillar and the substrate."

Revised Supplementary Information, Page 12, Line 1:

"**Modelling and simulations**

To examine whether the proposed microjet-driven mechanism for lift-off is consistent with the observed lift-off behavior of the scaffold during sonication, we developed a semi-quantitative mechanical model evaluating the hydrodynamic forces exerted by ultrasonic cavitation. In ultrasonic surface cleaning, the violent collapse of asymmetric cavitation bubbles near a solid surface generate high-velocity microjets, which are widely recognized as the primary hydrodynamic mechanism responsible for dislodging surface particles and structures[6,7]. In our experiments, we observed a strong and systematic aspect ratio (AR)-dependence of the scaffold micro-pillars that are detached during sonication: High-AR pillars consistently failed at lower sonication power than lower-AR pillars. This selective behavior is consistent with a mechanism involving lateral bending moments, which scale with AR^2, rather than compressive loading from isotropic shock waves. Therefore, based on our analysis, as outlined below, using established ultrasonic cleaning models and our experimental observations, we believe that microjet-induced lateral impact is likely the dominant physical driver for the observed structural failure of the scaffolds during sonication.

11.1. Validation of Microjet Impingement Model

For ultrasonic cavitation with frequencies in the tens of kilohertz regime, reported microjet velocities are on the order of 100 m/s in water[7,8], and the corresponding impact duration is therefore expected to be in the sub-microsecond to microsecond range based on the characteristic jet length. In isopropanol (IPA), the jet velocity is expected to be lower than in water because the higher vapor pressure can modify cavitation dynamics. The microsecond-scale load is within a timescale at which the dynamic bending response mode of a pillar-shaped scaffold mask becomes relevant. Based on the pillar dimensions of the scaffold used in this study, the natural oscillation period of the pillars is estimated to be of the order of tens to hundreds of nanoseconds using Euler-Bernoulli cantilever beam theory, comparable to the duration of the water hammer phase, suggesting that dynamic effects may partially contribute to the peak stress. Specifically, when the impact duration closely matches the structure's natural period, it triggers dynamic amplification, resulting in a transient peak stress that is significantly higher than the stress caused by an equivalent static load.

Our transient simulations (Supplementary Figure S10a) show a clear dynamic response of the pillars when applying a step pressure load, where the peak stress can be amplified by a factor of maximum 2 (no damping in the system) due to inertial effects, compared to the static case. To assess the transient structural response of the pillar under impulsive microjet loading, we performed time-dependent simulations using Rayleigh damping with the stiffness-proportional coefficient beta set to 5×10^{-9}, and 1×10^{-9} s, representing overdamped and underdamped conditions, respectively. Since the dynamic stress amplification factor under sudden step loading is limited to a maximum of 2 for undamped systems, and decreases with increasing damping, the static pressure simulations adopted here represent a conservative lower bound.

To evaluate the structural integrity of the micro-pillars under the influence of cavitation-induced microjets, the impact of a microjet is modeled as a sustained drag force. According to the classification by Liu et al[9], the first phase of the jet impact is characterized by the water hammer pressure:

$$p_{hammer} \approx \rho c v$$

where $\rho \approx 786 \mathrm{kg} \cdot \mathrm{m}^{-2},\ c = 1170 m \cdot s^{-1}$ for IPA, and v is the microjet velocity in dependence of the sonication power.

To approximate the hydrodynamic loading relevant to the SALO process, we selected a microjet velocity within the lower end of values reported for near-wall cavitation-driven flows. Prior studies have reported jet-impact or jet-associated local flow velocities ranging from several meters per second to several tens of meters per second in controlled cavitation systems, including about 5~80 m/s [10] in near-wall bubble studies and local flow velocities on the order of 10 m/s [11] near the tip of a laser-generated microjet. Because the local microjet velocity in a laboratory ultrasonic bath is highly transient and difficult to determine directly, we used 10 m/s as a conservative order-of-magnitude estimate rather than as a direct measurement of the flow field in our system. The scaffold lift-off process can be modeled as a two-stage physical process: First, upon the initial impingement of the microjet, a transient water hammer pressure ($P_{WH} = \rho c v \approx 9.2$ MPa for IPA) delivers an intense, impulsive shock. This causes instantaneous high-stress concentrations at the edges of the base of the pillar, crack formation by effectively exceeding the interfacial adhesion strength between the pillar and the substrate surface, and dislodging the IP-Dip pillar scaffolds at their base. Second, the transient shock dissipates into a steady radial liquid flow with a much lower stagnation pressure ($P_{dyn} = 0.5\rho v^2 \approx 39$ kPa). Rather than inducing further structural fracture, this residual hydrodynamic flow primarily serves to flush away the already detached scaffolds, clearing them from the underlying thin films and completing the lift-off process[12].

11.2. Corroboration with Experimental Scaling Laws

The most compelling evidence for the hypothesis that the lift-off threshold of the scaffold pillars is governed by beam-bending mechanics is its strong dependence on the aspect ratio (AR) of the pillars, which is consistent with beam theory mechanics. For a cantilever beam under a distributed pressure p (representing the microjet impact), the maximum stress at the pillar base (at the interface between the pillar and the substrate surface) scales as:

$$\sigma_{max} = \frac{M \cdot c}{I} = 3p \cdot \frac{h^2}{w^2}$$

where M = (p * w * h) * (h/2) is the bending moment; c = w/2 is the distance to the neutral axis; I = $w^4/12$ is the area moment of inertia; h is the pillar height and w is the pillar width. However, since the interfacial failure stress between IP-Dip photoresist and glass, as measured by Izard et al[13], represents the spatial average over the entire cross-section of the area of the pillar base, and the bending stress is linearly distributed across the neutral axis of the pillar, the cross-sectionally averaged stress magnitude σ_{ave} is theoretically predicted to be 1/2 of the maximum stress.

$$\sigma_{ave} = 1.5p \cdot AR^2$$

This is consistent with our FEM simulation results shown in Supplementary Figure S10b, validating our simulation framework. The deviation between the FEM results and the analytical Euler-Bernoulli beam theory at low AR is primarily due to the breakdown of the slender beam assumptions. For short pillars (low AR), transverse shear deformation, which is neglected in Euler-Bernoulli theory, becomes significant and comparable to bending deformation.

Given a constant adhesion stress threshold σ_{adh}, the pressure by the microjets needed for scaffold detachment is:

$$p \propto AR^{-2}$$

Assuming the effective acoustic pressure increases approximately with the square root of the selected bath power setting ($p_{applied} \propto \sqrt{P}$), the power threshold for lift-off of the scaffold pillars should theoretically follow a power law of

$$P_{applied} \propto AR^{-4}$$

To validate the scaffold lift-off mode, we compared our calculated average stress at the pillar base with the detachment thresholds reported by Izard et al[13], where the required detaching shear stress σ_{adh} for a scaffold with a footprint of 100 μm^2 were 12 MPa, which was then used as a detachment threshold in our FEM simulation (Figure S10b, red line). Because this value was reported for a specific scaffold geometry and interfacial system, it should be regarded here as a semi-quantitative reference rather than a universal detachment criterion. A purely analytical derivation of the power-AR relationship remains challenging. However, our experimental data (Figure 4) reveals a strong linear correlation on a logarithmic scale (log Sonication Power vs. log AR), demonstrating that the structural stability of the scaffold pillars drops sharply at higher aspect ratios. This trend is consistent with the theoretical AR^{-4} dependence of bending stress in cantilever structures. To contextualize our experimental failure observations, we performed a parametric FEM sweep over the full scaffold array geometry used in the sample shown in Figure 4, varying both pillar aspect ratio and sonication power ($\propto p^2$) (Figure S10c).The resulting von Mises stress heatmap (Figure S10d) shows that, when 12 MPa (dashed line in Figure S10d) is taken as a reference interfacial detachment threshold, the experimentally observed failure boundary corresponds closely in order of magnitude to the cross-sectionally averaged stress at the pillar base. This analysis supports that the pillar detachment observed in the sample in Figure 4 is governed by interfacial stress failure between the pillar and the substrate, driven by flexural bending under directed lateral loading.

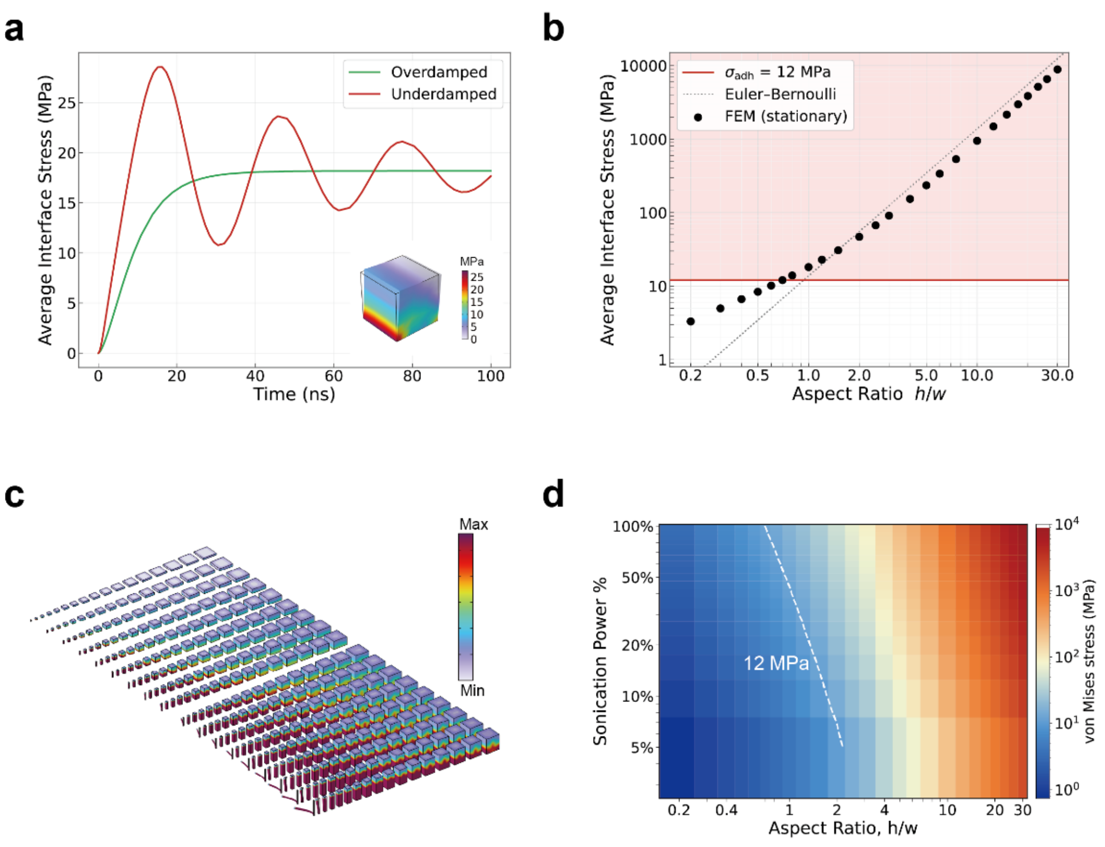

Figure S10. COMSOL simulation of resulting interface stress in dependence of sonication power and aspect ratio (AR) of scaffold pillars. a | Transient interfacial stress response of a single scaffold pillar (AR = 1) under a step pressure load of 9.2 MPa, simulated under two Rayleigh damping conditions (underdamped: $\beta = 1\times10^{-9}$ s; overdamped: $\beta = 5\times10^{-9}$ s). The peak dynamic stress is 1~2× the static value, depending on the damping condition, with the underdamped case approaching the theoretical dynamic amplification factor of 2. Inset: Representative von Mises stress distribution in the pillar. **b |** Stationary FEM simulations of average interfacial von Mises stress as a function of pillar aspect ratio (AR = h/w), with a uniform lateral step pressure load of 9.2 MPa applied to one sidewall. FEM results (filled circles) show good agreement with the Euler–

Bernoulli analytical prediction (dotted line). The red horizontal line marks the experimentally measured adhesion strength (by Izard et al[13]) at the interface between the pillars and the substrate surface; pillars with aspect ratios above this threshold (shaded region) are predicted to detach. **c |** 3D rendering of the full scaffold array geometry (identical to the pillar arrays shown in Figure 4) used for parametric stress simulations, illustrating the variation in pillar height and stress distribution across the pillar array. **d |** Heatmap of simulated average von Mises stress at the pillar-substrate interface as a function of pillar aspect ratio and sonication power. The dashed white line marks the 12 MPa detachment threshold, in good agreement with the experimentally observed detachment boundaries of the pillars in the sample shown in Figure 4.

2. ALD-patterning claims need more convincing evidence: ALD is a true major highlight, but the examples are kind of limited. I wonder whether the authors have aspect for inclusion such as (i) film integrity after lift-off; (ii) performance on highly challenging geometries or (iii) information on potential failure modes (e.g. delamination or crack formation). That would strengthen that really important case. At least a comment would be needed.

Our response:

We thank the reviewer for this constructive comment. We agree that ALD patterning is a central highlight and that additional evidence and explicit discussion of limitations will strengthen the manuscript. We therefore performed new experiments and analysis.

Related to part (i): We performed additional SEM and FIB cross-sectional analysis of patterned ALD films to verify the thin film integrity and pattern fidelity after lift-off.

Related to part (ii): We performed additional experiments on ALD deposition on high aspect-ratio scaffold structures and analyzed the pattern fidelity and ALD film uniformity. Together with our detailed experiments on SALO patterning of ALD coatings on slanted scaffold masks and capped closed-mask geometries (Supplementary Section S4), these data now provide a broader and more convincing experimental basis that our process is suitable for patterning of ALD layers on challenging 3D geometries.

Related to part (iii): We discuss three representative failure mechanisms of the SALO process, their physical origins, their practical limitations on the process, and the corresponding mitigation strategies.

To address these comments in the revised Manuscript and Supplementary Information, we have incorporated the above listed new experiments and analysis.

Related to part (i):

Revised Manuscript, Page 5, Line 18:

"The localized interfacial debonding of the scaffold mask cleanly shears off the patterned features along the scaffold perimeter without transferring destructive tensile stresses to the surrounding thin film, thereby enabling the patterning of highly conformal ALD coatings with clean edges and no evident cracking in the inspected cross-sections (see Supplementary Figure S5)."

Revised Supplementary Information, Page 6, Line 1:

"Thin film integrity at the pattern edges

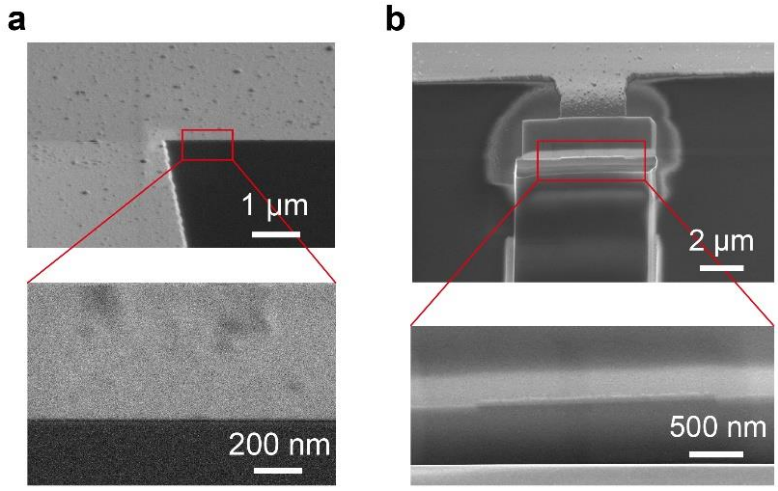

Figure S5. Edge integrity of SALO-patterned ALD films. a | Close-up SEM image of a SALO-patterned 25 nm ALD ZnO film, highlighting the edges of the pattern. No evidence of film delamination, cracking, or film discontinuity is observed. b | FIB-milled cross-sectional image of the patterned ZnO film at the edge region, confirming conformal film coverage and an intact film-substrate interface without observable delamination."

Related to part (ii):

Revised Manuscript, Page 5, Line 21:

"When patterning over complex 3D topographies, deposition non-uniformity can become a critical consideration. For surface-reaction-limited deposition processes such as ALD, however, the coatings are highly uniform, even in high-aspect-ratio scaffold geometries (Supplementary Figure S6). For more directional thin film deposition processes such as PVD, potential geometric shadowing can be mitigated through scaffold geometry design, as discussed in the Supplementary Information section 6."

Revised Supplementary Information, Page 7, Line 1:

"**Uniform thin film coverage**

For highly recessed or high-aspect-ratio scaffold mask geometries, surface-reaction-limited deposition processes such as ALD avoid the geometric shadowing associated with line-of-sight coating processes, resulting in uniform film thickness across the scaffold profile, as shown in Figure S6. In contrast, when more directional line-of-sight thin film coating processes are used, such as PVD, the overhangs, sidewall angles and spacings of the scaffold mask become important design parameters for ensuring thin film coverage near the scaffold base."

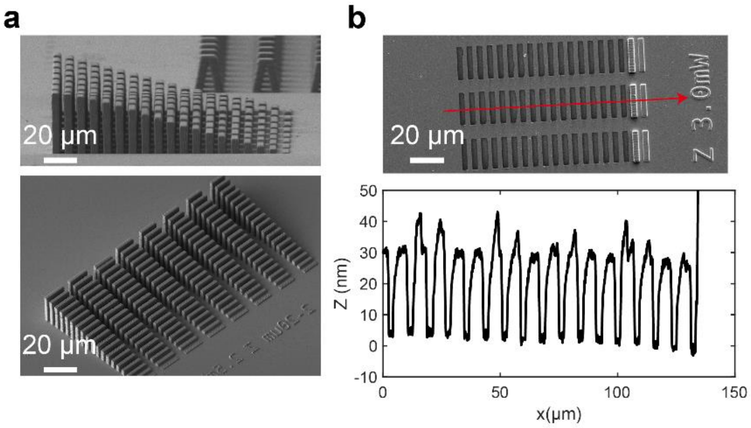

Figure S6 | High-aspect-ratio scaffold geometry and conformal film coverage. (a) SEM images of representative high-aspect-ratio scaffolds fabricated by TPP printing. (b) Top-view SEM of SALO-patterned 25 nm ZnO thin film which was deposited inside the deep trench of the very-high aspect ratio scaffolds. The red arrow indicates the line-scan direction and the corresponding height profile, showing a constant ALD coating thickness of about 25 nm across the gaps that were surrounded by scaffolds with different aspect ratios. The spikes in the line-scan are due to particles at the surface (confirmed by SEM imaging) that were present before the ALD layer was deposited.

Related to part (iii):

Revised Manuscript, Page 13, Line 24:

"A summary of potential failure modes arising from poor adhesion of the deposited thin films, reflection-induced exposure non-uniformity when TPP printing the scaffold mask, and incomplete resist development of the TPP-printed scaffold mask is provided in Supplementary section 16 and Figure S15."

Revised Supplementary Information, Page 22, Line 1:

"**SALO process failure modes**

Summary of three representative SALO failure mechanisms and mitigation strategies

1. Thin Film Delamination

- Mechanism & Limitation: When depositing metals with weak surface adhesion directly onto certain inert substrates, such as the sputtered Au films shown here, the interfacial adhesion energy between the Au layer and the substrate is often overcome by the internal stress of the thin film and/or the mechanical force applied during the ultrasonic lift-off process. This can result in delamination or tearing of the deposited thin film at the pattern edges (Figure S15a).
- Mitigation: This is a fundamental issue relating to thin film adhesion rather than specific to the SALO process. This failure mechanism is readily mitigated by optimized the thin film deposition process, such as the use of an adhesion layer (e.g., 2-5 nm of Ti or Cr) prior to depositing the metal layer (here Au).

2. Laser Exposure Dose Non-uniformity Due to Reflective Surfaces During Scaffold Printing

- Mechanism & Limitation: Two-photon polymerization (TPP) is sensitive to local light intensities. When printing on reflective substrate surfaces, such as reflective metal films, the focused laser beam interferes with laser light reflected by the substrate surface, creating standing waves and localized

intensity hotspots at the substrate interface. This proximity effect can induce unintended over-crosslinking or "scalloping" of the photoresist near the base of the scaffold, which can severely distort and compromise the scaffold geometry (Figure S15b). This effect limits the applicability of SALO on reflective mirror-like surfaces if no mitigation measures are taken.

- Mitigation: The optical interferences caused by reflective surfaces can be mitigated by applying a thin anti-reflective coating (ARC)[18,19] prior to TPP printing, or by implementing adaptive exposure strategies that dynamically reduce the laser power precisely at the substrate interface to compensate for the energy of the reflected laser light.

3. Incomplete Development of the Photoresist Near the Base of the Scaffold

- Mechanism & Limitation: Incomplete photoresist development can result in non-crosslinked photoresist residues trapped near the base of the scaffold (i.e., the interface critical for the SALO process). The resist residues locally prevent coverage of the substrate surface with the deposited thin film, thereby compromising pattern integrity (Figure S15c). Incomplete resist development can, for example, be caused by restricted transport of the liquid developer (e.g., PGMEA) in scaffolds with high aspect-ratio or dense geometries.
- Mitigation: This limitation can be overcome by optimizing the resist development protocol. Specifically, extending the development time or introducing gentle agitation of the developer bath can improve removal of unexposed photoresist from dense, high-aspect-ratio regions.

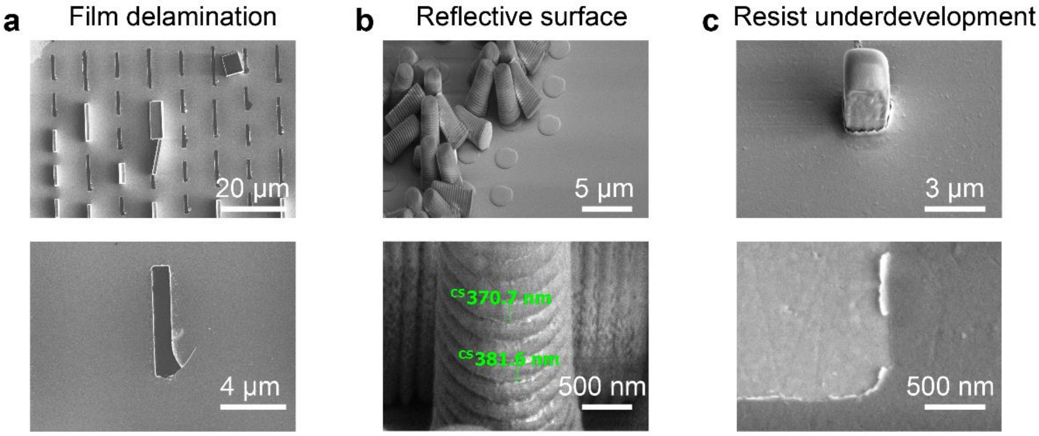

Figure S15 | Possible failure modes and their causes. a| Representative example of observed delamination of a SALO-patterned 25 nm thick sputtered Au film without Ti or Cr adhesion layer. b| Effects of exposure dose non-uniformity near the scaffold base during scaffold TPP printing caused by reflections from reflective substrate surface (here sputtered 20 nm Cr), which can compromise the scaffold mask (upper image) by unintended photoresist cross-linking or scalloping (lower image). c| Example of incomplete scaffold-mask development leaving resist residues at the scaffold base (here after sputtering 20 nm of Cr). The resist residues can shadow the thin film deposition (upper image) and cause residual flakes at the edges of the patterned film after the SALO process (lower image).

3. Resolution and fidelity quantification is too shallow: Although the presented SEM images look truly excellent and impressive, the Manuscript somehow lacks aspects like statistical relevant information of feature deviation, a discussion of possible error sources and in best case more examples on very small features. Inclusion by either images or at least a short discussion would be really important because resolution is a central claim in that frame.

Our response:

We thank the reviewer for this comment. We agree that quantitative resolution and fidelity analysis is important because resolution is a central claim of the Manuscript.

To address this comment in the revised Manuscript and Supplementary Information, we have included additional examples and statistical metrology data on sub-micrometer patterns, together with a discussion of the dominant error sources in the fabrication process.

Revised Manuscript, Page 6, Line 18:

"We further quantified pattern fidelity and feature-to-feature variation in sub-micrometer circular and square patterns fabricated in a 20 nm thick Cr layer on a glass substrate and found that features with nominal diameters down to 400 nm can be reproducibly defined, with measured standard deviations below 40 nm across the evaluated nominal dimensions (Supplementary section 7 and Figure S7). "

Revised Supplementary Information, Page 8, Line 1:

"**Patterning accuracy of sub-micrometer scale features**

Using TPP printed (IP-Dip) scaffolds on a glass substrate and subsequent SALO-patterning of a 20 nm thick sputtered Cr film, we quantified the deviation between the nominal pattern diameter of the scaffold mask and the actual feature size of the resulting circular pattern in the thin film. The results show that circular features with nominal diameters down to 400 nm can be reproducibly defined, with measured standard deviations below 40 nm across the evaluated nominal dimensions (Figure S7). For pillar diameters smaller than 400 nm, the printed scaffold approaches a dose-defined pillar-like regime where the effective feature size and its standard deviation depend heavily on primarily by voxel size and exposure conditions rather than the CAD design.

We also show that reliable shape differentiation is maintained for feature sizes of approximately 1 μm for the evaluated scaffold geometries, whereas at smaller dimensions the voxel geometry and proximity effects increasingly dominate the shape of the printed structure (see circle vs square comparison in Figure S7a).

Importantly, the ultimate resolution and dimensional fidelity are limited by the specific TPP laser printing system used and by the printing process calibration, since the voxel size and printing accuracy depend on multiple interconnected parameters, such as the exposure dose, system optics, and proximity effects, and these parameters vary across TPP tools and photoresists."

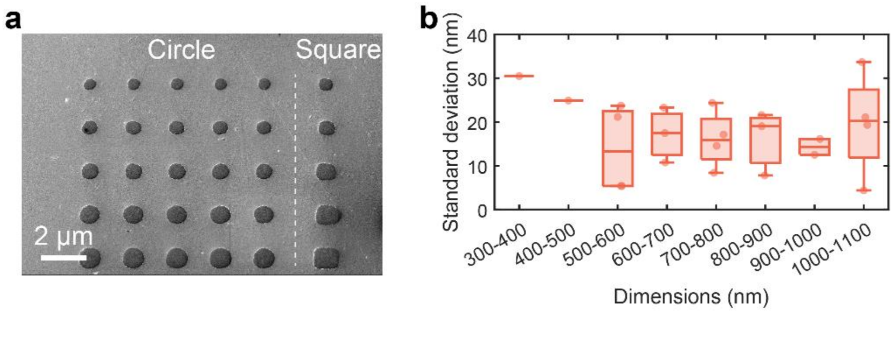

Figure S7 | Dimensional accuracy of patterned features and variability at the sub-micrometer scale. a| SEM image of representative sub-micrometer sized circular and square patterns used for dimensional metrology (scale bar: 2 μm). b| Standard deviation of the measured diameter of circular patterns as a function of the nominal diameters, showing dimensional variability across the evaluated range of pattern diameters. At least five different pattern samples were measured for each nominal diameter to calculate the standard deviation.

4. Proof-of-concept devices: The nanowire and fiber-tip grating are truly impressive and confirm patterning. However, I wonder whether the authors have also information on the device performance, which should be very strong. Hence, if the authors have information in that direction, please include them or have a least a discussion about further plans.

Our response:

We thank the reviewer for this encouraging comment and for recognizing the strength of the nanowire and fiber-tip grating demonstrations. We agree that demonstrator device performance is a crucial metric. We would like to clarify that functional validation data for the two proof-of-concept devices are reported in the Manuscript: The electrical continuity of the nanowires is confirmed via resistance measurements, and the structural fidelity of the fiber-tip gratings is validated by their distinct optical diffraction patterns. We realize that these results could be highlighted more clearly, and we have revised the main text in the Manuscript to point the reader more explicitly to these results. It should be noted that we deliberately positioned these demonstrations as basic proof-of-concept validations rather than fully optimized, standalone devices. The optimization of state-of-the-art nanowire sensors and diffraction gratings typically requires extensive efforts that go beyond the scope of our current work. Our primary goal in this Manuscript is not to claim the best possible device performance of the demonstrator devices, instead we focus on the demonstration of versatile patterning capabilities on 3D topographies and structures that are notoriously difficult to achieve using current technologies.

To address this comment in the revised Manuscript and Supplementary Information, we have included a discussion and clarifications about the proof-of-concept devices:

<u>Revised Manuscript, Page 11, Line 18:</u>

<u>New passage of **Realization of Functional Structures**, changes highlighted in yellow:</u>

"For the nanowire demonstration we patterned a gold nanowire on a silicon substrate surface with sloped sidewalls by KOH etching (slope sidewall angle of 54.7°). The sputtered nanowire, consisting of a 5 nm thick Cr adhesion layer and a 90 nm thick Au layer features a width of 900 nm and a total length of 1080 µm and is placed on the sloped surface and crossing the sharp edge of the sloped surface (Figure 6a, right image). We performed electrical measurements of the nanowire via a voltage sweep from –500 mV to +500 mV. The nanowire exhibited a resistance of 3.01 kΩ (equivalent to 6.2 Ω·m), slightly higher than the 2.58 kΩ (equivalent to 5.2 Ω·m) of a reference sample consisting of an identical nanowire design placed on a planar surface (Figure 6a, left image). Both current-voltage (I–V) curves showed good linearity, indicating reliable film continuity and electrical contacts, and the measured characteristics are consistent with those of comparable nanowire devices reported in the literature[20,]. The increased resistance in the 3D nanowire is likely due to minor non-uniformities in coating thickness or roughness near sharp edges, which could be further mitigated by improving the etching smoothness of the underlying silicon structures.

For the demonstration of the optical wire-grid gratings patterned on an optical fiber tip (Figure 6b), we used a single-mode optical fiber with a 125 µm cladding diameter (Thorlabs, Inc. USA). We patterned a 30 nm thick Au grating using ion sputtering (SEC Co., Ltd., Korea), featuring a pitch of 3 µm and a linewidth of approximately 1.5 µm conformally on the fiber end. We carried out the optical characterization of the grating by inserting light in the fiber using a near-infrared (NIR) laser diode with a wavelength of 1550 nm, with the diffraction pattern of the light exiting the fiber tip being recorded by an NIR camera placed 1 cm in front of the fiber tip. The resulting pattern exhibited a curvature consistent with results from our simulations (Supplementary Figure S14), confirming the structural and optical fidelity of the patterned grating. The optical response of the wire-grid gratings also is consistent with the behavior of comparable devices reported in the literature[21]. Beyond this specific demonstration, 3D photonics appears to be a particularly promising application space for SALO; for instance, ALD has previously been integrated with 3D printed micro-optics to produce highly conformal anti-reflective coatings without compromising the underlying surface topography[19]. Combined with SALO, this conformal coating capability could enable the fabrication of more advanced 3D photonic architectures.

Together, our electrical and optical demonstrators establish the SALO process as a viable route for fabricating functional, high-resolution devices. Nanowire resistance measurements and optical diffraction patterns confirm the expected electrical and optical performance, underscoring the validity of the approach as a proof of concept. Beyond these demonstrations, our method is readily extendable to a wide range of photonic, electronic, and sensing devices requiring reliable patterning of conformal thin films on arbitrarily shaped 3D surfaces.

5. Scalability and throughput: The usual questions for the readership are: (i) what are realistic print times; (ii) is wafer-scale patterning really feasible; or (iii) what about strategies for scaling up the process. A short discussion would broaden the Manuscript essentially.

Our response:

We thank the reviewer for this excellent suggestion. We agree that scalability and throughput are important for assessing practical adoption, and we added a related discussion in the Manuscript and a table in the Supplementary Information summarizing representative print times and scaling strategies.

(i) What are realistic print times: A scaffold for a complex 3D surface typically requires on the order of 10 to 15 min to print over a patterned area of 100 µm × 100 µm, whereas a small device such as a fiber tip grating requires tens of seconds to a few minutes. These print times are conservative because our current workflow prints the full scaffold volume using high resolution settings. In practice, the scaffold mask only requires high fidelity in the parts critical for the pattern outline near the interface between the scaffold and the surface, while the bulk of the scaffold volume can in principle be written with coarser hatching and slicing to increase throughput. Such variable resolution writing strategies are established in TPP printing and can be easily implemented.

(ii) Is wafer-scale patterning really feasible; or (iii) what about strategies for scaling up the process: While the present work serves as a proof-of-concept on smaller substrates, the patterning concept is fully

compatible with future scale-up using emerging high-throughput TPP printing platforms. There are a number of strategies to develop the laser lithography tools towards high-throughput TPP 3D printing: For example, the utilization of digital micromirror devices (DMD) by Somers et al. enable parallel multi-beam laser patterning without compromising diffraction-limited resolution[22]. This laser beam parallelization was subsequently massively scaled up by Ouyang et al. using digital holography to operate 2,000 programmable laser foci simultaneously, achieving a printing speed of 2.0×10^6 voxels/s and volumetric processing rates up to 54.0 mm^3/hour.[23] Most recently, a breakthrough employing metalens arrays and spatially adaptive illumination has pushed the printing speed to an unprecedented 1.49×10^8 voxels/s[24] (Gu et al., *Nature*, 648, 591-599, 2025). This specific advancement overcomes the fundamental trade-off between spatial resolution and macroscopic throughput, enabling the high-throughput fabrication of complex, fully aperiodic 3D nanostructures directly across macroscopic wafer-scale areas. By adopting these state-of-the-art ultrafast TPP laser writing platforms, the fabrication of SALO scaffolds can be scaled to wafer level and industrially relevant speed.

To address this comment in the revised Manuscript and Supplementary Information, we have included a discussion of the TPP printing speed and emerging strategies towards increased TPP printing speed:

Revised Manuscript, Page 13, Line 34:

"While printing the scaffold masks in the current work typically takes of the order of tens of minutes (for exact printing times see Supplementary section 10). Scaffold mask writing by TPP can be readily scaled to speeds that are feasible for printing on wafer-scale areas using emerging high-throughput printing strategies based on parallelized laser beam generation[22–24]. Most recently, metalens-array-enabled TPP printing with spatially adaptive illumination has pushed parallel laser writing beyond 10^8 voxels s^{-1},[24] making this approach in principle feasible for wafer-scale and industrially relevant applications. By leveraging these readily available nanofabrication tools, we anticipate that wide adoption and exploration of SALO will pave the way for new possibilities in fabricating sophisticated 3D micro- and nanostructures for a diverse scientific and industrial applications."

Revised Supplementary Information, Page 24, Line 1:

"**Scaffold mask printing times and scalability**

As detailed in Table S2, the fabrication times for the various scaffold masks demonstrated in our study takes of the order of tens of minutes, reflecting the inherent throughput limitations of conventional serial, single laser beam TPP printing. While patterning scaffold masks on a full 100 mm or 200 mm diameter wafer using our direct write TPP laser tool would be prohibitively slow, the SALO lift-off process scheme is compatible with wafer-scale substrates. To achieve throughputs suitable for wafer-level scaffold-mask printing, recent hardware advances in laser direct writing provide a viable path to substantially increased laser writing speeds. For example, the utilization of digital micromirror devices (DMD) by Somers et al. enable parallel multi-beam laser patterning without compromising diffraction-limited resolution[22]. This parallelization was subsequently massively scaled up by Ouyang et al. using digital holography to operate 2,000 programmable laser foci simultaneously, achieving a printing speed of 2.0×10^6 voxels/s and volumetric processing rates up to 54.0 mm^3/hour.[23] Most recently, a breakthrough employing metalens arrays and spatially adaptive illumination has pushed the printing speed to an unprecedented 1.49×10^8 voxels/s[24]. By adopting such state-of-the-art ultrafast TPP laser writing strategies, the fabrication of SALO scaffold masks is readily scalable to wafer level and industrially relevant speed."

Table S2: Representative scaffold mask printing times (non-optimized printing processes).

Scaffold	Printing Area	Printing Speed	Printing Time
Figure 1	130 µm × 130 µm	1 000 µm/s	15 min
Figure 2d	140 µm × 140 µm	1 000 µm/s	18 min
Figure 3a	100 µm × 600 µm	1 000 µm/s	13 min
Figure 5c	20 µm × 20 µm	1 000 µm/s	7 min
Figure 6a	200 µm × 400 µm	10 000 µm/s	10 min
Figure 6b	30 µm × 60 µm	10 000 µm/s	20 s

Reviewer #3 (Remarks to the Author):

The work provides significant developments in combining lithography and lift-off tools, pushing them towards the 3D realm at micro- and nano-scales. The experimental study is sufficiently novel and substantial. On the other hand, it is too much hyped and overstressed with some points, which are not accurate and sometimes even contradictory. Thus, it needs a revision.

Our response:

We thank the reviewer for the positive assessment of the novelty and overall contribution. We also appreciate the feedback of the reviewer regarding potential contradictions and overstatements. We have carefully revised the Manuscript to moderate the language, remove overstated claims, and ensure that all statements are consistent and supported by data.

1. IP-Dip resin was chosen. Why specifically, and what benefits and limitations does it have for the reported study? Would other, let's say pure single acrylate or hybrid materials such as ORMOCERs or ORMOSILs, have advantages due to their different adhesion and resilience? The choice of specific material should be clearly introduced, and a comparison with alternatives could be provided. The simple claim that the IP-Dip has weak adhesion to the substrate is not qualitatively nor quantitatively provided, nor is the influence of the substrate discussed. It appears that adhesion changes could allow for tuning/extending the release during lift-off, correct?

Our response:

We thank the reviewer for this insightful comment. We agree that the choice of IP-Dip should be better motivated and that alternative TPP resins, including single acrylates and organic inorganic hybrid materials, can be relevant for SALO. In this work, our choice of IP-Dip is grounded in its well-established balance of ultra-high optical resolution[25] (sub-200 nm feature sizes) and robust intrinsic mechanical properties. We clarify that IP-Dip was used as a practical starting point rather than a unique requirement for SALO.

We agree that hybrid organic inorganic materials such as ORMOCER based resins can offer advantages in thermal and mechanical robustness, and they have a strong track record in TPP micro-optics and photonic structures. We now cite representative prior work on two TPP of ORMOCER type hybrids and note that such materials are promising candidates to extend the SALO processing window toward harsher deposition and post processing conditions. While systematic, quantitative adhesion mapping across various resin and substrate combinations is beyond the scope of this initial proof-of-concept study, we have explicitly identified it as an important direction for future optimization.

The reviewer also accurately points out the critical aspect of substrate adhesion. Previous studies have reported relatively weak adhesion of IP-Dip on untreated silicon and glass surfaces[13,15]. While this poor adhesion is typically considered a limitation for permanent 3D micro-structures, it is a significant advantage in our SALO process**.** The relatively weak interfacial bonding of IP-Dip allows for a clean and efficient lift-off (release) without requiring aggressive chemical etches that might damage the underlying surface or the deposited thin films. In our experiments, IP-Dip provided sufficiently reproducible scaffold fabrication and release behavior for SALO. To address the influence of substrate surface chemistry on adhesion, we performed additional experiments to compare lift-off behavior across different surface chemistries and included the new experimental data in the Manuscript.

To address this comment in the revised Manuscript and Supplementary Information, we have revised the text to more clearly motivate the choice of resist, discuss alternative resist materials and included the new experimental data.

Revised Manuscript, Page 2, Line 42:

"We selected the proprietary acrylic photoresist IP-Dip as scaffold mask material in our experiments because this resist is optimized for ultra-high-resolution 3D patterning in our employed TPP laser lithography tool Photonic Professional GT2 system (Nanoscribe GmbH, Germany). Conveniently for the SALO workflow, IP-Dip exhibits intrinsically weak adhesion to untreated silicon and SiO_2 surfaces[15], a characteristic that facilitates clean and reproducible structural release during the final mechanical lift-off step without damaging the underlying surface or the deposited thin films."

Revised Manuscript, Page 3, Line 13:

"We found that when printing the scaffold mask directly on a polymer surfaces, depositing a thin intermediate inorganic layer, such as 10 nm of sputtered SiO_2, is generally required, acting as a diffusion barrier and preventing strong covalent cross-linking and polymer chain interpenetration between the polymer surface and the IP-Dip resin[14]. This approach confines the lift-off mode of the scaffold mask to clean interfacial delamination rather than fracture within the scaffold itself. To evaluate the influence of different substrate surfaces and surface pre-treatments on the lift-off behavior of the scaffold mask, we performed comparative contact-angle and scaffold adhesion experiments and found that the surface material and its pre-treatment are important factors for reliable scaffold lift-off (Supplementary section 1)."

Revised Supplementary Information, Page 2, Line 1:

"**Influence of substrate surface and surface pre-treatment on scaffold mask lift-off**

To evaluate the influence of different substrate surfaces on the lift-off behavior of the scaffold structures, we compared representative interfaces with different pre-treatments and chemistries, including O_2 plasma-cleaned glass, SiO_2-coated glass, TPP-printed polymer (IP-Dip), and silanized glass (Figure S1). For each case, we examined both the water contact angle before scaffold deposition and the resulting lift-off behavior during sonication. O_2 plasma-cleaned and SiO_2-coated surfaces with low water contact angles of $\leq 10°$ enabled clean interfacial detachment of the scaffold, whereas silanized surface (contact angle of $\sim 50°$) and printed polymer surfaces (contact angle of $\sim 80°$) featured much stronger scaffold adhesion and led to fracture of the scaffold near its base during sonication, rather than clean removal of the scaffold from the surface. These results indicate that the scaffold lift-off mode is governed primarily by the interfacial adhesion state between the scaffold and the surface material, rather than by macroscopic wettability alone, which agrees with previous studies by Izard et al. and Jelinek et al[13,15]. The observed strong scaffold adhesion on silanized surface and polymer surfaces is consistent with stronger interfacial interactions, potentially including covalent anchoring in the case of silanized surfaces and polymer-polymer interpenetration or photochemically induced coupling in the case of printed polymer surfaces[14]. The resulting interfacial bond strength can exceed the cohesive strength of the scaffold material, shifting the lift-off mode from interfacial debonding between the scaffold and the substrate surface, to structural fracture of the scaffold material near the scaffold base. Therefore, on polymer surfaces we used a deposited thin intermediate SiO_2 layer before scaffold printing to ensure well-defined scaffold lift-off."

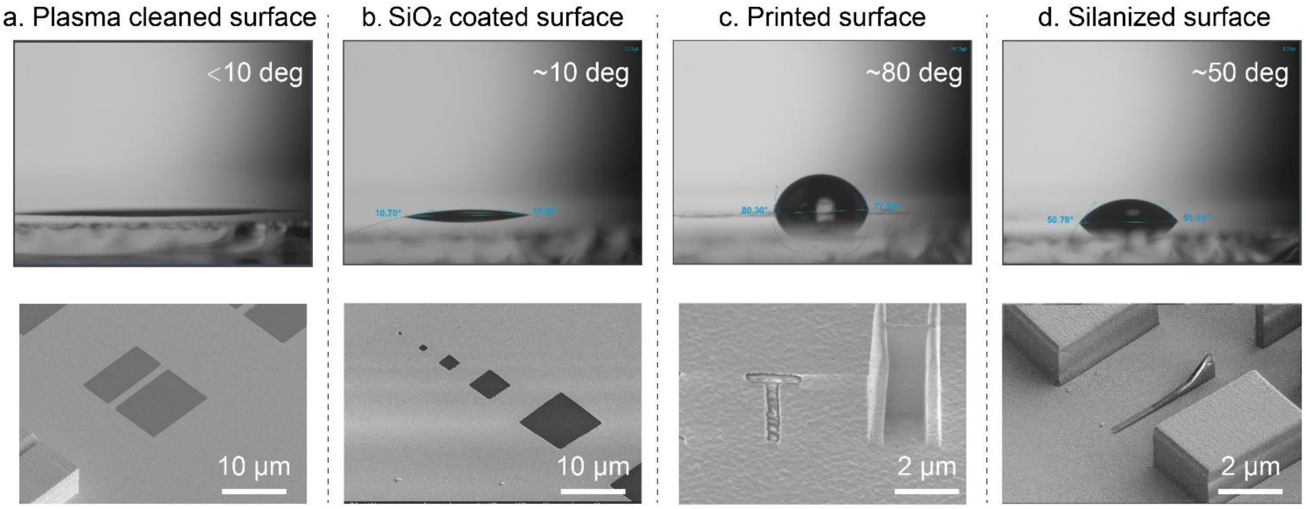

Figure S1. Correlation between substrate wettability and SALO behavior. Water contact angles were measured on four different surfaces based on ITO-coated glass substrate: (a) O_2 plasma-cleaned surface, <10°. (b) SiO_2-coated surface, ~10°. (c) 1 μm-thick TPP-printed polymer surface (IP-Dip), as printed, ~80°. (d) Silanized surface, ~50°. For (1) and (b), solid scaffolds with an aspect ratio (AR) of ~1 detached cleanly during sonication. For (c) and (d), solid scaffolds with an AR of ~1 did not release, while solid scaffolds with an AR of ~8 fractured at the base during sonication, instead of detaching from the substrate. Upper panels show the water droplets during contact-angle measurements, and lower panels show the corresponding state of the scaffolds after sonication. The features visible in the two lower-right panels are fractured scaffold remnants remaining on the substrate after lift-off.

2. Though micro- to nano-scaled patterns on 3D surfaces are claimed, judging from Figure 3, it is contradictory. Namely, the TPP scaffolds reach only 200 nm, whereas high-resolution sub-100 nm features are created by EBL on a flat surface. Thus, there is no direct evidence of 100 nm on conformal surfaces, as stated.

Our response:

We thank the reviewer for pointing out this inconsistency. We agree that, as shown in Figure 3, the sub 100 nm patterns are demonstrated on planar substrates using EBL, and we therefore revised the Manuscript text to avoid implying that arbitrary 100 nm features are demonstrated on conformal 3D surfaces. In this work, the minimum feature size on 3D surfaces is limited by the available 3D lithography and scaffold printing resolution. We do not currently have 3D patterning capabilities with sub-100 nm resolution on arbitrary 3D topographies. We therefore demonstrated EBL on flat substrates as a high-resolution reference case. In addition, we demonstrate a route to further reduce scaffold dimensions using oxygen plasma treatment, which is an established post processing strategy for downscaling structures printed using TPP. As shown in Figure 3g, the oxygen plasma processed scaffold on a 3D surface reaches sub-100 nm feature sizes. We now state this explicitly as a specific demonstrated case rather than a general claim of sub-100 nm conformal patterning across arbitrary 3D geometries.

To address this comment in the revised Manuscript and Supplementary Information, we revised the related text.

<u>Revised Manuscript, Page 6, Line 33:</u>

"It should be noted that the minimum feature size achievable by SALO depends on the patterning technique employed for realizing the scaffold mask. While electron beam lithography readily provides the possibility to realize scaffolds with sub-100 nm resolution on planar substrate surfaces, direct TPP printing of conformal

scaffolds is typically limited to structures with smallest dimensions of around 200 nm. However, as demonstrated in the example in Figure 3g, applying an oxygen plasma treatment to shrink the TPP printed scaffold mask enables lift-off patterning of sub-100 nm features on a 3D surface."

3. TPP, as an acronym, was introduced twice, but the parameters to replicate the experiment were not provided. Focusing conditions (used objective's NA) are missing, and the mentioned powers in mW do not reveal the actual intensity at the focus, which is essential for inducing the Two-Photon Absorption employed for Two-Photon Polymerization (TPP). Thus, it renders the experiment non-reproducible. It is clear within the community that the intensity is the essential factor determining the non-linear light-matter interaction, enabling 3D structuring, not the average power. This concept was explained in a tutorial paper in the field of Multiphoton 3D lithography. Nat Rev Methods Primers 5, 15 (2025). https://doi.org/10.1038/s43586-025-00386-y . Thus, the exposure conditions should be provided adequately.

Our response:

We thank the reviewer for this helpful comment and for pointing us to this tutorial paper, which we have now cited in the revised manuscript. We agree that our original description of the TPP exposure conditions was incomplete, and that reporting only the average laser power in mW without the key optical and scanning parameters limits reproducibility, as discussed in the cited tutorial.

To address this comment in the revised Manuscript, we have revised the Manuscript to (i) avoid introducing the TPP acronym twice and (ii) provide a complete set of writing parameters including the intensity in the Methods.

Revised Manuscript, Page 14, Line 14:

"The scaffold structures, both solid and shell-type, and the 3D polymer structures in this work were printed via two-photon lithography using a commercial laser direct writing system (Photonic Professional GT2, Nanoscribe GmbH) equipped with a 780 nm wavelength femtosecond laser. The laser operates at a repetition rate of 80 MHz with a pulse duration of ~100 fs. A 63× oil-immersion objective with a numerical aperture (NA) of 1.4 was used to focus the laser beam. According to the diffraction limit, the diffraction-limited spot size is ~0.34 μm (estimated as 0.61λ/NA). In this study, an laser power of 20 mW was applied, which corresponds to a pulse energy of ~0.25 nJ according to the irradiance calculation[26,27]. Consequently, the peak intensity (irradiance) at the center of the focal spot is estimated to be approximately 1.4 TW/cm^2."

4. Figure 4 has lots of information. But besides being visually nice, it's not very easy to extract the specific values for further use. Perhaps a table should be created to summarize the critical parameters, allowing the reader to easily and rapidly find the limits.

Our response:

We thank the reviewer for this excellent suggestion. We agree that Figure 4 is information dense and that extracting specific threshold values from the plot can be inconvenient.

To address this comment in the revised Manuscript and Supplementary Information, we have added Supplementary Table S1 that summarizes the critical parameters and observed limits.

Revised Manuscript, Page 8, Line 35:

"A summary of the critical detachment thresholds and geometric limits is provided in Supplementary Table S1 for rapid reference."

Revised Supplementary Information, Page 11, Line 1:

Table S1: SALO process results in dependence of scaffold mask aspect ratio (AR) and deposited thin film material and layer thickness of samples shown in Figure 4 and Figure S9.

Thin Film and Scaffold Features	Collapse AR	Lift-off AR	Non-releasable AR
100 nm Ag on circular pillar scaffold	7	0.6-6	< 0.6
20 nm Ag on circular pillar scaffold	11	0.5-10	< 0.5
20 nm Ta on circular pillar scaffold	17	0.6-15	< 0.6
100 nm Ag on square pillar scaffold	9	0.5-8	< 0.5
20 nm Ag on square pillar scaffold	17	0.5-16	< 0.5
20 nm Ta on square pillar scaffold	16	0.5-15	< 0.5
20 nm Ag on wall scaffold	-	> 0.4	< 0.4

5. Figure 5 shows impressive 3D structures used as sacrificial masks, but the produced multi-material structures are on flat or almost flat surfaces. Thus, though the proposed approach is transferable to multi-material deposition, it almost loses its 3D option (at least within the limitations of the study). This must be clearly explained, without overemphasizing that it can be applied to various materials. Since then, it has lost its 3D capacity (not yet proven).

Our response:

We sincerely thank the reviewer for this critical observation. The reviewer correctly points out that while our sacrificial masks is 3D, the initial demonstration of multi-material patterning in Figure 5 was performed on essentially planar substrates, which did not fully prove the 3D capacity of our method in this example.

To address this limitation, we have conducted new experiments and successfully demonstrated multi-material SALO patterning directly on a non-planar 3D substrate. We have updated Figure 5 to include these new results (selective lift-off result in Figure 5c, d, e and f), providing concrete experimental proof that the multi-material capability is fully compatible with 3D topographies. We have also retained the original planar ALD example in Figure 5a, as it remains the best way to quantitatively characterize and compare the conformal coating thickness and uniformity.

To address this comment in the revised Manuscript, we present the 3D multi-material demonstration in the new Figure 5 and clarified the corresponding text accordingly.

<u>Revised Manuscript, Page 10, Line 16:</u>

“To demonstrate selective SALO on 3D topographies, we realized a mask with different types of scaffolds that release at different ultrasonic power thresholds (Figure 5c, d).”

<u>Revised Manuscript, Page 11, Line 1:</u>

<u>New Figure 5:</u>

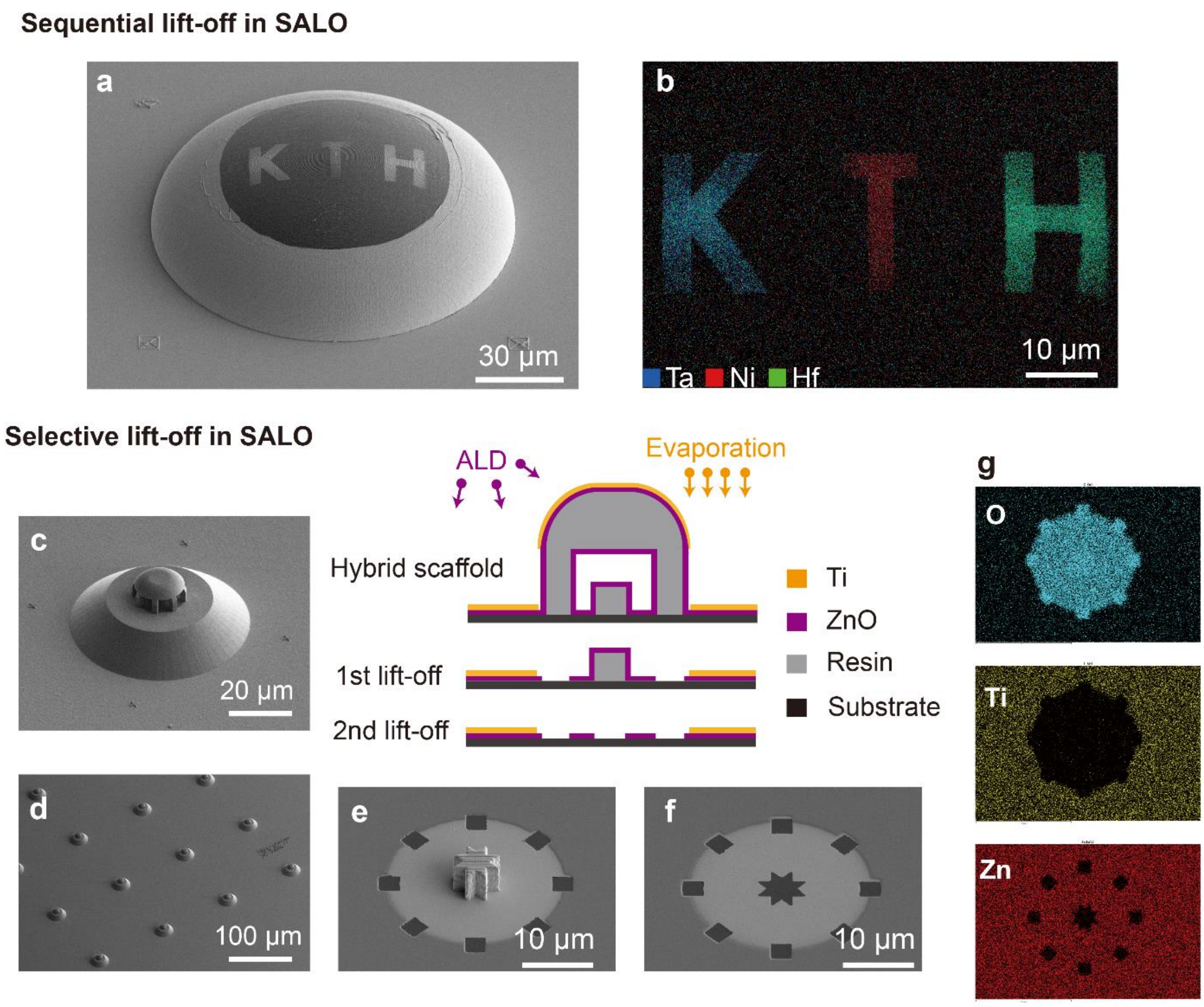

Original Figure 5:

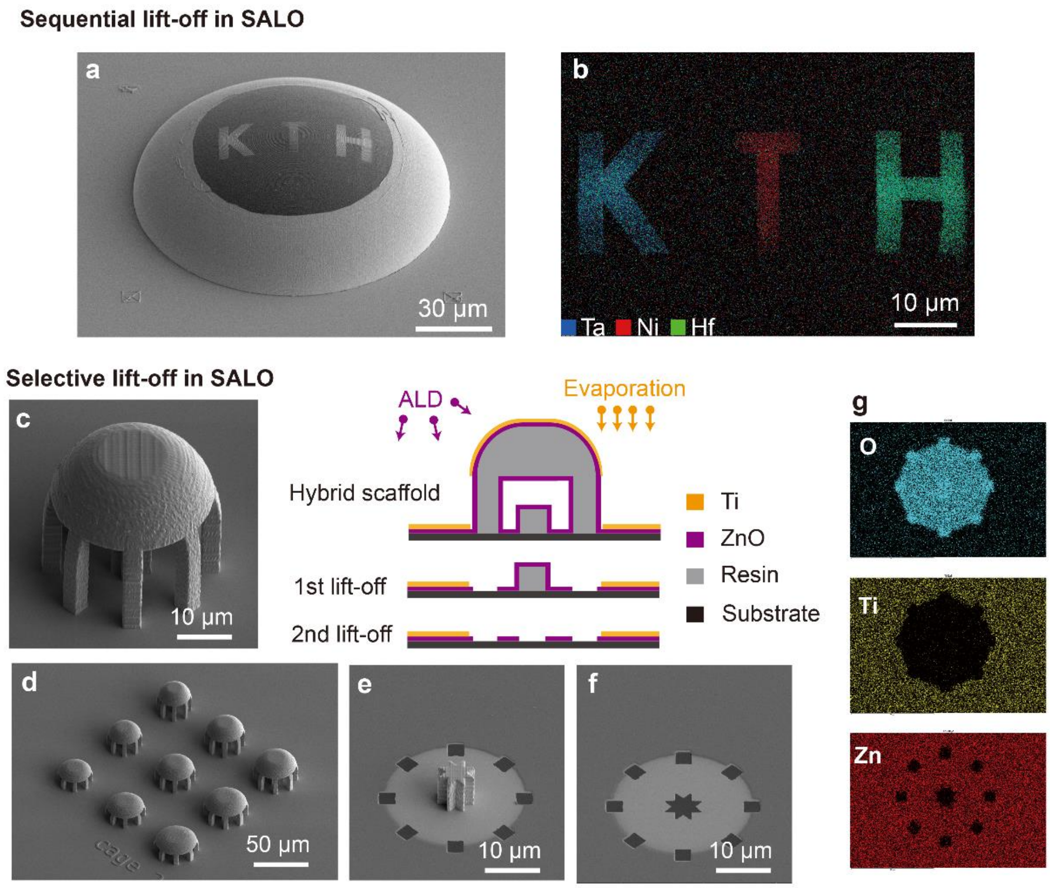

6. ALD has been used in combination with TPP for micro-optical applications, showing its excellent performance as anti-reflective coatings, for instance: Anti-Reflective Coatings Produced via Atomic Layer Deposition for Hybrid Polymer 3D Micro-Optics, Nanomaterials 13(16), 2281 (2023); https://doi.org/10.3390/nano13162281. Authors could consider discussing it and also foreseeing if the SALO technique could be beneficial for optics/photonics. Importantly, in that case, hybrid materials were used, which can withstand higher temperatures and are more mechanically rigid.

Our response:

We thank the reviewer for this helpful suggestion and for pointing us to this highly relevant reference. We fully agree that the combination of ALD and TPP has shown excellent performance for micro-optical applications, such as anti-reflective coatings on 3D micro-optics. In the revised Manuscript, we have integrated a related discussion. We highlight how SALO is highly complementary to these uniform coating techniques, as it enables the spatially selective patterning of conformal films on complex optical surfaces. Furthermore, we agree with the important point of the reviewer regarding the scaffold material choice. We have added a forward-looking statement noting that utilizing hybrid organic-inorganic materials (as employed in the suggested reference) for the SALO sacrificial scaffolds would provide enhanced thermal and mechanical rigidity, which is attractive for widening process window for the scaffold masks.

To address this comment in the revised Manuscript, We have added a discussion highlighting the potential relevance of SALO for future optics and photonics applications and potential use of hybrid organic-inorganic photoresists.

Revised Manuscript, Page 12, Line 12:

"Beyond this specific demonstration, 3D photonics appears to be a particularly promising application space for SALO; for instance, ALD has previously been integrated with 3D printed micro-optics to produce highly conformal anti-reflective coatings without compromising the underlying surface topography[19]. Combined with SALO, this conformal coating capability could enable the fabrication of more advanced 3D photonic architectures."

Revised Manuscript, Page 13, Line 28:

"To accommodate broader process windows (e.g., increased deposition temperatures), the organic photoresist for the scaffold mask may be replaced by hybrid organic-inorganic photoresists, which have been shown to offer improved thermal and mechanical rigidity[28,29].

7. Could the approach be further expanded by utilizing post-processing solutions, such as thermal annealing (calcination or pyrolysis), to transform the scaffolds into inorganic structures and increase their resilience to etching or lift-off? See a report on that: Laser additive manufacturing of Si/ZrO_2 tunable crystalline phase 3D nanostructures. Opto-Electron. Adv. 5, 210077 (2022); https://doi.org/10.29026/oea.2022.210077.

Our response:

We thank the reviewer for this interesting suggestion and for pointing us to this highly relevant reference. We completely agree that integrating post-processing techniques like pyrolysis represents an interesting strategy to expand the capabilities and resilience of our SALO approach. This could unlock at least two new capabilities for the SALO process:

1. Compatibility with Extreme Deposition Conditions: Currently, SALO relies on low-to-moderate temperature processes (such as ALD) to preserve the polymer scaffold mask. A pyrolyzed, inorganic scaffold could withstand much harsher environments, enabling conformal lift-off of high-temperature

CVD or PVD films (e.g., refractory metals or high-quality nitrides) that would otherwise melt or degrade standard polymeric photoresists.

2. Surpassing Optical Resolution Limits: Pyrolysis induces significant and isotropic shrinkage of the 3D scaffold. Leveraging this volumetric shrinkage before thin-film deposition could effectively scale down the SALO mask dimensions below the optical diffraction limit of the TPP system.

To address this comment in the revised Manuscript, we have added a brief discussion and relevant citation in the revised manuscript as follows:

Revised Manuscript, Page 13, Line 30:

"Building on this, the SALO process could be further expanded by incorporating thermal treatments, such as calcination or pyrolysis[27], of the 3D scaffold mask. This may enable more thermally robust scaffold architectures, provide a route towards further improving the achievable scaffold mask resolution, and facilitate the realization of multi-compositional 3D microstructures."

8. Is the approach applicable to all substrates? Or is it limited to some, and what are the criteria for it?

Our response:

We thank the reviewer for this question. We agree that it is critical to define the requirements on the substrate surface. Important aspects in the SALO process are the (1) TPP printing process, (2) the adhesion between the scaffold material and the substrate surface, and (3) the subsequent lift-off processes:

1. Optical compatibility of substrate with 2PP printing: The substrate must allow precise laser focusing. Highly reflective surfaces (which cause severe interference and voxel distortions) can locally modify the exposure conditions and generate printing artifacts (new Supplementary Figure S15b).
2. Adhesion between substrate surface and scaffold mask: Excessively strong adhesion between the substrate and the scaffold mask can shift the lift-off mode from interfacial scaffold debonding to structural fracture of the scaffold itself (Supplementary Figure S1).
3. Mechanical robustness during lift-off: The substrate surface must be sufficiently stable to withstand the ultrasonic agitation required for the lift-off process. This is also true for deposited thin film, which has to have sufficient adhesion to the substrate to avoid thin-film delamination during the lift-off process (new Supplementary Figure S15a).

We also would like to point out that we have successfully demonstrated SALO on a range of substrate surfaces in this work, including silicon 3D structures, glass dome surfaces, 3D printed polymer structures, and fiber end facets, which supports broad substrate compatibility. Special cases such as soft polymers, highly porous substrates, or extremely rough surfaces have not yet been systematically evaluated.

To address this comment in the revised Manuscript and Supplementary Information, we have clarified the constraints and material dependencies of the SALO process in the Discussion section. We also have included new experimental results and analysis of the role of the adhesion between the substrate surface and the scaffold mask, and we have summarized potential failure modes of the SALO process in the new Supplementary Figure 15.

Revised Manuscript, Page 13, Line 16:

"In this work, we successfully demonstrated the SALO process on a diverse range of substrates, including silicon surfaces with planar and 3D topographies, glass domes, TPP-printed 3D polymer structures, and optical glass fiber facets. While this highlights the broad applicability of the SALO patterning process, the technique

is naturally constrained by the physical requirements of the TPP printing and lift-off workflows. Specifically, the TPP printing relies on precise laser focusing, meaning that substrates with extreme optical properties, such as highly reflective metal or excessively rough surfaces, can cause laser light scattering or interference artifacts that locally modify the exposure dose during TPP printing. Additionally, the substrate surface must possess sufficient mechanical robustness to withstand the ultrasonic agitation required during the lift-off process. As a result, applying SALO to highly reflective or rough surfaces, or to unconventional or fragile materials like soft polymers or highly porous substrates, will require process optimization. A summary of potential failure modes arising from poor adhesion of the deposited thin films, reflection-induced exposure non-uniformity when TPP printing the scaffold mask, and incomplete resist development of the TPP-printed scaffold mask is provided in Supplementary section 16 and Figure S15."

Revised Supplementary Information, Page 2, Line 1:

"Influence of substrate surface and surface pre-treatment on scaffold mask lift-off

To evaluate the influence of different substrate surfaces on the lift-off behavior of the scaffold structures, we compared representative interfaces with different pre-treatments and chemistries, including O_2 plasma-cleaned glass, SiO_2-coated glass, TPP-printed polymer (IP-Dip), and silanized glass (Figure S1). For each case, we examined both the water contact angle before scaffold deposition and the resulting lift-off behavior during sonication. O_2 plasma-cleaned and SiO_2-coated surfaces with low water contact angles of ≤10° enabled clean interfacial detachment of the scaffold, whereas silanized surface (contact angle of ∼50°) and printed polymer surfaces (contact angle of ∼80°) featured much stronger scaffold adhesion and led to fracture of the scaffold near its base during sonication, rather than clean removal of the scaffold from the surface. These results indicate that the scaffold lift-off mode is governed primarily by the interfacial adhesion state between the scaffold and the surface material, rather than by macroscopic wettability alone, which agrees with previous studies by Izard et al. and Jelinek et al[13,15]. The observed strong scaffold adhesion on silanized surface and polymer surfaces is consistent with stronger interfacial interactions, potentially including covalent anchoring in the case of silanized surfaces and polymer-polymer interpenetration or photochemically induced coupling in the case of printed polymer surfaces[14]. The resulting interfacial bond strength can exceed the cohesive strength of the scaffold material, shifting the lift-off mode from interfacial debonding between the scaffold and the substrate surface, to structural fracture of the scaffold material near the scaffold base. Therefore, on polymer surfaces we used a deposited thin intermediate SiO_2 layer before scaffold printing to ensure well-defined scaffold lift-off."

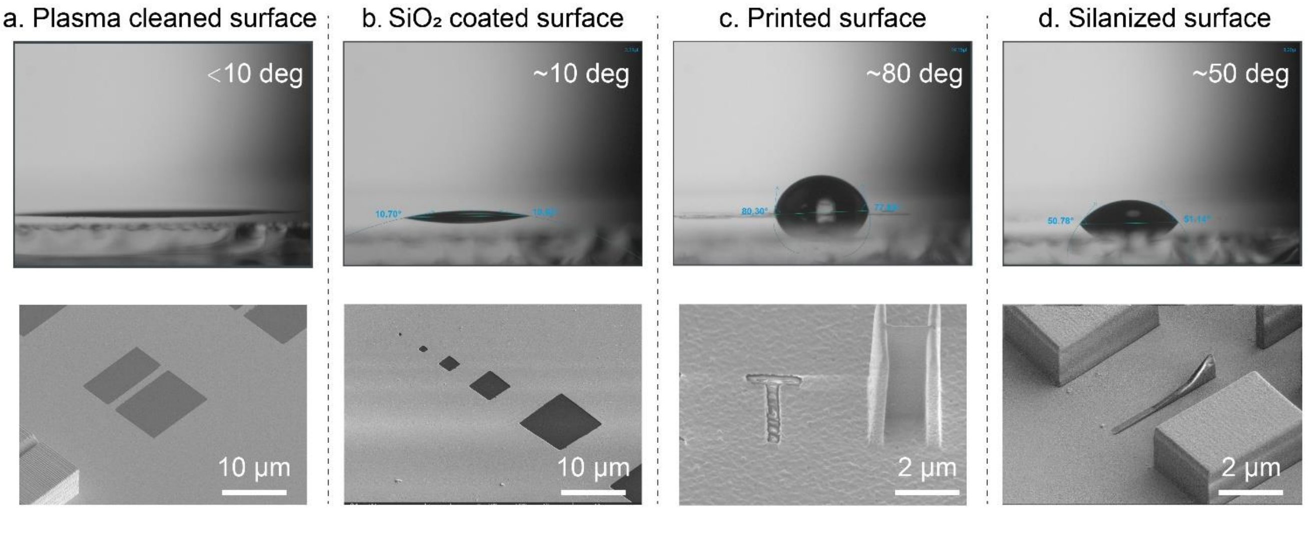

Figure S1. Correlation between substrate wettability and SALO behavior. Water contact angles were measured on four different surfaces based on ITO-coated glass substrate: (a) O_2 plasma-cleaned surface, <10°. (b) SiO_2-coated surface, ∼10°. (c) 1 μm-thick TPP-printed polymer surface (IP-Dip), as printed, ∼80°. (d)

Silanized surface, ~50°. For (1) and (b), solid scaffolds with an aspect ratio (AR) of ~1 detached cleanly during sonication. For (c) and (d), solid scaffolds with an AR of ~1 did not release, while solid scaffolds with an AR of ~8 fractured at the base during sonication, instead of detaching from the substrate. Upper panels show the water droplets during contact-angle measurements, and lower panels show the corresponding state of the scaffolds after sonication. The features visible in the two lower-right panels are fractured scaffold remnants remaining on the substrate after lift-off.

Revised Supplementary Information, Page 22, Line 1:

"**SALO process failure modes**

Summary of three representative SALO failure mechanisms and mitigation strategies

1. Thin Film Delamination

- Mechanism & Limitation: When depositing metals with weak surface adhesion directly onto certain inert substrates, such as the sputtered Au films shown here, the interfacial adhesion energy between the Au layer and the substrate is often overcome by the internal stress of the thin film and/or the mechanical force applied during the ultrasonic lift-off process. This can result in delamination or tearing of the deposited thin film at the pattern edges (Figure S15a).
- Mitigation: This is a fundamental issue relating to thin film adhesion rather than specific to the SALO process. This failure mechanism is readily mitigated by optimized the thin film deposition process, such as the use of an adhesion layer (e.g., 2-5 nm of Ti or Cr) prior to depositing the metal layer (here Au).

2. Laser Exposure Dose Non-uniformity Due to Reflective Surfaces During Scaffold Printing

- Mechanism & Limitation: Two-photon polymerization (TPP) is sensitive to local light intensities. When printing on reflective substrate surfaces, such as reflective metal films, the focused laser beam interferes with laser light reflected by the substrate surface, creating standing waves and localized intensity hotspots at the substrate interface. This proximity effect can induce unintended over-crosslinking or "scalloping" of the photoresist near the base of the scaffold, which can severely distort and compromise the scaffold geometry (Figure S15b). This effect limits the applicability of SALO on reflective mirror-like surfaces if no mitigation measures are taken.
- Mitigation: The optical interferences caused by reflective surfaces can be mitigated by applying a thin anti-reflective coating (ARC)[18,19] prior to TPP printing, or by implementing adaptive exposure strategies that dynamically reduce the laser power precisely at the substrate interface to compensate for the energy of the reflected laser light.

3. Incomplete Development of the Photoresist Near the Base of the Scaffold

- Mechanism & Limitation: Incomplete photoresist development can result in non-crosslinked photoresist residues trapped near the base of the scaffold (i.e., the interface critical for the SALO process). The resist residues locally prevent coverage of the substrate surface with the deposited thin film, thereby compromising pattern integrity (Figure S15c). Incomplete resist development can, for example, be caused by restricted transport of the liquid developer (e.g., PGMEA) in scaffolds with high aspect-ratio or dense geometries.
- Mitigation: This limitation can be overcome by optimizing the resist development protocol. Specifically, extending the development time or introducing gentle agitation of the developer bath can improve removal of unexposed photoresist from dense, high-aspect-ratio regions.

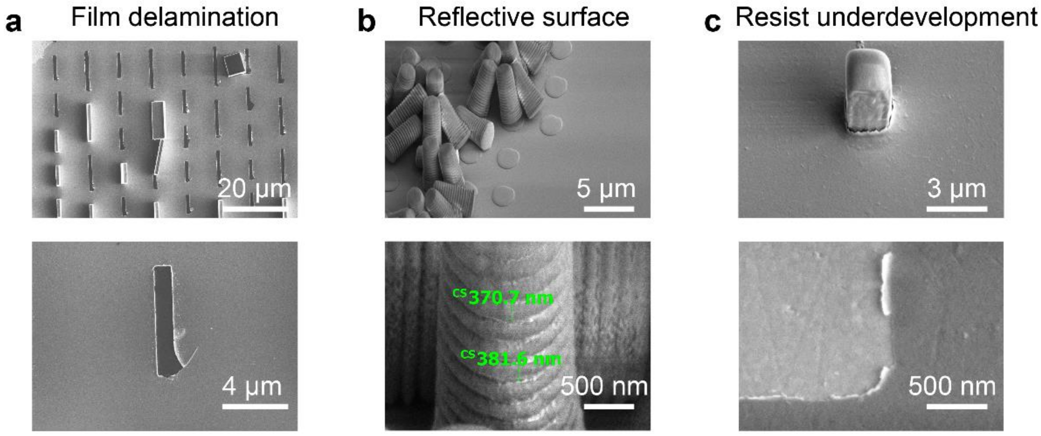

Figure S15 | Possible failure modes and their causes. a| Representative example of observed delamination of a SALO-patterned 25 nm thick sputtered Au film without Ti or Cr adhesion layer. b| Effects of exposure dose non-uniformity near the scaffold base during scaffold TPP printing caused by reflections from reflective substrate surface (here sputtered 20 nm Cr), which can compromise the scaffold mask (upper image) by unintended photoresist cross-linking or scalloping (lower image). c| Example of incomplete scaffold-mask development leaving resist residues at the scaffold base (here after sputtering 20 nm of Cr). The resist residues can shadow the thin film deposition (upper image) and cause residual flakes at the edges of the patterned film after the SALO process (lower image).

9. 14 mW UV flood exposure sounds unreasonably low. Is it true, or what exactly do these numbers in mW mean?

We thank the reviewer for spotting this error and sincerely apologize for the typo in the previous version of the Manuscript. The value we intended to report is the irradiance (intensity) at the sample plane, which was measured to be 14 mW/cm². Consequently, a 15-minute (900 seconds) UV flood exposure corresponds to a total exposure dose of 12.6 J/cm² (calculated as 14 mW/cm² × 900 s). We have corrected this error in the revised Manuscript and updated the Methods section to explicitly state the irradiance in mW/cm² and the calculated total dose in J/cm².

To address this comment in the revised Manuscript, we have corrected this error in the revised Manuscript and included the specific irradiance and total exposure dose to provide a more precise process description.

Revised Manuscript, Page 14, Line 39:

"A final UV flood exposure of 15min (irradiance at the sample plane was measured to be 14 mW/cm², corresponding to a total exposure dose of 12.6 J/cm²) ensured complete polymerization and solvent removal."

10. In Supplement's Figure 1, the shape of curved surfaces is different, why? It seems it was supposed to be a parallel comparison, but it its misleading. Also, what is intended to show with the resin mask is that it is possible to combine the removed and non-removed parts by adjusting the AR of the structures? It should be elaborated more on that for easier understanding.

Our response:

We thank the reviewer for raising this point and we agree that, if presented as a strictly parallel comparison, differences in curvature could be misleading. In the revised Supplementary Information, we clarify that the dome-like Si curvatures in Supplementary Figure were intentionally chosen as representative examples

of different substrate topographies rather than as a one-to-one comparison of a specific curvature 3D surface. We have revised the text to clarify that the curvature of the silicon surface cannot be easily defined in an arbitrary way, and we explicitly discuss the practical limitations of realizing 3D silicon topographies that influence the final curvature of the silicon surface.

Regarding the “resin mask”, we have expanded the explanation to state that, by adjusting the aspect ratio of the scaffold features, we can tune the scaffold structures to preferentially be removed or retained during the lift-off process, thereby enabling deliberate coexistence of removed and non-removed scaffold structures within the same scaffold mask design.

To address this comment in the revised Manuscript and Supplementary Information, we have added a discussion to the revised Supplementary Information clarifying why the dome-shaped silicon substrates exhibit different curvatures compared to the 3D-printed models. We also adjusted the text in the Manuscript to avoid the impression that the Si and polymer surfaces are one-to-one comparisons.

Revised Manuscript, Page 4, Line 23:

“To further demonstrate these capabilities, we patterned the United Nations logo onto substrates with curved surfaces (fabrication details in Methods–Preparation of 3D substrate), including on TPP printed curved polymer structures, and on curved silicon surfaces (Supplementary Figure S2). ”

Revised Supplementary Information, Page 3, Line 9:

“The dome-like Si surfaces shown in Supplementary Figure S2 were fabricated by photoresist reflow followed by pattern-transfer from the resist to the silicon surface using inductively coupled plasma reactive ion etching (ICP-RIE) (see Methods for fabrication details). As a result, the design freedom of the curvature of the resulting silicon surface is limited by process-dependent factors such as the photoresist reflow behavior and the subsequent dry etching selectivity and etch transfer characteristics between the photoresist and the silicon. Therefore, the silicon domes in Supplementary Figure S2b are not one-to-one curvature-matched counterparts to the TPP printed polymer dome structures shown in Figure S2d.”

All in all, the Manuscript is relatively good, but must be further improved in order to fit the level of the chosen journal.

Our response:

We appreciate the positive assessment our work and the constructive feedback provided by the reviewer. We have carefully addressed all specific points raised, and we believe these revisions have significantly improved the quality of the Manuscript, making it now well-suited for *Nature Communications*.

References

1. Mhanna, R. *et al.* Artificial Bacterial Flagella for Remote-Controlled Targeted Single-Cell Drug Delivery. *Small* **10**, 1953–1957 (2014).
2. Liao, Y. *et al.* 3D-Printed Complex Microstructures with a Self-Sacrificial Structure Enabled by Grayscale Polymerization and Ultrasonic Treatment. *ACS Omega* **6**, 18281–18288 (2021).
3. Weber, J. C., Brubaker, M. D., Wallis, T. M. & Bertness, K. A. Lithographic sonication patterning of large area GaN nanopillar forests grown on a Si substrate. *Microelectronic Engineering* **181**, 43–46 (2017).
4. Biercuk, M. J., Monsma, D. J., Marcus, C. M., Becker, J. S. & Gordon, R. G. Low-temperature atomic-layer-deposition lift-off method for microelectronic and nanoelectronic applications. *Applied Physics Letters* **83**, 2405–2407 (2003).
5. Yu, J. H., Cho, Y., Lee, J., Choi, K. H. & Lee, S. Area-selective atomic layer deposition of Al2O3 using inkjet-printed inhibition patterns and lift-off process. *Journal of Information Display* **24**, 215–225 (2023).
6. Lamminen, M. O., Walker, H. W. & Weavers, L. K. Mechanisms and factors influencing the ultrasonic cleaning of particle-fouled ceramic membranes. *Journal of Membrane Science* **237**, 213–223 (2004).
7. Jia, W. *et al.* Numerical simulation study on the characteristics and mechanism of ultrasonic cavitation cleaning of crude oil sediments. *Ultrason Sonochem* **122**, 107583 (2025).
8. Plesset, M. S. & Chapman, R. B. Collapse of an initially spherical vapour cavity in the neighbourhood of a solid boundary. *J. Fluid Mech.* **47**, 283–290 (1971).
9. Lu, X., Chen, C., Dong, K., Li, Z. & Chen, J. An equivalent method of jet impact loading from collapsing near-wall acoustic bubbles: A preliminary study. *Ultrason Sonochem* **79**, 105760 (2021).
10. Ohl, C.-D. *et al.* Sonoporation from jetting cavitation bubbles. *Biophys J* **91**, 4285–4295 (2006).
11. Sankin, G. N., Yuan, F. & Zhong, P. Pulsating Tandem Microbubble for Localized and Directional Single Cell Membrane Poration. *Phys Rev Lett* **105**, 078101 (2010).
12. Kim, W., Kim, T.-H., Choi, J. & Kim, H.-Y. Mechanism of particle removal by megasonic waves. *Appl. Phys. Lett.* **94**, 081908 (2009).
13. Izard, A. G. *et al.* Enhanced adhesion in two-photon polymerization direct laser writing. *AIP Advances* **10**, 045217 (2020).
14. Han, J. Y., Warshawsky, S. & DeVoe, D. L. In situ photografting during direct laser writing in

thermoplastic microchannels. *Sci Rep* **11**, 10980 (2021).

15. Jelinek, A., Rossegger, E., Schlögl, S., Kiener, D. & Alfreider, M. Probing local adhesion: A miniaturized multi-photon lithography design demonstrated on silanized vs. untreated surfaces. *Materials & Design* **242**, 112994 (2024).
16. Greczynski, G. & Hultman, L. X-ray photoelectron spectroscopy: Towards reliable binding energy referencing. *Progress in Materials Science* **107**, 100591 (2020).
17. Hantsche, H. High resolution XPS of organic polymers, the scienta ESCA300 database. By G. Beamson and D. Briggs, Wiley, Chichester 1992, 295 pp., hardcover, £ 65.00, ISBN 0-471-93592-1. *Advanced Materials* **5**, 778–778 (1993).
18. Park, E.-M. *et al.* Investigation of the effects of bottom anti-reflective coating on nanoscale patterns by laser interference lithography. *Thin Solid Films* **519**, 4220–4224 (2011).
19. Astrauskytė, D. *et al.* Anti-Reflective Coatings Produced via Atomic Layer Deposition for Hybrid Polymer 3D Micro-Optics. *Nanomaterials* **13**, (2023).
20. Enrico, A., Dubois, V., Niklaus, F. & Stemme, G. Scalable Manufacturing of Single Nanowire Devices Using Crack-Defined Shadow Mask Lithography. *ACS Appl Mater Interfaces* **11**, 8217–8226 (2019).
21. Qin, H. *et al.* In-fiber single-polarization diffraction grating based on radiant tilted fiber grating. *Opt. Lett., OL* **44**, 4407–4410 (2019).
22. Geng, Q., Wang, D., Chen, P. & Chen, S.-C. Ultrafast multi-focus 3-D nano-fabrication based on two-photon polymerization. *Nat Commun* **10**, 2179 (2019).
23. Ouyang, W. *et al.* Ultrafast 3D nanofabrication via digital holography. *Nat Commun* **14**, 1716 (2023).
24. Gu, S. *et al.* 3D nanolithography with metalens arrays and spatially adaptive illumination. *Nature* **648**, 591–599 (2025).
25. Bauer, J., Guell Izard, A., Zhang, Y., Baldacchini, T. & Valdevit, L. Programmable Mechanical Properties of Two-Photon Polymerized Materials: From Nanowires to Bulk. *Advanced Materials Technologies* **4**, 1900146 (2019).
26. Skliutas, E. *et al.* Multiphoton 3D lithography. *Nat Rev Methods Primers* **5**, 15 (2025).
27. Merkininkaitė, G., Aleksandravičius, E., Malinauskas, M., Gailevičius, D. & Šakirzanovas, S. Laser additive manufacturing of Si/ZrO_2 tunable crystalline phase 3D nanostructures. *OEA* **5**, 210077–11 (2022).

28. Navickas, M. *et al.* Donor–acceptor complexes between photoinitiators and hybrid organic–inorganic SZ2080^{TM} photoresist. *Materials Advances* https://doi.org/10.1039/D5MA01526J (2026) doi:10.1039/D5MA01526J.

29. Stavrou, M. *et al.* Direct measurement of two-photon absorption and refraction properties of SZ2080TM-based resists at 515 nm: insights into 3D printing. *Nanophotonics* **14**, 2981–2992 (2025).

Point-by-Point Response to the Comments of the Reviewers

We thank the referees for the constructive comments. With the clarifications and the detailed responses to all reviewer comments provided below, we believe the manuscript now is suitable for publication in Nature Communications.

Reviewer #1

I don't think the author well response my question, and the innovation of the present study is not well demonstrated compared to those previous papers.

As this reviewer does not specify the exact question and papers that he/she is referring to, we unfortunately cannot provide a specific response. We agree with reviewers #3 and #4, and believe that the novelty and innovation of the present study have been clearly demonstrated in our revised manuscript.

Reviewer #3

The substantially revised version adequately addresses the raised questions.

We thank the reviewer for the positive evaluation of our revised manuscript.

Reviewer #4

This reviewer read the revised manuscript first, before looking at the previous reviewers' reports, and author's reply/revision. The approach produces a pre-designed 3D Scaffold-Architected Lift-Off (SALO) mask on designated surfaces, then deposits desired materials, and finally lifts the mask. The outcomes are the negatives defined by the mask. The resultant films are conformal to the mask design, with impressive patterns on sloped surfaces with angles up to 90°. The multiscale dimensions from 0.1 mm to tens of nm scale are truly impressive. In my opinion, two aspects of this work warrant a publication on Nature Communication: (a) the outcomes of their lithography, which has high spatial accuracy crossing multidimensions, from nano-, meso-, to macro-size; (b) the science opportunities this report would generate for peers. At the end of the day, the quality of data and thought-provoking work comprises the type of paper that should reach the readers of Nature.

The two reviewers before me covered most of the science and technological issues already. The authors made a comprehensive and good faithed effort to address them point-by-point. I found the replies and revisions to be satisfactory. I recommend the following to improve the broader impacts to benefit Nature's readers.

We thank the reviewer for the efforts in evaluation all documents, and for the positive evaluation of our work and the revised manuscript.

(1) The concept of "3D surface" is technically mis-used. By stretching, they can be called "supported 3D surfaces" or better yet, they should be referred to as "exterior surfaces of 3D structures."
Our response:

We thank the reviewer for pointing out this incorrect terminology. We fully agree that the term "3D surface"

is technically imprecise.

To address this comment in the revised Manuscript, we now refer to the studied geometry at first mention as the "exterior surfaces of 3D structures" and have revised the terminology in the relevant parts of the manuscript accordingly.

(2) The thickness of the films, especially among those designed structures, should be accurately measured, e.g., using atomic force microscopy (AFM). It may not be the same as that measured during deposition using QCM.

We thank the reviewer for this helpful suggestion. In response, we have added an AFM step-height measurement of the thinnest SALO-patterned sample (nominally 15 nm ALD HfO_2) as an additional validation of the patterned film thickness. We note that AFM was not performed for every patterned thickness. Instead, the film thicknesses reported in this work were calibrated independently using reference structures measured by a stylus profiler, rather than taken directly from the deposition tool readout. For samples patterned on 3D structures, reference structures were fabricated outside the 3D region specifically for thickness calibration. The AFM result on the thinnest patterned sample therefore serves as an additional confirmation that the SALO-patterned film thickness is consistent with the nominal calibrated thickness.

To address this comment, we have added AFM measurement results for the deposited ALD film in the revised **Supplementary Information** and the corresponding text in the revised **Manuscript**. We also have revised the order of Fig. 2b and Fig.2d to match the sequence of their appearance in the main text.

Revised Manuscript, Page 6, Line 1:

"To further confirm the actual thickness of a patterned ALD layer we performed step-height measurements by atomic force microscopy (AFM) of the thinnest representative ALD layer (15 nm of HfO_2) that we patterned using SALO, confirming its nominal thickness (Supplementary Fig. S6c)."

Old Fig2:

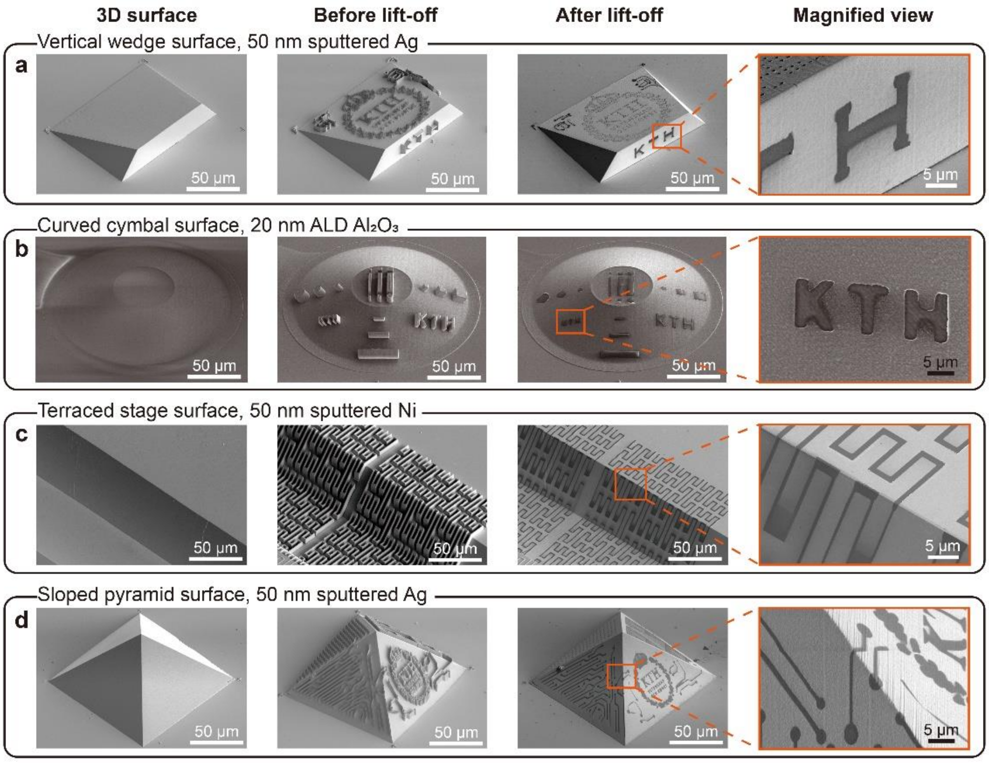

new Fig2:

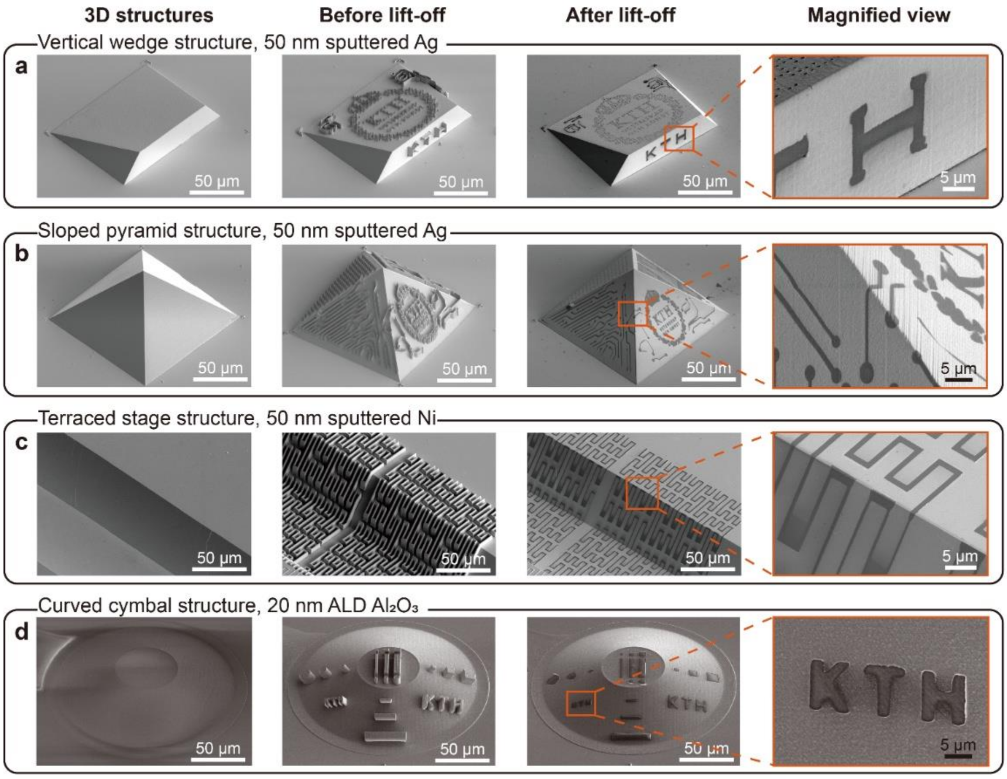

Revised Supplementary Information, Page 7, Line 7:

"AFM topography and line-profile analysis of a sample corresponding to the thinnest ALD layer (15 nm thick HfO2) we have patterned by SALO, further confirming that the patterned ALD layer thickness was consistent with the nominal thickness (Fig. S6c).

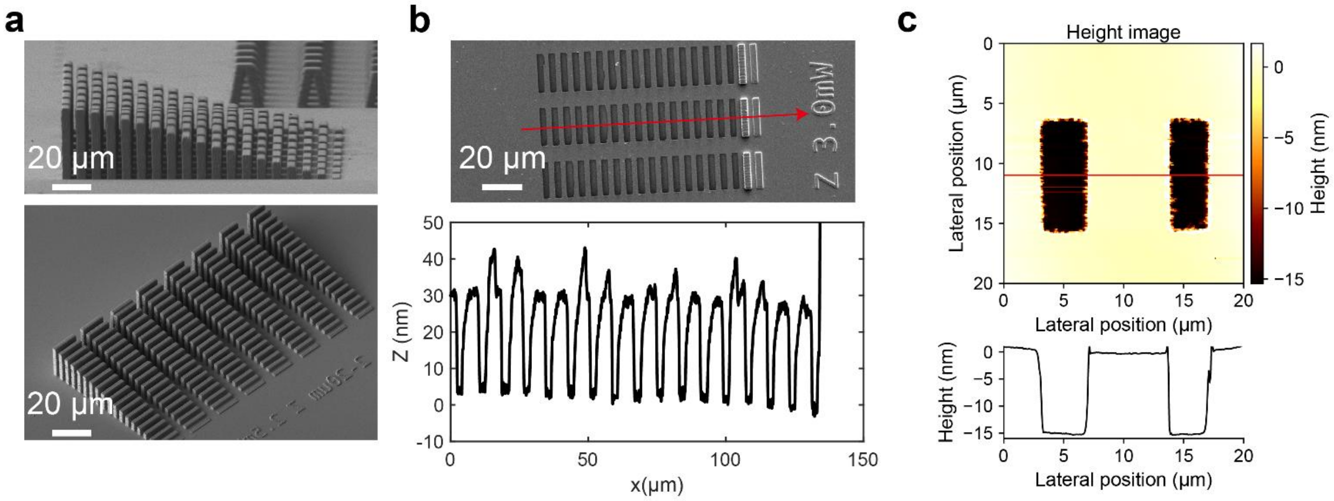

Figure S6 | High-aspect-ratio scaffold geometry and conformal film coverage. a | SEM images of representative high-aspect-ratio scaffolds fabricated by TPP printing. **b** | Top-view SEM of SALO-patterned 25 nm ZnO thin film which was deposited inside the deep trench of the very-high aspect ratio scaffolds. The red arrow indicates the line-scan direction and the corresponding height profile, showing a constant ALD coating thickness of about 25 nm across the gaps that were surrounded by scaffolds with different aspect ratios. The spikes in the line-scan are due to particles at the surface (confirmed by SEM imaging) that were present before the ALD layer was deposited. **c** | AFM thickness measurement of the SALO-patterned 15 nm thick ALD HfO2 layer, used as a representative sample to verify the actual ALD layer thickness."

(3) SiN or Si3N4? I suspect it was the latter.

We thank the reviewer for this comment. We have corrected this and replaced "SiN" with "Si_3N_4" throughout the Methods section.

(4) Discussion and Method cited no references. When past work is mentioned, citing the representative references would be appropriate.

We thank the reviewer for this comment. We have now added appropriate representative references in both the Discussion and Methods sections wherever prior work is mentioned.

(5) Minor editorial issues, there should be a space between a number and its unit, e.g., 30 nm not 30nm. The manuscript uses them both somewhat randomly, e.g., in Figure 3.

We thank the reviewer for pointing this out. We have corrected this formatting issue and carefully reviewed the entire manuscript to ensure consistent spacing between numbers and units throughout.

Overall, the work is impressive and thought-provoking. I recommend publication after the above points are addressed.

We thank the reviewer for the positive evaluation of our work.
